# First-day lactate trajectories and subsequent in-hospital mortality in cardiogenic shock: a landmark multicohort study

**DOI:** 10.3389/fcvm.2026.1948425

**Published:** 2026-09-09

**Authors:** Xiaoye Xu, Tielong Chen, Fei Ying, Huili Zhou, Zhao Liu, Ping Wang, Pengqin Shu

**Affiliations:** 1 Affiliated Mental Health Center & Hangzhou Seventh People's Hospital, Zhejiang University School of Medicine, Hangzhou, Zhejiang, China; 2 Department of Cardiology, Hangzhou TCM Hospital Affiliated with Zhejiang Chinese Medical University, Hangzhou, Zhejiang, China

**Keywords:** cardiogenic shock, eICU-CRD, intensive care, lactate, landmark analysis, MIMIC-IV, mortality, trajectory analysis

## Abstract

**Background:**

Lactate in cardiogenic shock is commonly summarized by a single measurement or a two-point estimate of clearance, although its first-day pattern may offer a more interpretable account of early clinical evolution. We examined whether first-day lactate trajectories identify reproducible patterns associated with subsequent mortality.

**Methods:**

This retrospective multicohort study used MIMIC-IV for trajectory discovery and internal prediction modeling and eICU-CRD to evaluate transportability. Adults with a first ICU admission for cardiogenic shock and at least two lactate measurements within 24 h were eligible for trajectory construction. Primary analyses were restricted to patients alive and still hospitalized at a 24-hour landmark.

**Results:**

Four patterns were identified among 2,181 eligible MIMIC-IV patients: low-stable, moderate-decreasing, high-decreasing, and persistent-high. The 24-hour landmark risk set included 2,015 patients, with subsequent mortality ranging from 24.34% to 72.88%. Compared with low-stable, adjusted odds ratios were 2.08 [95% confidence interval (CI) 1.56–2.77] for high-decreasing and 5.81 (95% CI 3.57–9.46) for persistent-high. After centering each trajectory at its initial window, the increasing pattern remained associated with mortality after adjustment for initial lactate and clinical covariates (odds ratio 2.91, 95% CI 1.75–4.85, vs. the largest-decrease pattern). In foldwise cross-validation, the trajectory model had an area under the receiver operating characteristic curve (AUROC) of 0.724 and an area under the precision-recall curve (AUPRC) of 0.572. Last lactate and initial plus last lactate performed better (AUROC 0.734 and 0.736; AUPRC 0.592 and 0.594). Among 429 landmark-eligible eICU-CRD patients assigned to fixed MIMIC-IV centroids, mortality ranged from 25.49% to 78.12%; in 366 complete cases, the adjusted risk ratio for group 4 was 2.30 (95% CI 1.47–3.62).

**Conclusion:**

First-day lactate trajectories provided a clinically interpretable framework for characterizing lactate burden and evolution after repeated testing. The low-stable and persistent-high extremes showed the most consistent cross-database separation, whereas the four-group partition should be interpreted as a descriptive continuum rather than four validated clinical risk classes. Simpler last-lactate summaries provided better discrimination when prediction alone was the objective.

## Introduction

Cardiogenic shock is a life-threatening syndrome characterized by circulatory failure and inadequate tissue perfusion. Although substantial progress has been made in coronary revascularization, vasoactive therapy, mechanical ventilation, renal replacement therapy, and the use of mechanical circulatory support in selected patients, short-term mortality remains unacceptably high (1, 2). In this context, early and reliable risk stratification is clinically important, particularly for distinguishing patients whose tissue perfusion improves after initial treatment from those who continue to deteriorate despite intensive resuscitation.

Blood lactate is routinely measured in patients with cardiogenic shock and provides an accessible marker of the severity and persistence of systemic hypoperfusion. However, lactate elevation is not determined by oxygen delivery alone; it also reflects adrenergic activation, altered cellular metabolism, and impaired hepatic or renal clearance. Previous studies have shown that both lactate concentration and lactate clearance are associated with prognosis in cardiogenic shock (3, 4). Lactate clearance has also been investigated as a resuscitation target in sepsis (5), while serum lactate has been associated with mortality independently of shock severity and organ failure (6). Nevertheless, commonly used measures, such as the initial lactate value or percentage lactate clearance, reduce a complex and evolving physiological process to one or two summary measurements. As a result, they may fail to differentiate a patient with a moderate early lactate elevation followed by rapid recovery from a patient with persistent hyperlactatemia throughout the first day of intensive care.

Evaluating lactate as a first-day trajectory may therefore provide a more clinically meaningful representation of the patient's response to treatment. This approach, however, introduces an important methodological challenge: a complete 24-hour trajectory can only be observed in patients who survive long enough to reach the end of the measurement period. Prognostic analyses must therefore account for this landmark to avoid attributing early deaths to trajectory patterns that could never have been fully observed. In addition, trajectory patterns identified in a single database may not transport directly to other settings, where patient populations, institutional practices, laboratory sampling, and documentation systems differ.

To address these issues, we used MIMIC-IV for trajectory discovery and model development and eICU-CRD for an external transportability assessment. Our primary aim was not to establish a prediction model superior to simple lactate summaries, but to determine whether first-day lactate trajectories could provide a reproducible and clinically interpretable framework for dynamic risk characterization among patients who survived to a prespecified 24-hour landmark. We also assessed whether the mortality contrast associated with these patterns could be transported to an independent multicenter ICU database. Predictive performance was evaluated as a secondary comparison with simpler lactate measures.

## Materials and methods

### Study design, data sources, and population

This retrospective multicohort study used two credentialed-access critical care databases. MIMIC-IV version 3.1 contains admissions to Beth Israel Deaconess Medical Center from 2008 to 2022 and was used as the discovery and model-development cohort (7). eICU-CRD contains multicenter ICU admissions from 2014 to 2015 and was used to assess external transportability (8). This design tested whether trajectory profiles and their associated mortality contrasts from a more recent single-center cohort persisted in an earlier multicenter cohort. Both databases contain de-identified diagnoses, laboratory measurements, vital signs, treatments, severity scores, and hospital outcomes. We did not use these data to assess treatment effectiveness.

The target population for trajectory analyses was adult ICU patients with cardiogenic shock who underwent repeated lactate testing during the first ICU day. The exposure was the completed 24-hour lactate pattern. Primary association and prediction analyses used a 24-hour landmark, with subsequent in-hospital mortality as the outcome among patients alive and still hospitalized at that time.

The study was reported according to STROBE and RECORD guidance for observational studies using routinely collected health data (9, 10). Regression and machine learning prediction analyses were reported according to TRIPOD + AI guidance (11).

In MIMIC-IV, cardiogenic shock was identified using ICD-9 code 78551 or ICD-10 code R570. Only the first ICU stay was included for patients with multiple stays. The primary trajectory analysis required at least two lactate measurements within the first 24 h after ICU admission.

In eICU-CRD, cardiogenic shock was identified from the diagnosis table using the diagnosis term “cardiogenic shock” or ICD-9 code 785.51. Only the first ICU stay per patient was included, with the same requirement for repeated lactate measurements.

### Lactate measurements

In MIMIC-IV, lactate values were extracted from mimiciv_hosp.labevents using lactate item IDs 50813, 52442, and 53154. Lactate dehydrogenase measurements were excluded. In eICU-CRD, lactate values were extracted from the lab table using labname = “lactate”. Specimen source was not consistently harmonized across the databases, so arterial and venous measurements were not separated. Only measurements timestamped from ICU admission through 24 h were used; emergency-department measurements obtained before ICU admission were excluded. Values were analyzed in mmol/L, and no unit conversion was required in the extracted records.

Lactate values were restricted to positive values no greater than 50 mmol/L to exclude implausible or miscoded results; the maximum retained values were 27.0 mmol/L in MIMIC-IV and 26.0 mmol/L in eICU-CRD. For each ICU stay, all lactate measurements from ICU admission to 24 h were extracted. Summary features included initial, last, peak, minimum, and mean observed lactate; lactate slope and clearance; the median observed value at 18–24 h; a time-weighted mean; any value of at least 4 mmol/L; and persistent hyperlactatemia. The time-weighted mean used trapezoidal integration over 0–24 h, with the nearest observed value extended to the interval boundaries. Lactate clearance was calculated as (initial lactate − last lactate)/initial lactate * 100. Persistent hyperlactatemia was defined as a last lactate within 24 h of at least 4 mmol/L.

### Lactate trajectory modeling

The first 24 h after ICU admission were divided into four intervals: 0–6, 6–12, 12–18, and 18–24 h. Measurements sharing an identical timestamp were collapsed to their median before window construction, and the median lactate value within each interval was then calculated. Missing interval values were filled using within-patient interpolation for internal gaps and nearest observed within-patient values for edge windows. Cohort median filling was reserved only for any remaining window-level missingness after within-patient filling. The proportion of observed, interpolated, and edge-filled lactate windows was reported overall and by trajectory group. We also summarized the interval between the final measured lactate and the 24-hour landmark.

Lactate values were transformed using log1p and standardized before clustering. *K*-means solutions from *K* = 2 to K = 5 were fitted using random_state = 2026 and n_init = 50. We compared silhouette, Calinski-Harabasz, and Davies-Bouldin indices, cluster sizes, centroid separation, and the temporal shape of the mean profiles. Mortality was not used to select *K*. The *K* = 2 solution had the strongest internal separation but combined decreasing and persistent patterns that were clinically distinct in the observed profiles. *K* = 4 was not prespecified as a clinical classification or anchored to a predefined lactate-threshold taxonomy; it was retained before outcome modeling as a pragmatic analytical resolution to distinguish low lactate burden, intermediate decreasing patterns, high decreasing patterns, and persistent hyperlactatemia. The 4 mmol/L threshold was used only for the separate persistent-hyperlactatemia sensitivity analysis and was not used as a clustering criterion. *K* = 4 was therefore treated as a pragmatic compromise between statistical cohesion, stability, and temporal resolution rather than a uniquely optimal partition. Clusters were ordered by mean 24-hour lactate burden and final 18–24-hour lactate. Assignment clarity was examined using the nearest and second-nearest centroid distances, their absolute and relative margins, and pairwise centroid distances in standardized log-lactate space.

Sensitivity analyses used 2, 3, and 5 groups and repeated the 4-group model among patients with at least three lactate measurements, at least three directly observed time windows, or measurements in all four time windows. We also required at least three directly observed windows with a measured 18–24-hour value, and performed a no-edge-filling analysis requiring measured first and final windows. To separate temporal shape from absolute lactate burden, we subtracted each patient's log-transformed 0–6-hour value from the three later log-transformed windows and fitted an exploratory 4-group K-means model to these centered changes. The centered groups were ordered from the largest decrease to the largest increase; their mortality associations were adjusted for the primary covariates and log-transformed initial lactate. Cluster stability was assessed across 100 random seeds and 100 patient-level bootstrap samples using the adjusted Rand index. The primary association analysis was restricted to patients alive and still hospitalized 24 h after ICU admission; *K*-means was also refitted within this landmark risk set to assess whether early deaths influenced the cluster solution. Full-cohort associations and an analysis excluding ICU stays shorter than 24 h were treated as sensitivity analyses. Subgroup analyses compared acute myocardial infarction-related cardiogenic shock (AMI-CS) with non-AMI-CS within the landmark risk set. AMI-CS was defined using acute myocardial infarction codes from the same hospital admission (ICD-9 410.xx, excluding subsequent episode codes ending in 2, or ICD-10 I21/I22).

### Covariates and outcome

MIMIC-IV covariates were extracted from mimiciv_derived tables where available. Covariates included age, sex, SOFA score (12), SAPS II, OASIS, Charlson comorbidity index (13) and selected comorbidities, first-day vital signs, first-day laboratory tests, mechanical ventilation within 24 h, and vasoactive agent use within 24 h.

The primary adjusted models included age, SOFA score, Charlson comorbidity index, mean arterial pressure, creatinine, mechanical ventilation, and vasoactive agent use. These models were intended for descriptive risk stratification after completion of the first ICU day, not estimation of an independent biological or treatment effect of trajectory membership. Adjusted association models used complete-case analysis for the covariates included in each model, and model-specific complete-case sample sizes were reported. Because SOFA, mean arterial pressure, creatinine, mechanical ventilation, and vasoactive agent use overlapped temporally with trajectory formation, sensitivity models excluded treatment variables and used a reduced severity specification. In eICU-CRD, sensitivity models for both fixed-centroid and re-clustered assignments retained all 429 landmark patients by median-imputing missing APACHE IVa score, mean arterial pressure, creatinine, and ventilation status and including indicators for the two observed missingness patterns.

In eICU-CRD, covariates included age, APACHE IVa score (14), acute physiology score, mean arterial pressure, creatinine, mechanical ventilation, and vasoactive agent use. The SQL extraction explicitly selected records with apacheversion = “IVa”. Because acute physiology score is a component of the APACHE framework, the primary eICU-CRD model included APACHE IVa score but not acute physiology score. Sensitivity models substituted acute physiology score for APACHE IVa score or included both scores to assess whether the results depended on severity-score specification.

The primary outcome was in-hospital mortality. In MIMIC-IV, in-hospital mortality was defined using hospital_expire_flag. In eICU-CRD, in-hospital mortality was defined using hospitaldischargestatus = “Expired”. For descriptive timing analyses, a death within 24 h required a recorded death time more than 0 and no more than 24 h after ICU admission. Records with a death time before ICU admission were excluded from this timing summary but retained for hospital-discharge-based landmark classification.

### Statistical analysis

First-day clinical characteristics were summarized by lactate trajectory group. Continuous variables were reported as median and interquartile range. Categorical variables were reported as count and percentage. To assess selection into the analytic cohort, patients with at least two early lactate measurements were compared with those having fewer measurements using available age, sex, mortality, and 24-hour disposition data; standardized mean differences were reported. Severity measures were not available from the current extraction for patients outside the trajectory cohorts.

The association between lactate trajectory group and in-hospital mortality was examined using logistic regression. The low-stable trajectory group was used as the reference group. Alongside adjusted odds ratios, we reported covariate-standardized mortality probabilities, adjusted risk differences per 100 patients, and adjusted risk ratios from modified Poisson models with robust standard errors. Confidence intervals for standardized probabilities and risk differences were obtained from 5,000 draws from the fitted logistic-model coefficient distribution. For eICU-CRD, standard errors were clustered by hospital. Variance inflation factors were calculated for the primary adjusted model to assess linear multicollinearity, while recognizing that low variance inflation does not remove conceptual or temporal overlap among covariates.

In MIMIC-IV, the primary adjusted model included age, SOFA score, Charlson comorbidity index, mean arterial pressure, creatinine, mechanical ventilation, and vasoactive agent use. In eICU-CRD, the primary transportability model adjusted for age, APACHE IVa score, mean arterial pressure, creatinine, mechanical ventilation, and vasoactive agent use. All statistical tests were two-sided, with an alpha level of 0.05. The primary association analysis estimated the three trajectory-group contrasts against the low-stable reference group in the MIMIC-IV landmark risk set; the corresponding eICU-CRD contrasts evaluated external transportability. We did not adjust these three primary contrasts for multiple testing; *P* values and 95% confidence intervals are reported. Alternative cluster solutions, observation-window analyses, subgroup analyses, treatment-variable specifications, and algorithm comparisons were exploratory; we interpreted them by effect size, precision, and consistency rather than statistical significance alone.

### Prediction modeling

Prediction models were evaluated at a 24-hour landmark in MIMIC-IV (15). The risk set comprised patients alive and still hospitalized 24 h after ICU admission, defined by hospital discharge more than 24 h after ICU entry; the outcome was subsequent in-hospital mortality. Recorded death times were summarized separately and were not used to define continued hospitalization. We used 5-fold stratified cross-validation. Numeric imputation and scaling were fitted within each training fold and applied to the corresponding validation fold. For every model containing trajectory group, lactate-window filling parameters, scaling, K-means centroids, trajectory assignment, and model fitting were repeated within each training fold before application to the corresponding validation fold. This foldwise pipeline was the primary prediction analysis. Features and model settings were specified before comparison; no data-driven feature selection or hyperparameter optimization was performed. A sensitivity analysis assigned trajectory labels once from the full trajectory-analysis cohort to quantify the effect of global unsupervised preprocessing. We compared a clinical base model with models adding initial, last, initial and last, 18–24-hour, mean observed, time-weighted mean, peak, or minimum lactate; lactate slope; initial lactate plus clearance; persistent hyperlactatemia; trajectory group; or the full set of lactate dynamic features.

Performance measures were AUROC, AUPRC, Brier score, calibration intercept, and calibration slope. Calibration intercept and slope were estimated from a logistic calibration model with the logit of the out-of-fold predicted probability as the predictor. Patient-level bootstrap resampling with 500 iterations provided 95% confidence intervals for changes in AUROC and AUPRC (16). These bootstrap intervals resampled the out-of-fold predictions and therefore quantified sampling uncertainty conditional on the fitted cross-validation pipeline; they did not refit the complete pipeline within each bootstrap sample. To quantify split and preprocessing variability, the complete 5-fold pipeline was repeated 20 times with different split seeds, with all preprocessing, *K*-means fitting, trajectory assignment, and model fitting repeated within each training fold. Decision curve analysis (17) and calibration plots (18) were generated for the clinical base, trajectory, and full lactate dynamic models, and split-to-split variation in net benefit was summarized. Five predefined algorithms were compared with fixed settings: L2-penalized logistic regression, random forest, ExtraTrees, gradient boosting, and histogram gradient boosting. These fixed-setting comparisons were descriptive. The models were not deployed or recalibrated in eICU-CRD because the two databases did not provide a fully harmonized predictor set.

### External transportability assessment

The eICU-CRD cohort was used to evaluate external transportability. In the primary analysis, MIMIC-IV-derived centroids, standardization parameters, and ordering rules were applied directly without re-clustering. Patients were assigned to the nearest MIMIC-IV centroid by squared Euclidean distance after log1p transformation and MIMIC-IV-based standardization. Re-clustering eICU-CRD with the same four-window procedure was treated as a sensitivity analysis. We reported nearest-centroid distances, assignment margins, the proportion above the 95th percentile of the MIMIC-IV nearest-distance distribution, and agreement between fixed-centroid assignment and eICU re-clustering. Primary mortality associations were examined among patients alive and still hospitalized at the 24-hour landmark, defined by hospitaldischargeoffset >1,440 min, using eICU-available covariates and APACHE IVa score; standard errors were clustered by hospital. Modified Poisson regression provided adjusted risk ratios, while logistic regression provided odds ratios. Firth-penalized logistic regression was used as an unclustered small-sample sensitivity analysis for possible separation (19). Full-cohort associations and eICU re-clustering were retained as sensitivity analyses. Because several MIMIC-IV predictors could not be reconstructed identically in eICU-CRD, this assessment addressed trajectory profiles and their mortality contrasts, not the complete MIMIC-IV prediction model.

### Ethics and data availability

MIMIC-IV and eICU-CRD contain de-identified data and are available through PhysioNet to credentialed users who complete the required human-subjects training and data use agreements. The source database projects obtained the required institutional approvals or waivers for release of de-identified data. This secondary analysis involved no direct patient contact, used no identifiable information, and required no additional informed consent.

Data were extracted using PostgreSQL. We conducted statistical analyses and developed machine learning models in Python 3.14.5 using pandas 3.0.4, statsmodels 0.14.6, scikit-learn 1.9.0, SciPy 1.18.0, and Matplotlib 3.11.0. The SQL and Python scripts are available from the public GitHub repository at https://github.com/shupengqin/early-lactate-trajectories-cardiogenic-shock; the version corresponding to this manuscript is preserved at commit 8beb7404580540b206a809e84ad3717c8dd8f3b8 (https://github.com/shupengqin/early-lactate-trajectories-cardiogenic-shock/tree/8beb7404580540b206a809e84ad3717c8dd8f3b8).

## Results

### Cohort selection

**Figure 1:**
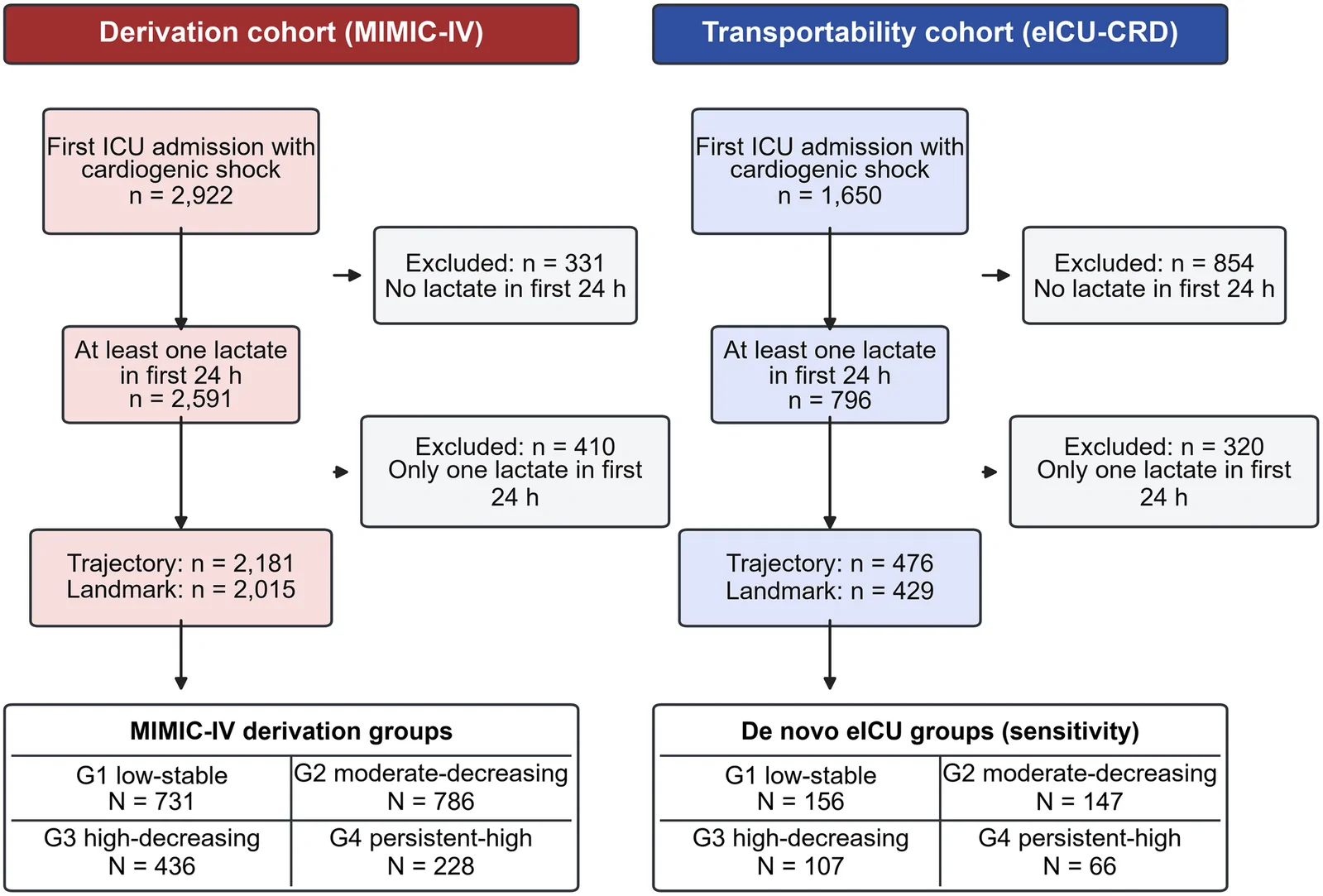
Study cohort construction, trajectory eligibility, and 24-hour landmark risk sets. The flow diagram shows construction of the MIMIC-IV derivation cohort and eICU-CRD transportability cohort from adult first ICU admissions with cardiogenic shock. Exclusion boxes show patients without lactate measurement within the first 24 h and patients with only one early lactate measurement, who were not eligible for trajectory modeling. The final cohort boxes report the number alive and still hospitalized at 24 h. The final row reports the full MIMIC-IV derivation-group distribution and the *de novo* eICU-CRD re-clustered group distribution used in sensitivity analysis; the primary fixed-centroid landmark assignment is shown in Figures 2E,F and Table 5.

In MIMIC-IV, 2,922 first ICU admissions met the strict ICD-based definition of cardiogenic shock. Among them, 1,114 patients died during hospitalization, corresponding to an in-hospital mortality of 38.12%. Lactate was measured at least once within the first 24 h in 2,591 patients and at least twice in 2,181 patients. Recorded deaths within 24 h occurred in 27 of 331 patients without an early lactate measurement, 46 of 410 patients with one early lactate measurement, and 157 of 2,181 patients with at least two early lactate measurements (Supplementary Table S15). Two hospital deaths had recorded death times before ICU admission and were excluded from this timing summary. Patients with repeated testing were younger and less often female than those with fewer than two measurements; in-hospital mortality was 38.70% vs. 36.44% (Supplementary Table S23). Trajectories were constructed for 2,181 patients with repeated early lactate measurements. Hospital discharge records showed that 166 were no longer hospitalized at 24 h: 164 had an expired hospital admission and 2 were discharged alive. For 6 of the expired admissions, recorded death time was later than 24 h despite hospital discharge within 24 h. The landmark risk set therefore included 2,015 patients, of whom 680 subsequently died in hospital (Figure 1; Supplementary Table S15).

In eICU-CRD, 1,650 first ICU admissions met the cardiogenic shock definition. In-hospital mortality was 36.12% (596 deaths). A total of 476 patients had at least two lactate measurements within the first 24 h; their mortality was 47.48%, compared with 31.52% among the 1,174 patients with fewer measurements (Supplementary Table S23). The landmark risk set included 429 patients, of whom 186 subsequently died in hospital (Figure 1).

### Early lactate trajectories in MIMIC-IV

**Figure 2:**
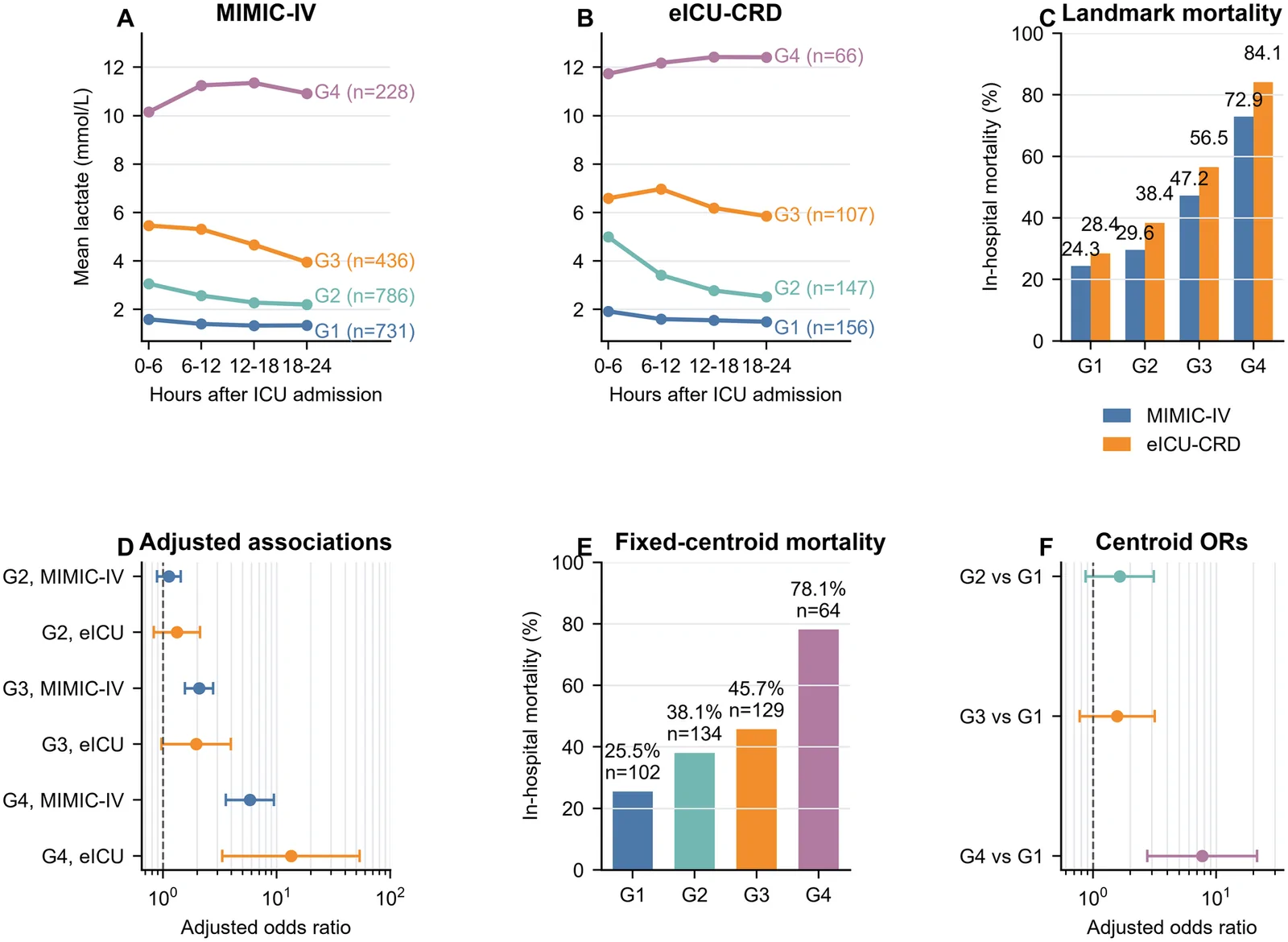
Lactate trajectory patterns, eICU re-clustering sensitivity, and fixed-centroid transportability. **(Panel A)** shows the four MIMIC-IV trajectory patterns. **(Panels B–D)** show the independently re-clustered eICU-CRD sensitivity analysis, including mean profiles, landmark mortality, and adjusted odds ratios. **(Panels E,F)** show the primary external transportability results after direct assignment of landmark-eligible eICU-CRD patients to fixed MIMIC-IV-derived centroids.

First-day clinical characteristics across the MIMIC-IV trajectory groups are summarized in Table 1, and missingness for selected variables in MIMIC-IV and eICU-CRD is summarized in Supplementary Table S2. Four early lactate trajectory groups were identified in MIMIC-IV (Figure 2A, Table 2). The low-stable group included 731 patients and showed persistently low lactate levels from 0 to 6 h to 18–24 h (mean 1.59 to 1.34 mmol/L). The moderate-decreasing group included 786 patients and showed a decline from 3.06 to 2.20 mmol/L. The high-decreasing group included 436 patients and showed a decline from 5.46 to 3.95 mmol/L. The persistent-high group included 228 patients and remained markedly elevated throughout the first 24 h (10.15 to 10.92 mmol/L).

**Table 1:** Clinical characteristics in the full MIMIC-IV trajectory cohort by lactate trajectory group.

Characteristic	Overall	Group 1	Group 2	Group 3	Group 4	Max SMD
N	2181	731	786	436	228	
Age, years	69.0 [59.0, 77.0]	67.0 [57.0, 76.5]	69.5 [59.0, 78.0]	69.0 [60.0, 78.0]	68.0 [59.0, 78.0]	0.1
SOFA score	8.0 [5.0, 11.0]	6.0 [4.0, 9.0]	8.0 [5.0, 10.0]	10.0 [7.0, 12.0]	12.0 [9.8, 14.0]	1.49
SAPS II	47.0 [37.0, 59.0]	41.0 [32.5, 50.0]	45.0 [37.0, 55.0]	55.0 [45.0, 65.2]	65.0 [55.0, 73.0]	1.63
OASIS	37.0 [30.0, 44.0]	34.0 [27.0, 40.0]	36.0 [30.0, 42.0]	40.0 [34.0, 46.0]	45.0 [39.0, 50.0]	1.12
Charlson comorbidity index	6.0 [4.0, 8.0]	6.0 [4.0, 8.0]	6.0 [5.0, 8.0]	6.0 [4.0, 8.0]	6.0 [4.0, 8.0]	0.03
Heart rate, mean	87.5 [76.4, 101.3]	86.0 [74.3, 98.6]	86.3 [77.4, 100.3]	90.6 [78.3, 104.3]	91.0 [79.5, 107.2]	0.34
Mean arterial pressure, mean	74.5 [68.9, 80.2]	74.9 [69.9, 80.1]	75.1 [69.5, 81.0]	74.8 [68.4, 80.2]	70.6 [63.2, 76.0]	0.56
Respiratory rate, mean	20.5 [18.0, 23.6]	19.5 [17.2, 22.0]	20.3 [18.1, 23.4]	21.5 [18.8, 24.7]	23.5 [20.9, 26.8]	0.86
SpO2, mean	97.0 [95.4, 98.3]	96.9 [95.5, 98.3]	97.2 [95.8, 98.4]	97.0 [95.2, 98.4]	95.4 [90.8, 97.4]	0.76
Hemoglobin, minimum	9.8 [8.1, 11.6]	9.9 [8.4, 11.5]	10.0 [8.3, 12.0]	9.4 [7.9, 11.6]	8.8 [7.0, 10.7]	0.52
Platelets, minimum	159.0 [111.0, 214.0]	172.0 [133.0, 230.8]	159.0 [115.0, 215.0]	139.0 [92.8, 196.0]	128.0 [67.8, 191.0]	0.63
White blood cells, maximum	15.9 [11.5, 21.4]	13.9 [10.4, 18.5]	15.9 [11.9, 21.1]	17.8 [12.9, 24.0]	19.6 [14.3, 25.6]	0.49
Bicarbonate, minimum	18.0 [15.0, 21.0]	20.0 [18.0, 23.0]	19.0 [16.0, 21.0]	16.0 [12.0, 19.0]	10.0 [8.0, 13.5]	2.08
BUN, maximum	36.0 [23.0, 55.0]	35.0 [23.0, 56.0]	36.0 [22.0, 56.0]	36.0 [24.0, 53.0]	37.5 [28.0, 54.0]	0.04
Creatinine, maximum	1.8 [1.2, 2.8]	1.6 [1.1, 2.6]	1.7 [1.2, 2.7]	1.9 [1.3, 2.8]	2.5 [2.0, 3.5]	0.45
Sodium, minimum	136.0 [132.0, 138.0]	135.0 [132.5, 138.0]	135.0 [132.0, 138.0]	136.0 [132.0, 138.0]	136.0 [132.0, 140.0]	0.21
Potassium, maximum	4.8 [4.4, 5.5]	4.6 [4.3, 5.2]	4.8 [4.4, 5.5]	5.0 [4.4, 5.8]	5.2 [4.8, 6.0]	0.54
Initial lactate	2.6 [1.7, 4.4]	1.5 [1.2, 1.9]	2.8 [2.2, 3.6]	4.8 [3.5, 6.9]	8.9 [6.3, 12.9]	2.29
Last lactate within 24 h	1.9 [1.4, 3.2]	1.3 [1.0, 1.6]	2.0 [1.6, 2.4]	3.4 [2.5, 4.7]	10.6 [7.2, 14.4]	2.76
Peak lactate within 24 h	3.4 [2.2, 6.5]	1.8 [1.5, 2.3]	3.5 [2.8, 4.5]	7.1 [5.8, 8.8]	13.1 [11.0, 16.9]	3.98
Female sex	838 (38.4)	281 (38.4)	285 (36.3)	170 (39.0)	102 (44.7)	0.17
Myocardial infarction	1,049 (48.1)	364 (49.8)	379 (48.2)	197 (45.2)	109 (47.8)	0.09
Congestive heart failure	1,705 (78.2)	621 (85.0)	646 (82.2)	293 (67.2)	145 (63.6)	0.49
Peripheral vascular disease	426 (19.5)	140 (19.2)	166 (21.1)	78 (17.9)	42 (18.4)	0.08
Chronic pulmonary disease	609 (27.9)	244 (33.4)	207 (26.3)	97 (22.2)	61 (26.8)	0.25
Diabetes without complications	589 (27.0)	187 (25.6)	189 (24.0)	140 (32.1)	73 (32.0)	0.18
Diabetes with complications	438 (20.1)	159 (21.8)	153 (19.5)	80 (18.3)	46 (20.2)	0.08
Renal disease	852 (39.1)	290 (39.7)	322 (41.0)	155 (35.6)	85 (37.3)	0.11
Malignant cancer	174 (8.0)	52 (7.1)	53 (6.7)	44 (10.1)	25 (11.0)	0.15
Mechanical ventilation within 24 h	1,387 (63.6)	402 (55.0)	479 (60.9)	329 (75.5)	177 (77.6)	0.48
Vasoactive agent within 24 h	1,471 (67.4)	448 (61.3)	507 (64.5)	338 (77.5)	178 (78.1)	0.37
In-hospital mortality	844 (38.7)	184 (25.2)	240 (30.5)	225 (51.6)	195 (85.5)	1.21

Values are median [interquartile range] or *n* (%), unless otherwise indicated. Max SMD denotes the maximum absolute standardized mean difference across pairwise group comparisons. Group 1, low-stable; Group 2, moderate-decreasing; Group 3, high-decreasing; Group 4, persistent-high. SOFA, Sequential Organ Failure Assessment; SAPS II, Simplified Acute Physiology Score II; OASIS, Oxford Acute Severity of Illness Score; SpO2, peripheral oxygen saturation; SMD, standardized mean difference.

**Table 2:** Lactate trajectory groups in MIMIC-IV and the independently re-clustered eICU-CRD sensitivity analysis.

Cohort	Group	*N*	Mean lactate 0–6 h	Mean lactate 6–12 h	Mean lactate 12–18 h	Mean lactate 18–24 h	Deaths	Mortality, %
MIMIC-IV	Group 1	731	1.59	1.4	1.33	1.34	184	25.17
MIMIC-IV	Group 2	786	3.06	2.57	2.28	2.2	240	30.53
MIMIC-IV	Group 3	436	5.46	5.31	4.67	3.95	225	51.61
MIMIC-IV	Group 4	228	10.15	11.25	11.35	10.92	195	85.53
eICU-CRD	Group 1	156	1.92	1.6	1.55	1.49	44	28.21
eICU-CRD	Group 2	147	4.99	3.42	2.78	2.52	58	39.46
eICU-CRD	Group 3	107	6.59	6.97	6.19	5.85	65	60.75
eICU-CRD	Group 4	66	11.73	12.18	12.42	12.41	59	89.39

Mean lactate values are shown for four 6-hour windows during the first 24 h after ICU admission. Group sizes and mortality are descriptive values from the full trajectory-eligible cohorts. The eICU-CRD rows are from an independent re-clustering sensitivity analysis; fixed-centroid transportability is reported in Table 5. Mortality denotes in-hospital mortality. Group 1, low-stable; Group 2, moderate-decreasing; Group 3, high-decreasing; Group 4, persistent-high. eICU-CRD, eICU Collaborative Research Database.

The lactate-window audit showed that observed measurements were available for 91.47% of 0–6-hour windows, 77.81% of 6–12-hour windows, 68.78% of 12–18-hour windows, and 61.71% of 18–24-hour windows (Supplementary Table S16). No cohort-median filling was required after within-patient interpolation and nearest-value edge filling. In the persistent-high group, 120 of 228 final 18–24-hour windows (52.63%) were directly observed and 108 (47.37%) were edge-filled (Supplementary Table S24). The median interval from the last measured lactate to the landmark was 4.28 h overall and 5.71 h in the persistent-high group; 32.02% of the persistent-high group had their last measurement more than 12 h before the landmark. Ten records represented five duplicate timestamps, four with discordant values; collapsing these timestamps to their median did not change any MIMIC-IV trajectory assignments. Among patients eligible for trajectory analysis, those with at least five lactate measurements had higher illness severity than those with two measurements (median SOFA 9 vs. 7) and higher mortality (41.46% vs. 36.51%; Supplementary Tables S15, S16).

In the full trajectory-eligible MIMIC-IV cohort, descriptive in-hospital mortality increased from 25.17% in the low-stable group to 85.53% in the persistent-high group (Table 2). These full-cohort descriptive values were not used for the primary association analysis.

### Association with in-hospital mortality

At the 24-hour landmark, subsequent mortality was 24.34% in the low-stable group, 29.59% in the moderate-decreasing group, 47.25% in the high-decreasing group, and 72.88% in the persistent-high group (Figure 2C). Compared with the low-stable group, adjusted odds ratios were 1.13 (95% CI 0.89–1.44; *P* = 0.319) for the moderate-decreasing group, 2.08 (95% CI 1.56–2.77; *P* < 0.001) for the high-decreasing group, and 5.81 (95% CI 3.57–9.46; *P* < 0.001) for the persistent-high group (Figure 2D). Covariate-standardized mortality was 27.58% in the low-stable group and 64.85% in the persistent-high group, an adjusted difference of 37.26 deaths per 100 patients (95% CI 26.72–46.40); the corresponding adjusted risk ratio was 2.15 (95% CI 1.77–2.61; Table 3). The adjusted model included 2,013 of 2,015 patients.

**Table 3:** Adjusted mortality associations and standardized estimates in the MIMIC-IV 24-hour landmark risk set.

Group	*N* at landmark	Deaths	Model included, n	Excluded for missing covariates, n	Adjusted mortality, % (95% CI)	Risk difference per 100 (95% CI)	Adjusted RR (95% CI)	Adjusted OR (95% CI)	*P* value
Group 1	723	176	721	2	27.6 (24.4–31.1)	Reference	Reference	Reference	
Group 2	774	229	774	0	29.9 (26.9–33.1)	2.3 (−2.2–6.8)	1.10 (0.94–1.30)	1.13 (0.89–1.44)	0.319
Group 3	400	189	400	0	42.4 (37.8–47.2)	14.8 (8.7–20.6)	1.56 (1.31–1.85)	2.08 (1.56–2.77)	<0.001
Group 4	118	86	118	0	64.8 (55.3–73.3)	37.3 (26.7–46.4)	2.15 (1.77–2.61)	5.81 (3.57–9.46)	<0.001

The low-stable trajectory group was the reference group. Models adjusted for age, SOFA score, Charlson comorbidity index, mean arterial pressure, creatinine, mechanical ventilation, and vasoactive agent use. Adjusted mortality is the marginal probability standardized to the covariate distribution of the complete-case model sample; risk differences compare each group with Group 1. Risk ratios were estimated using modified Poisson regression with robust standard errors, and odds ratios using logistic regression. *P* values correspond to the adjusted odds ratios and are two-sided. RR, risk ratio; OR, odds ratio; CI, confidence interval; SOFA, Sequential Organ Failure Assessment.

Using a simpler binary marker in the MIMIC-IV landmark risk set, persistent hyperlactatemia was also associated with subsequent in-hospital mortality after adjustment (OR 3.19, 95% CI 2.36–4.31; *P* < 0.001; Supplementary Table S14).

### Prediction model performance

**Figure 3:**
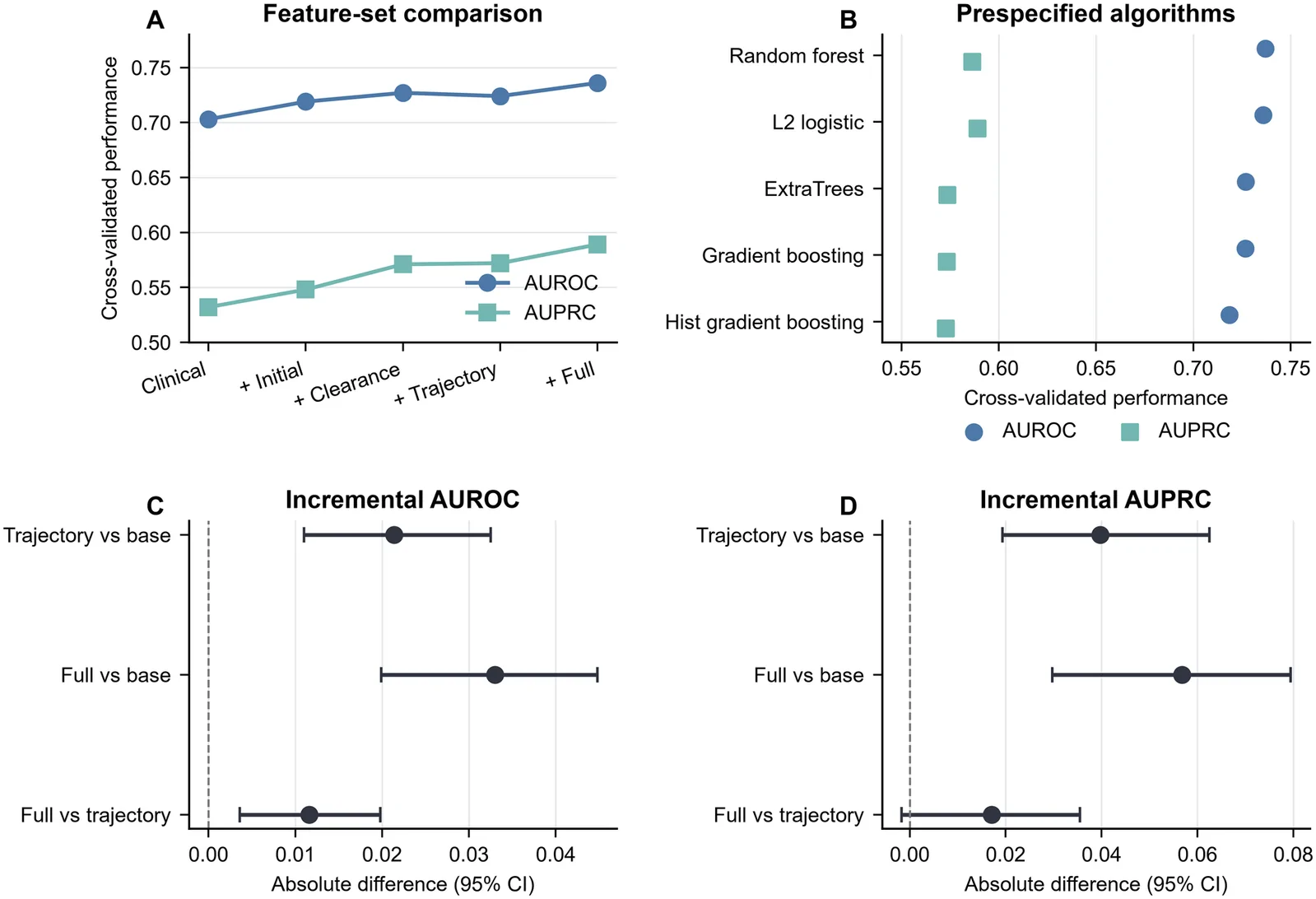
Prediction and machine learning performance at the 24-hour landmark. **(Panel A)** compares AUROC and AUPRC across nested feature sets among patients alive and still hospitalized at 24 h. **(Panel B)** compares five predefined algorithms using the full lactate dynamic feature set. **(Panels C,D)** show bootstrap 95% confidence intervals for incremental AUROC and AUPRC.

**Figure 4:**
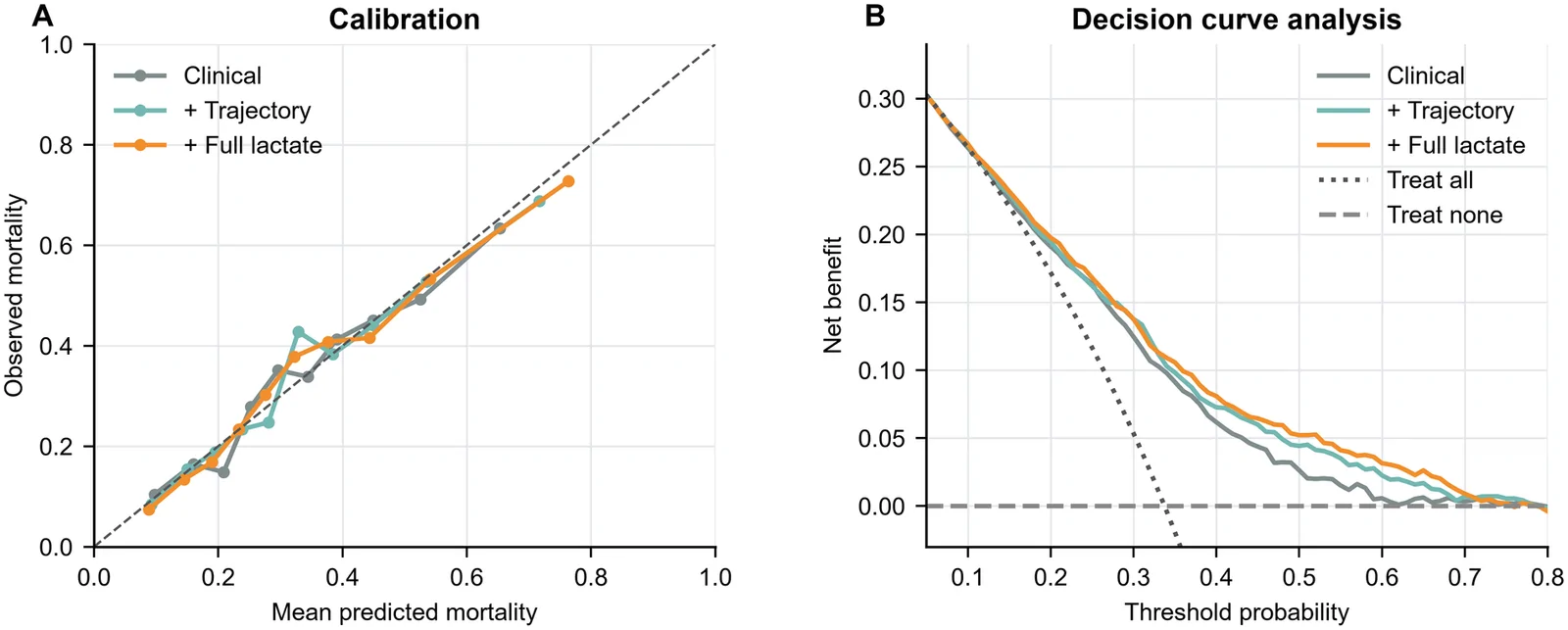
Calibration and decision curve analysis at the 24-hour landmark. **(Panel A)** shows calibration curves for the clinical base, trajectory, and full lactate dynamic models. **(Panel B)** shows decision curves for the same models across threshold probabilities.

The 24-hour landmark risk set included 2,015 patients, of whom 680 subsequently died in hospital. In 5-fold cross-validation with foldwise trajectory fitting, the clinical base model had an AUROC of 0.703 and an AUPRC of 0.532. The corresponding values were 0.719 and 0.548 after adding initial lactate, 0.727 and 0.571 after adding initial lactate plus clearance, and 0.724 and 0.572 after adding trajectory group. The full lactate dynamic model had an AUROC of 0.736 and an AUPRC of 0.589 (Table 4, Figure 3A). Relative to the clinical base model, trajectory group increased AUROC by 0.0214 (95% CI 0.0110–0.0325) and AUPRC by 0.0397 (95% CI 0.0193–0.0625). The corresponding increments for the full lactate dynamic model were 0.0330 (95% CI 0.0199–0.0448) and 0.0568 (95% CI 0.0297–0.0794; Figures 3C,D; Supplementary Table S6). Calibration intercepts were −0.031, −0.021, and −0.049 for the clinical, trajectory, and full lactate models, respectively; the corresponding slopes were 0.950, 0.963, and 0.926 (Table 4). Calibration plots and decision curve analyses are shown in Figures 4A,B, with source data in Supplementary Table S8.

**Table 4:** Prediction model performance in MIMIC-IV.

Model	AUROC	AUPRC	Brier score	Calibration intercept	Calibration slope	Delta AUROC vs base	Delta AUPRC vs base
Clinical base model	0.703	0.532	0.199	−0.031	0.950	0.000	0.000
Clinical base + initial lactate	0.719	0.548	0.195	−0.031	0.949	0.016	0.016
Clinical base + initial lactate + clearance	0.727	0.571	0.192	−0.032	0.948	0.024	0.039
Clinical base + trajectory group	0.724	0.572	0.192	−0.021	0.963	0.021	0.040
Clinical base + full lactate dynamics	0.736	0.589	0.189	−0.049	0.926	0.033	0.057

Performance was estimated using 5-fold stratified cross-validation among MIMIC-IV patients alive and still hospitalized at the 24-hour landmark. For every model containing trajectory group, lactate-window filling, scaling, K-means fitting, trajectory assignment, and prediction-model fitting were repeated within each training fold. Calibration intercept and slope were estimated from a logistic calibration model using the logit of each out-of-fold predicted probability. Ideal calibration corresponds to an intercept of 0 and a slope of 1. Delta values are absolute differences compared with the clinical base model. The analyses quantify incremental prognostic information rather than a ready-to-deploy bedside calculator. AUROC, area under the receiver operating characteristic curve; AUPRC, area under the precision-recall curve.

When trajectory labels were instead assigned once from the full trajectory-analysis cohort, the trajectory model had an AUROC of 0.727 and an AUPRC of 0.573; the full lactate dynamic model had an AUROC of 0.737 and an AUPRC of 0.589 (Supplementary Table S18). These estimates were close to those from the primary foldwise pipeline.

Direct comparison with simpler summaries showed that last lactate alone and initial plus last lactate performed better than trajectory group: AUROC was 0.734 and 0.736, respectively, and AUPRC was 0.592 and 0.594, compared with 0.724 and 0.572 for trajectory group. Relative to last lactate, trajectory group had a lower AUROC by 0.0092 (95% CI −0.0170 to −0.0024) and a lower AUPRC by 0.0200 (95% CI −0.0378 to −0.0016). The corresponding differences vs. initial plus last lactate were −0.0120 (95% CI −0.0192 to −0.0056) and −0.0222 (95% CI −0.0385 to −0.0059). Mean observed, time-weighted mean, and 18–24-hour lactate also performed at least comparably to trajectory group, whereas lactate slope performed worse (Supplementary Tables S10, S11). Across thresholds of 0.30–0.50, trajectory grouping did not consistently improve net benefit over last lactate, initial plus last lactate, or initial lactate plus clearance. Across 20 repeated 5-fold runs of the originally specified models, mean AUROC was 0.725 for trajectory group and 0.729 for initial lactate plus clearance; mean AUPRC was 0.571 for both. Brier scores and calibration slopes were stable across splits (Supplementary Tables S28, S29).

With foldwise trajectory fitting and predefined model settings, AUROC ranged from 0.719 to 0.737 across algorithms. Random forest (0.737) and L2-penalized logistic regression (0.736) performed similarly, followed by ExtraTrees (0.727), gradient boosting (0.727), and histogram gradient boosting (0.719; Figure 3B; Supplementary Table S1). These fixed-parameter comparisons were descriptive.

### Sensitivity analyses

**Figure 5:**
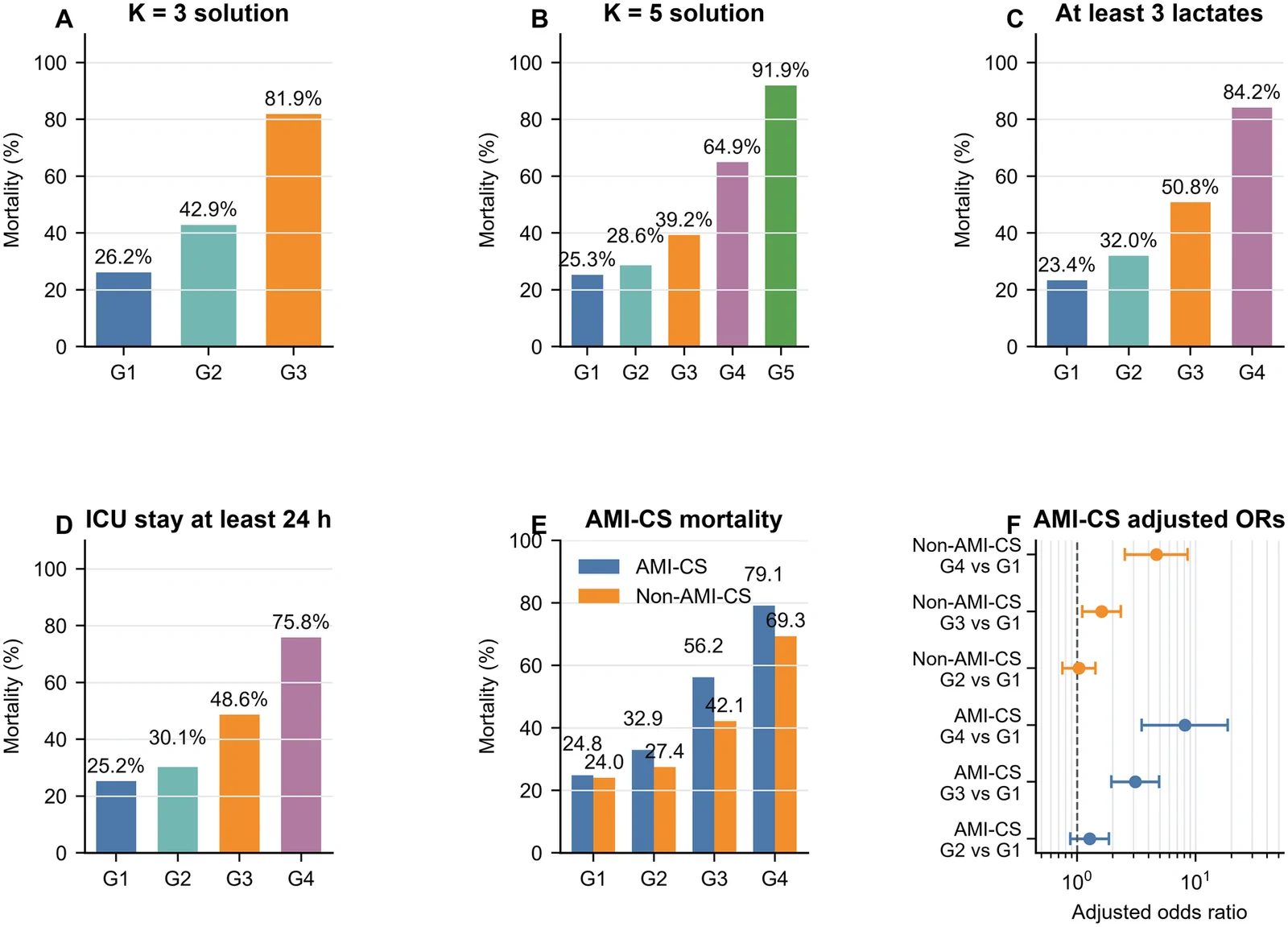
Robustness and subgroup analyses. **(Panels A,B)** show descriptive full-cohort mortality gradients in *K* = 3 and *K* = 5 trajectory sensitivity analyses. **(Panel C)** shows the descriptive full-cohort analysis restricted to patients with at least three lactate measurements within the first 24 h. **(Panel D)** shows the analysis excluding ICU stays shorter than 24 h. **Panels E and F** show mortality gradients and adjusted odds ratios in AMI-CS and non-AMI-CS subgroups within the 24-hour landmark risk set.

The mortality gradient persisted across the sensitivity analyses (Supplementary Table S3). In the full trajectory-eligible MIMIC-IV cohort, the adjusted OR for the persistent-high group was 11.61 (95% CI 7.46–18.07); the corresponding estimate in the eICU re-clustered full-cohort sensitivity analysis was 20.99 (95% CI 5.40–81.63; Supplementary Table S22). The maximum variance inflation factor in the full-cohort MIMIC-IV model was 1.93, although this does not exclude conceptual overlap between severity scores and treatment variables (Supplementary Table S9). In the 2-group solution, mortality was 28.40% and 66.72%, and the higher-lactate group had an adjusted OR of 3.78 (95% CI 3.00–4.77). In the 3-group solution, mortality increased from 26.16% to 81.88%, with an adjusted OR of 8.92 (95% CI 6.17–12.91) for the highest group (Figure 5A).

In the 5-group model, mortality increased from 25.27% to 91.89%; the adjusted OR for the highest group was 21.15 (95% CI 11.02–40.59; Figure 5B; Supplementary Table S3). The 4-group results were also similar among patients with at least three lactate measurements (*n* = 1,814; Figure 5C) or at least three observed windows (*n* = 1,484); adjusted ORs for the highest group were 11.45 (95% CI 7.20–18.22) and 8.72 (95% CI 5.33–14.26), respectively (Supplementary Table S3). Requiring at least three observed windows and an observed 18–24-hour value yielded 1,165 landmark patients; mortality ranged from 24.68% to 75.00%, and the adjusted OR for the highest pattern was 6.86 (95% CI 3.97–11.87). The no-edge-filling analysis included 1,110 landmark patients with measured first and final windows; mortality ranged from 24.52% to 76.19%, and the corresponding OR was 7.36 (95% CI 4.19–12.92; Supplementary Table S3). Among 835 patients with all four windows directly observed, the landmark analysis included 818 patients and required no filling; mortality ranged from 25.00% to 78.79%, and the adjusted OR for the highest pattern was 9.46 (95% CI 4.97–17.98; Supplementary Table S26).

All three internal cluster indices favored *K* = 2 over *K* = 4: silhouette scores were 0.548 and 0.344, Calinski-Harabasz indices were 3,174.9 and 2,687.9, and Davies-Bouldin indices were 0.720 and 0.955, respectively. *K* = 4 was therefore retained for temporal resolution rather than statistical optimality. For the *K* = 4 solution, adjusted Rand indices ranged from 0.968 to 1.000 across random seeds and from 0.729 to 0.988 across patient bootstrap samples (median 0.940). Pairwise centroid distances ranged from 1.36 to 5.73 in standardized log-lactate space; the median relative assignment margin was 0.52 (Supplementary Tables S17, S25). Refitting *K* = 4 within the landmark risk set yielded an adjusted Rand index of 0.873 relative to the full-cohort labels; the persistent-high pattern remained associated with subsequent mortality (OR 5.02, 95% CI 3.26–7.73; Supplementary Table S17). After excluding ICU stays shorter than 24 h, group 4 remained associated with mortality (OR 6.21, 95% CI 3.80–10.15; Figure 5D; Supplementary Table S7).

The initial-centered analysis separated patterns ranging from marked decrease to increase. Initial and last lactate medians were 6.1 and 1.8 mmol/L in the largest-decrease group and 1.9 and 3.8 mmol/L in the increasing group. After adjustment for clinical covariates and log initial lactate, the increasing pattern had an OR of 2.91 (95% CI 1.75–4.85) relative to the largest-decrease pattern; corresponding ORs were 1.60 (95% CI 1.10–2.32) for moderate decrease and 2.41 (95% CI 1.62–3.58) for the stable pattern (Supplementary Table S3). This exploratory result assessed temporal shape separately from the primary burden-ordered clusters.

Excluding mechanical ventilation and vasoactive agent use from the landmark adjustment model did not materially change the association for the persistent-high pattern in MIMIC-IV (OR 6.06, 95% CI 3.73–9.84) or in the eICU re-clustered sensitivity analysis (OR 13.32, 95% CI 3.32–53.42). In the eICU-CRD median-imputation sensitivity model, which included all 429 landmark-eligible patients, the corresponding re-clustered OR was 10.58 (95% CI 3.70–30.29; Supplementary Table S19). Model-specific complete-case sample sizes are reported in Supplementary Table S20.

In the MIMIC-IV landmark risk set, 798 patients met the acute MI diagnosis definition for AMI-CS and 1,217 did not. Subsequent mortality increased across trajectory groups in both subgroups. Among patients with AMI-CS, mortality rose from 24.83% in group 1 to 79.07% in group 4; adjusted ORs were 3.10 (95% CI 1.96–4.92) for group 3 and 8.10 (95% CI 3.52–18.65) for group 4. Among patients without AMI-CS, mortality increased from 23.99% in group 1 to 69.33% in group 4; adjusted ORs were 1.61 (95% CI 1.11–2.34) for group 3 and 4.66 (95% CI 2.54–8.56) for group 4 (Figures 5E,F; Supplementary Tables S12, S13).

### External transportability

In the primary transportability analysis, landmark-eligible eICU patients were assigned directly to the fixed MIMIC-IV centroids. Subsequent mortality increased from 25.49% in group 1 to 38.06% in group 2, 45.74% in group 3, and 78.12% in group 4 (Figure 2E, Table 5; Supplementary Table S4). The adjusted model included 366 patients from 71 hospitals. Covariate-standardized mortality was 32.11% in group 1 and 75.48% in group 4, an adjusted difference of 43.36 deaths per 100 patients (95% CI 22.13–59.13); the adjusted risk ratio was 2.30 (95% CI 1.47–3.62; Table 5; Supplementary Table S27). The hospital-clustered logistic OR for group 4 was 7.71 (95% CI 2.76–21.57; Figure 2F), while Firth-penalized logistic regression gave an OR of 7.15 (95% CI 3.10–17.48; Supplementary Table S5). In the fixed-centroid median-imputation sensitivity analysis including all 429 landmark patients, adjusted mortality was 29.9% in group 1 and 72.9% in group 4; the adjusted risk difference was 43.0 deaths per 100 patients (95% CI 23.2–58.2), the risk ratio was 2.40 (95% CI 1.54–3.74), and the logistic OR was 7.62 (95% CI 2.97–19.58; Supplementary Table S5).

**Table 5:** Transportability of fixed MIMIC-IV trajectory centroids and mortality contrasts in eICU-CRD at the 24-hour landmark.

Group	*N* at landmark	Deaths	Model included, *n*	Excluded for missing covariates, *n*	Adjusted mortality, % (95% CI)	Risk difference per 100 (95% CI)	Adjusted RR (95% CI)
Group 1	102	26	79	23	32.1 (22.2–43.9)	Reference	Reference
Group 2	134	51	115	19	42.8 (34.0–51.6)	10.7 (−3.3–23.2)	1.40 (0.93–2.13)
Group 3	129	59	113	16	41.7 (32.8–51.7)	9.5 (−4.8–23.5)	1.41 (0.91–2.18)
Group 4	64	50	59	5	75.5 (58.0–87.6)	43.4 (22.1–59.1)	2.30 (1.47–3.62)

The primary transportability analysis assigned eICU-CRD patients directly to MIMIC-IV-derived centroids without re-clustering and included those alive and still hospitalized 24 h after ICU admission. Adjusted models included age, APACHE IVa score, mean arterial pressure, creatinine, mechanical ventilation, and vasoactive agent use, with hospital-clustered standard errors. Adjusted mortality is the marginal probability standardized to the covariate distribution of the complete-case model sample; risk differences compare each group with Group 1. Risk ratios were estimated using modified Poisson regression. Mortality denotes subsequent in-hospital mortality. Regular and Firth-penalized logistic estimates and an all-429-patient median-imputation sensitivity analysis are reported in Supplementary Table S5. Group 1, low-stable; Group 2, moderate-decreasing; Group 3, high-decreasing; Group 4, persistent-high. RR, risk ratio; CI, confidence interval; APACHE IVa, Acute Physiology and Chronic Health Evaluation IVa; eICU-CRD, eICU Collaborative Research Database.

Across the full eICU trajectory cohort, the median nearest-centroid distance was 0.79 and 7.77% of patients exceeded the MIMIC-IV 95th-percentile distance threshold. Agreement between fixed-centroid assignment and eICU re-clustering was 69.54%, with an adjusted Rand index of 0.447 (Supplementary Table S25). Re-clustering eICU-CRD as a sensitivity analysis produced four ordered profiles (Figure 2B; Table 2). At the landmark, mortality increased from 28.39% to 84.09% (Figure 2C), and the adjusted risk ratio for the highest pattern was 2.24 (95% CI 1.57–3.20; Supplementary Table S5). The corresponding Firth OR was 11.63 (95% CI 4.19–39.64), while the hospital-clustered logistic ORs are shown in Figure 2D; both estimates retained substantial imprecision.

Using a binary definition in the eICU-CRD landmark risk set, subsequent mortality was 34.77% without persistent hyperlactatemia and 63.78% when the last lactate within 24 h was at least 4 mmol/L. The hospital-clustered, APACHE IVa-adjusted OR was 2.36 (95% CI 1.30–4.30; *P* = 0.005; Supplementary Table S21).

## Discussion

Among patients with cardiogenic shock who underwent repeated lactate testing and survived to the prespecified 24-hour landmark, four first-day lactate patterns showed an overall gradient in subsequent in-hospital mortality, driven primarily by the high-decreasing and persistent-high patterns. Given that mortality in cardiogenic shock remains high despite contemporary treatment (20, 21), identifying patients with unresolved metabolic derangement after initial resuscitation may provide clinically relevant risk information. When the MIMIC-IV centroids were applied unchanged to eICU-CRD, the overall mortality contrast was reproduced, with the persistent-high pattern remaining clearly separated. However, the external data did not support four strictly ordered adjusted-risk strata. These findings therefore support a transportable framework for describing early lactate evolution, rather than four independently distinct mortality categories. The observed heterogeneity is consistent with findings from prospective cardiogenic shock cohorts, although the identified patterns should not be interpreted as a comprehensive classification of cardiogenic shock (22).

Previous studies have associated arterial lactate concentration and lactate clearance with outcomes in cardiogenic shock (23), while studies in broader ICU populations have suggested that lactate kinetics may be more informative than a single threshold (24, 25). A recent pilot study in acute cardiogenic pulmonary edema also linked 4-hour mean lactate clearance with adverse outcomes, although cardiogenic shock was excluded and the observation window was shorter (26). In the present study, the primary clusters were distinguished first by overall lactate burden and second by temporal shape, as reflected by the ordered initial and final lactate values and the strong predictive performance of the last lactate measurement alone. The clusters should therefore not be interpreted as subtypes defined solely by trajectory shape. Nevertheless, in the initial-lactate-centered sensitivity analysis, falling, stable, and rising patterns could be distinguished, and the rising pattern remained associated with higher mortality after adjustment for initial lactate and other clinical covariates. These findings suggest that temporal shape provides additional clinical resolution, although the overall burden of lactate remains the dominant prognostic signal.

All three internal clustering indices favored a two-cluster solution. We did not prespecify *K* = 4 as a clinical taxonomy or anchor it to predefined lactate thresholds; the 4 mmol/L threshold was used only in a separate persistent-hyperlactatemia analysis. *K* = 4 was retained before outcome modeling as a pragmatic analytical resolution because it separated low burden, decreasing, and persistent-high patterns that were reproducible across random seeds and bootstrap samples. It should therefore be viewed as an exploratory descriptive partition rather than a statistically optimal partition, a validated clinical classification, or a biological taxonomy. The consistency of the high-lactate contrast in the final-window-observed, no-edge-filling, and complete-window analyses supports the robustness of the main finding, but does not remove the judgment involved in selecting four groups.

An important consideration is that the lactate trajectory was not a baseline exposure. It reflected the combined effects of illness evolution, treatment response, organ clearance, and the clinical decision to repeat lactate measurements during the first ICU day. We therefore defined the risk set only after the complete trajectory window had been observed and interpreted the adjusted models as descriptive risk stratification at the 24-hour landmark. Variables such as SOFA score, mean arterial pressure, mechanical ventilation, and vasoactive treatment were temporally intertwined with trajectory formation and could not provide conventional control of baseline confounding. Excluding treatment variables did not materially change the estimates, but neither model can establish an independent biological effect of trajectory membership or identify a causal treatment target.

The persistent-high trajectory group had the highest mortality in both databases. This pattern may reflect ongoing tissue hypoperfusion, sustained adrenergic activation, impaired lactate clearance, failure to reverse shock physiology, or a combination of these mechanisms. Similar associations have been reported in unselected critically ill populations (27). However, the available databases could not distinguish among these mechanisms, and the observed association does not demonstrate that directly modifying the lactate trajectory would improve survival.

In the landmark prediction analysis, trajectory group improved discrimination relative to a clinical model without lactate measurements, but performed less well than the last lactate value or the combination of initial and last lactate. This finding helps define the potential role of trajectory clustering. Clustering summarizes a complex first-day course into a small number of clinically recognizable patterns, but a recent lactate measurement may be more efficient when discrimination is the sole objective. Decision-curve analysis similarly showed no consistent net-benefit advantage for trajectory grouping over simpler lactate summaries. Repeating the complete five-fold modeling pipeline across 20 random splits did not materially alter the comparison with initial lactate plus clearance, and all five prespecified algorithms showed broadly similar discriminatory performance. These results do not support adding clustering to a bedside prediction model solely to improve predictive accuracy, consistent with evidence that machine-learning methods do not consistently outperform logistic regression in clinical prediction studies (28).

Applying fixed MIMIC-IV centroids to eICU-CRD provided a stricter assessment of transportability than re-clustering because the trajectory definitions were carried unchanged into an independent dataset (29). Despite differences in participating institutions, documentation practices, and severity scoring, the persistent-high pattern remained clearly associated with greater mortality, and most eICU-CRD patients fell within the distance range observed in the derivation cohort. However, groups 2 and 3 had similar adjusted mortality, and the external findings should not be interpreted as evidence of four strictly ordered risk strata. Agreement between fixed-centroid assignment and *de novo* eICU-CRD clustering was only moderate, supporting the use of fixed-centroid assignment as the primary transportability analysis and re-clustering as a sensitivity analysis. Complete-case, all-patient imputation, hospital-clustered, and Firth regression analyses were directionally consistent, although the confidence intervals remained wide. Overall, the external analyses support transportability of the persistent-high mortality contrast across the two databases, but not external validation of the complete MIMIC-IV prediction model. Within MIMIC-IV, similar mortality gradients in patients with and without acute myocardial infarction suggest that the observed association was not confined to AMI-related cardiogenic shock.

Recent MIMIC-IV studies have used group-based trajectory modeling to characterize fluid balance in AMI-related cardiogenic shock and have externally validated mortality prediction models in this population (30, 31). The present study extends this work by examining a routinely measured metabolic marker over the first ICU day and resetting the prognostic time origin at a prespecified 24-hour landmark. We also assessed whether the findings depended on the derivation sample or preprocessing strategy by transferring fixed centroids to eICU-CRD, refitting trajectories within prediction folds, and conducting several analyses of the lactate measurement process.

Three limitations are particularly important. First, cardiogenic shock was identified retrospectively using diagnosis codes or clinical text rather than prospectively adjudicated hemodynamic criteria, and ICU admission may not have coincided precisely with shock onset. In addition, several clinically relevant variables, including SCAI shock stage, cardiac arrest, device-specific mechanical circulatory support (IABP, Impella, and VA-ECMO), timing of revascularization, liver function, and renal replacement therapy, were not consistently captured across the databases. Specimen type was also not harmonized, and arterial and venous lactate measurements could not be consistently analyzed separately; systematic specimen-related differences may have affected absolute lactate levels and trajectory assignment. These factors limited the clinical granularity of subgroup characterization and may have left residual confounding.

Second, the analysis focused on patients who underwent repeated lactate testing and survived to the prespecified 24-hour landmark. Lactate sampling frequency and the timing of the final measurement were partly influenced by clinical practice; decisions to continue or stop repeated testing may themselves reflect clinical deterioration, treatment response, or perceived stabilization. Later trajectory windows were therefore less consistently observed. Nearly half of the final-window values in the persistent-high group required edge filling, which may affect longitudinal estimates when missingness is informative (32). The final-window-observed, no-edge-filling, complete-window, and landmark analyses produced similar high-lactate contrasts, but they could not eliminate selection or measurement-process bias.

Third, lactate trajectory formation occurred in parallel with treatment and changes in illness severity during the first ICU day. The adjusted associations should therefore be interpreted as markers of risk at the 24-hour landmark rather than as estimates of an independent causal effect. External estimates were also less precise because of the smaller eICU-CRD sample, incomplete covariate availability, and differences in the clinical era represented by the database. The persistent-high mortality contrast was observed in both databases, but this finding supports transportability between these datasets rather than broad generalizability. Because the complete prediction model was not externally validated, prospective evaluation is needed before clinical implementation.

## Conclusion

Among patients with cardiogenic shock who underwent repeated lactate testing and survived to a 24-hour landmark, first-day lactate trajectories provided a clinically interpretable framework for describing lactate burden and evolution. The high-decreasing and persistent-high patterns were associated with greater subsequent mortality in MIMIC-IV, and the persistent-high mortality contrast remained clearly evident when the fixed MIMIC-IV centroids were applied to eICU-CRD. The four-group partition should therefore be interpreted as a descriptive continuum, with the most consistent transportability observed for the contrast between low-stable and persistent-high patterns rather than as four validated clinical risk classes. The exploratory initial-centered analysis suggested that temporal shape refined risk characterization beyond initial lactate level, although overall lactate burden remained the dominant prognostic signal. Simpler summaries, particularly the most recent lactate measurement, provided better discrimination and may be preferable when prediction alone is the objective. These findings support prospective evaluation of trajectory-based risk characterization at the end of the first ICU day, without establishing trajectory membership as a causal target or a substitute for clinical assessment.

## Data Availability

The original contributions presented in the study are included in the article/Supplementary Material, further inquiries can be directed to the corresponding author/s.
