# Supplementary material for: First-day lactate trajectories and subsequent in-hospital mortality in cardiogenic shock: a landmark multicohort study
Source: Front Cardiovasc Med. 2026 Sep 9;13:1948425. doi: 10.3389/fcvm.2026.1948425 (PMC13597877; doi:10.3389/fcvm.2026.1948425)
Supplement: Supplementary file 1 [file Table1.docx]

**Supplementary Tables - Frontiers in Cardiovascular Medicine**

**Supplementary Table S1. Extended machine learning performance**

| **model** | **feature_set** | **n** | **events** | **auroc** | **auprc** | **brier** | **calibration_intercept** | **calibration_slope** |
| --- | --- | --- | --- | --- | --- | --- | --- | --- |
| random_forest | foldwise_full_lactate_dynamic | 2015 | 680 | 0.737 | 0.5863 | 0.1888 | 0.0534 | 1.1013 |
| logistic_l2 | foldwise_full_lactate_dynamic | 2015 | 680 | 0.736 | 0.5891 | 0.1889 | -0.0487 | 0.9263 |
| extra_trees | foldwise_full_lactate_dynamic | 2015 | 680 | 0.727 | 0.5735 | 0.1938 | 0.2576 | 1.4021 |
| gradient_boosting | foldwise_full_lactate_dynamic | 2015 | 680 | 0.7268 | 0.5733 | 0.1915 | -0.0261 | 0.9579 |
| hist_gradient_boosting | foldwise_full_lactate_dynamic | 2015 | 680 | 0.7186 | 0.5727 | 0.1965 | -0.1668 | 0.6802 |

Note. Performance was estimated using 5-fold stratified cross-validation among MIMIC-IV patients alive and still hospitalized at the 24-hour landmark. All models used the full lactate dynamic feature set. Events indicate in-hospital deaths. AUROC, area under the receiver operating characteristic curve; AUPRC, area under the precision-recall curve; Brier, Brier score; L2, L2-penalized logistic regression. Source files: Supplementary_Table_S1_Extended_machine_learning_performance.csv.

**Supplementary Table S2. Missingness summary in MIMIC-IV and eICU-CRD**

| **cohort** | **variable** | **n_total** | **n_missing** | **missing_pct** |
| --- | --- | --- | --- | --- |
| MIMIC-IV | age | 2181 | 0 | 0 |
| MIMIC-IV | gender | 2181 | 0 | 0 |
| MIMIC-IV | lactate_n_24h | 2181 | 0 | 0 |
| MIMIC-IV | initial_lactate_24h | 2181 | 0 | 0 |
| MIMIC-IV | last_lactate_24h | 2181 | 0 | 0 |
| MIMIC-IV | peak_lactate_24h | 2181 | 0 | 0 |
| MIMIC-IV | lactate_clearance_24h | 2181 | 0 | 0 |
| MIMIC-IV | sofa | 2181 | 0 | 0 |
| MIMIC-IV | sapsii | 2181 | 0 | 0 |
| MIMIC-IV | oasis | 2181 | 0 | 0 |
| MIMIC-IV | charlson_comorbidity_index | 2181 | 0 | 0 |
| MIMIC-IV | mbp_mean | 2181 | 4 | 0.18 |
| MIMIC-IV | creatinine_max | 2181 | 0 | 0 |
| MIMIC-IV | mechvent_24h | 2181 | 0 | 0 |
| MIMIC-IV | vasoactive_24h | 2181 | 0 | 0 |
| MIMIC-IV | trajectory_group | 2181 | 0 | 0 |
| eICU-CRD | age | 476 | 0 | 0 |
| eICU-CRD | gender | 476 | 0 | 0 |
| eICU-CRD | lactate_n_24h | 476 | 0 | 0 |
| eICU-CRD | initial_lactate_24h | 476 | 0 | 0 |
| eICU-CRD | last_lactate_24h | 476 | 0 | 0 |
| eICU-CRD | peak_lactate_24h | 476 | 0 | 0 |
| eICU-CRD | lactate_clearance_24h | 476 | 0 | 0 |
| eICU-CRD | acutephysiologyscore | 476 | 68 | 14.29 |
| eICU-CRD | apachescore | 476 | 68 | 14.29 |
| eICU-CRD | meanbp | 476 | 24 | 5.04 |
| eICU-CRD | creatinine | 476 | 24 | 5.04 |
| eICU-CRD | vent | 476 | 24 | 5.04 |
| eICU-CRD | vasoactive_24h | 476 | 0 | 0 |
| eICU-CRD | trajectory_group | 476 | 0 | 0 |

Note. Missingness is reported within the analytic trajectory cohorts. n_total is the denominator for each cohort-variable pair; n_missing and missing_pct report unavailable values before model-specific complete-case analysis. MIMIC-IV, Medical Information Mart for Intensive Care IV; eICU-CRD, eICU Collaborative Research Database. Source files: Supplementary_Table_S2_Missingness_summary.csv.

**Supplementary Table S3. Trajectory sensitivity analyses in MIMIC-IV**

| **scenario** | **k** | **min_lactate_count** | **trajectory_group** | **n** | **deaths** | **mortality_pct** | **initial_lactate_median** | **last_lactate_median** | **peak_lactate_median** | **adjusted_or_vs_group1** | **ci95** | **p_value** | **model_n** |
| --- | --- | --- | --- | --- | --- | --- | --- | --- | --- | --- | --- | --- | --- |
| k2_min2 | 2 | 2 | 1 | 1595 | 453 | 28.4 | 2.2 | 1.6 | 2.7 | reference | reference | reference |  |
| k2_min2 | 2 | 2 | 2 | 586 | 391 | 66.72 | 6.4 | 4.9 | 9.1 | 3.78 | 3.00-4.77 | 1.2258005484990642e-29 |  |
| k3_min2 | 3 | 2 | 1 | 1189 | 311 | 26.16 | 1.9 | 1.4 | 2.3 | reference | reference | reference |  |
| k3_min2 | 3 | 2 | 2 | 716 | 307 | 42.88 | 3.9 | 2.7 | 5.6 | 1.69 | 1.36-2.10 | 1.7726098497235127e-06 |  |
| k3_min2 | 3 | 2 | 3 | 276 | 226 | 81.88 | 8.2 | 9.2 | 12.3 | 8.92 | 6.17-12.91 | 3.829730985069999e-31 |  |
| k5_min2 | 5 | 2 | 1 | 554 | 140 | 25.27 | 1.4 | 1.2 | 1.6 | reference | reference | reference |  |
| k5_min2 | 5 | 2 | 2 | 748 | 214 | 28.61 | 2.45 | 1.8 | 2.9 | 1.04 | 0.80-1.35 | 0.7693918153093333 |  |
| k5_min2 | 5 | 2 | 3 | 469 | 184 | 39.23 | 3.8 | 2.7 | 5.3 | 1.49 | 1.12-2.00 | 0.006683401748015097 |  |
| k5_min2 | 5 | 2 | 4 | 262 | 170 | 64.89 | 6.4 | 4.9 | 9 | 3.95 | 2.76-5.66 | 5.812409531745458e-14 |  |
| k5_min2 | 5 | 2 | 5 | 148 | 136 | 91.89 | 9.6 | 12.95 | 14.75 | 21.15 | 11.02-40.59 | 4.4709128589541534e-20 |  |
| k4_min3 | 4 | 3 | 1 | 578 | 135 | 23.36 | 1.6 | 1.3 | 2 | reference | reference | reference |  |
| k4_min3 | 4 | 3 | 2 | 638 | 204 | 31.97 | 2.9 | 2 | 3.7 | 1.3 | 0.99-1.70 | 0.05840788930167527 |  |
| k4_min3 | 4 | 3 | 3 | 396 | 201 | 50.76 | 4.75 | 3.3 | 7.3 | 2.46 | 1.81-3.34 | 7.146684376737613e-09 |  |
| k4_min3 | 4 | 3 | 4 | 202 | 170 | 84.16 | 8.9 | 10.2 | 13.2 | 11.45 | 7.20-18.22 | 8.362579035512484e-25 |  |
| k4_round2_at_least3_observed_final_window_observed_landmark | 4 | 2 | 1 | 385 | 95 | 24.6753 | 1.7 | 1.3 | 2.1 | reference | reference | reference | 1164 |
| k4_round2_at_least3_observed_final_window_observed_landmark | 4 | 2 | 2 | 397 | 125 | 31.4861 | 3.1 | 2.1 | 4 | 1.1887825512308152 | 0.85-1.66 | 0.30835806288106304 | 1164 |
| k4_round2_at_least3_observed_final_window_observed_landmark | 4 | 2 | 3 | 275 | 138 | 50.1818 | 4.9 | 3.3 | 7.6 | 2.4466608772073783 | 1.70-3.53 | 1.648019300671399e-06 | 1164 |
| k4_round2_at_least3_observed_final_window_observed_landmark | 4 | 2 | 4 | 108 | 81 | 75 | 8.9 | 7.5 | 12.7 | 6.863039661835848 | 3.97-11.87 | 5.6417077979931085e-12 | 1164 |
| k4_round2_no_edge_filling_first_and_final_observed_landmark | 4 | 2 | 1 | 367 | 90 | 24.5232 | 1.7 | 1.3 | 2.2 | reference | reference | reference | 1109 |
| k4_round2_no_edge_filling_first_and_final_observed_landmark | 4 | 2 | 2 | 375 | 119 | 31.7333 | 3 | 2.1 | 4 | 1.2090731222953714 | 0.86-1.70 | 0.2764841396872987 | 1109 |
| k4_round2_no_edge_filling_first_and_final_observed_landmark | 4 | 2 | 3 | 263 | 131 | 49.8099 | 4.9 | 3.3 | 7.6 | 2.4301166250508337 | 1.67-3.53 | 3.36475003159632e-06 | 1109 |
| k4_round2_no_edge_filling_first_and_final_observed_landmark | 4 | 2 | 4 | 105 | 80 | 76.1905 | 8.9 | 7.5 | 12.7 | 7.355145214665842 | 4.19-12.92 | 3.794787307071803e-12 | 1109 |
| k4_initial_centered_log_shape_landmark | 4 | 2 | 1 | 213 | 75 | 35.2113 | 6.1 | 1.8 | 6.5 | reference | reference | reference | 2013 |
| k4_initial_centered_log_shape_landmark | 4 | 2 | 2 | 671 | 219 | 32.6379 | 3 | 1.7 | 3.4 | 1.6001787570351587 | 1.10-2.32 | 0.012962646923388443 | 2013 |
| k4_initial_centered_log_shape_landmark | 4 | 2 | 3 | 970 | 318 | 32.7835 | 1.9 | 1.85 | 2.4 | 2.408827121215414 | 1.62-3.58 | 1.2875761643428832e-05 | 2013 |
| k4_initial_centered_log_shape_landmark | 4 | 2 | 4 | 161 | 68 | 42.236 | 1.9 | 3.8 | 6.5 | 2.909599630687842 | 1.75-4.85 | 4.219146311542032e-05 | 2013 |

Note. Sensitivity scenarios include K=2, K=3, and K=5 clustering, restriction to patients with at least three lactate measurements, restriction to at least three directly observed windows with an observed 18-24-hour window, an analysis requiring observed first and final windows with no edge filling, and an initial-centered shape analysis. For the initial-centered analysis, each patient's 0-6-hour log-lactate value was subtracted from the three later log-lactate windows before K=4 clustering; groups were ordered from the largest decrease to the largest increase, and models additionally adjusted for log initial lactate. For all other scenarios, Group 1 is the lowest lactate-burden group. Group 1 is the reference group for adjusted ORs. P values are two-sided. OR, odds ratio; CI, confidence interval. Source files: Supplementary_Table_S3_Trajectory_sensitivity_analyses.csv.

**Supplementary Table S4. eICU fixed-centroid transportability assessment using MIMIC-IV-derived centroids.**

| **trajectory_group** | **n** | **deaths** | **mortality_pct** | **initial_lactate_median** | **last_lactate_median** | **peak_lactate_median** |
| --- | --- | --- | --- | --- | --- | --- |
| 1 | 102 | 26 | 25.49 | 1.6 | 1.3 | 1.75 |
| 2 | 134 | 51 | 38.06 | 3.1 | 1.98 | 3.44 |
| 3 | 129 | 59 | 45.74 | 5.7 | 3.8 | 6.9 |
| 4 | 64 | 50 | 78.12 | 10.1 | 9.55 | 12.55 |

Note. Patients alive and still hospitalized at the 24-hour landmark were assigned to the nearest MIMIC-IV-derived lactate trajectory centroid without re-clustering. Group numbers follow the MIMIC-IV ordering from lowest to highest 24-hour lactate burden. Mortality denotes subsequent in-hospital mortality. Lactate values are reported in mmol/L. Source files: Supplementary_Table_S4_eICU_MIMIC_centroid_validation.csv.

**Supplementary Table S5. Adjusted eICU landmark associations using fixed MIMIC-IV centroids, all-patient imputation, and eICU re-clustering**

| **Assignment method** | **Group** | **N at landmark** | **Deaths** | **Model included, n** | **Adjusted mortality, % (95% CI)** | **Risk difference per 100 (95% CI)** | **Adjusted RR (95% CI)** | **Regular OR (95% CI)** | **Firth OR (95% CI)** |
| --- | --- | --- | --- | --- | --- | --- | --- | --- | --- |
| Fixed MIMIC-IV centroids (primary) | Group 1 | 102 | 26 | 79 | 32.1 (22.2-43.9) | Reference | Reference | Reference | Reference |
| Fixed MIMIC-IV centroids (primary) | Group 2 | 134 | 51 | 115 | 42.8 (34.0-51.6) | 10.7 (-3.3-23.2) | 1.40 (0.93-2.13) | 1.65 (0.87-3.12) | 1.62 (0.86-3.13) |
| Fixed MIMIC-IV centroids (primary) | Group 3 | 129 | 59 | 113 | 41.7 (32.8-51.7) | 9.5 (-4.8-23.5) | 1.41 (0.91-2.18) | 1.57 (0.78-3.17) | 1.55 (0.80-3.02) |
| Fixed MIMIC-IV centroids (primary) | Group 4 | 64 | 50 | 59 | 75.5 (58.0-87.6) | 43.4 (22.1-59.1) | 2.30 (1.47-3.62) | 7.71 (2.76-21.57) | 7.15 (3.10-17.48) |
| Fixed MIMIC-IV centroids (median-imputation sensitivity) | Group 1 | 102 | 26 | 102 | 29.9 (21.0-41.0) | Reference | Reference | Reference | Reference |
| Fixed MIMIC-IV centroids (median-imputation sensitivity) | Group 2 | 134 | 51 | 134 | 40.3 (33.8-47.7) | 10.4 (-2.0-21.5) | 1.42 (0.95-2.11) | 1.66 (0.91-3.03) | Not estimated |
| Fixed MIMIC-IV centroids (median-imputation sensitivity) | Group 3 | 129 | 59 | 129 | 43.2 (34.7-52.3) | 13.3 (0.0-25.6) | 1.55 (1.04-2.31) | 1.89 (1.00-3.59) | Not estimated |
| Fixed MIMIC-IV centroids (median-imputation sensitivity) | Group 4 | 64 | 50 | 64 | 72.9 (57.2-84.2) | 43.0 (23.2-58.2) | 2.40 (1.54-3.74) | 7.62 (2.97-19.58) | Not estimated |
| eICU re-clustering (sensitivity) | Group 1 | 155 | 44 | 126 | 34.7 (27.4-43.1) | Reference | Reference | Reference | Reference |
| eICU re-clustering (sensitivity) | Group 2 | 138 | 53 | 121 | 40.9 (33.1-49.2) | 6.2 (-3.9-16.0) | 1.23 (0.92-1.64) | 1.33 (0.83-2.12) | 1.32 (0.76-2.30) |
| eICU re-clustering (sensitivity) | Group 3 | 92 | 52 | 79 | 49.7 (35.8-63.3) | 15.0 (-0.7-30.3) | 1.51 (1.05-2.16) | 1.96 (0.97-3.97) | 1.93 (1.04-3.61) |
| eICU re-clustering (sensitivity) | Group 4 | 44 | 37 | 40 | 85.6 (63.4-95.4) | 50.9 (26.7-64.1) | 2.24 (1.57-3.20) | 13.41 (3.34-53.90) | 11.63 (4.19-39.64) |

Note. The primary analysis assigned patients to fixed MIMIC-IV-derived centroids using complete covariate records. A fixed-centroid sensitivity analysis retained all 429 landmark patients by median-imputing APACHE IVa score, mean arterial pressure, creatinine, and ventilation status and adding indicators for the two observed missingness patterns; eICU re-clustering was a separate sensitivity analysis. Models included age, APACHE IVa score, mean arterial pressure, creatinine, mechanical ventilation, and vasoactive agent use. Regular logistic and modified Poisson estimates used hospital-clustered standard errors. Firth logistic regression was an unclustered small-sample sensitivity analysis. Group 1 was the reference group. OR, odds ratio; RR, risk ratio; CI, confidence interval; APACHE IVa, Acute Physiology and Chronic Health Evaluation IVa. Source files: Supplementary_Table_S5_eICU_MIMIC_centroid_adjusted_associations.csv.

**Supplementary Table S6. Bootstrap increments in AUROC and AUPRC**

| **comparison** | **n_bootstrap** | **delta_auroc** | **delta_auroc_ci95** | **delta_auprc** | **delta_auprc_ci95** |
| --- | --- | --- | --- | --- | --- |
| base_plus_trajectory vs clinical_base | 500 | 0.0214 | 0.0110-0.0325 | 0.0397 | 0.0193-0.0625 |
| base_plus_full_lactate vs clinical_base | 500 | 0.033 | 0.0199-0.0448 | 0.0568 | 0.0297-0.0794 |
| base_plus_full_lactate vs base_plus_trajectory | 500 | 0.0116 | 0.0036-0.0198 | 0.0171 | -0.0017-0.0355 |

Note. Bootstrap estimates used 500 patient-level resamples from the 24-hour landmark risk set. Delta values are absolute performance differences between the compared models. Confidence intervals are empirical 95% bootstrap intervals. AUROC, area under the receiver operating characteristic curve; AUPRC, area under the precision-recall curve. Source files: Supplementary_Table_S6_Bootstrap_prediction_increments.csv.

**Supplementary Table S7. Observation-window sensitivity analysis excluding ICU stays shorter than 24 hours**

| **trajectory_group** | **n** | **deaths** | **mortality_pct** | **initial_lactate_median** | **last_lactate_median** | **peak_lactate_median** |
| --- | --- | --- | --- | --- | --- | --- |
| 1 | 703 | 177 | 25.18 | 1.5 | 1.3 | 1.8 |
| 2 | 743 | 224 | 30.15 | 2.9 | 2 | 3.5 |
| 3 | 387 | 188 | 48.58 | 4.9 | 3.3 | 7.2 |
| 4 | 124 | 94 | 75.81 | 8.45 | 7.8 | 12.7 |

Note. This sensitivity analysis excluded patients with ICU length of stay shorter than 24 hours to reduce bias from incomplete early observation. Group 1, low-stable; Group 2, moderate-decreasing; Group 3, high-decreasing; Group 4, persistent-high. Mortality denotes in-hospital mortality. Lactate values are reported in mmol/L. Source files: Supplementary_Table_S7_Observation_window_sensitivity.csv.

**Supplementary Table S8. Calibration and decision curve source data**

**Calibration curve source data**

| **mean_predicted** | **observed** | **n** | **model** |
| --- | --- | --- | --- |
| 0.0973 | 0.104 | 202 | clinical_base |
| 0.1598 | 0.1642 | 201 | clinical_base |
| 0.2088 | 0.1485 | 202 | clinical_base |
| 0.2523 | 0.2786 | 201 | clinical_base |
| 0.2963 | 0.3515 | 202 | clinical_base |
| 0.3439 | 0.3383 | 201 | clinical_base |
| 0.3909 | 0.4129 | 201 | clinical_base |
| 0.4494 | 0.4505 | 202 | clinical_base |
| 0.5255 | 0.4925 | 201 | clinical_base |
| 0.6532 | 0.6337 | 202 | clinical_base |
| 0.0921 | 0.0842 | 202 | base_plus_trajectory |
| 0.1501 | 0.1542 | 201 | base_plus_trajectory |
| 0.1951 | 0.1881 | 202 | base_plus_trajectory |
| 0.2382 | 0.2338 | 201 | base_plus_trajectory |
| 0.281 | 0.2475 | 202 | base_plus_trajectory |
| 0.3288 | 0.4279 | 201 | base_plus_trajectory |
| 0.3843 | 0.3831 | 201 | base_plus_trajectory |
| 0.4496 | 0.4406 | 202 | base_plus_trajectory |
| 0.5354 | 0.5274 | 201 | base_plus_trajectory |
| 0.7171 | 0.6881 | 202 | base_plus_trajectory |
| 0.0883 | 0.0743 | 202 | base_plus_full_lactate |
| 0.1451 | 0.1343 | 201 | base_plus_full_lactate |
| 0.1898 | 0.1683 | 202 | base_plus_full_lactate |
| 0.233 | 0.2338 | 201 | base_plus_full_lactate |
| 0.2759 | 0.302 | 202 | base_plus_full_lactate |
| 0.3226 | 0.3781 | 201 | base_plus_full_lactate |
| 0.3766 | 0.408 | 201 | base_plus_full_lactate |
| 0.4435 | 0.4158 | 202 | base_plus_full_lactate |
| 0.541 | 0.5323 | 201 | base_plus_full_lactate |
| 0.7637 | 0.7277 | 202 | base_plus_full_lactate |

**Decision curve source data**

| **threshold** | **net_benefit** | **treat_all** | **treat_none** | **model** |
| --- | --- | --- | --- | --- |
| 0.05 | 0.3027 | 0.3026 | 0 | clinical_base |
| 0.06 | 0.2946 | 0.2952 | 0 | clinical_base |
| 0.07 | 0.2867 | 0.2876 | 0 | clinical_base |
| 0.08 | 0.2779 | 0.2799 | 0 | clinical_base |
| 0.09 | 0.2709 | 0.2719 | 0 | clinical_base |
| 0.1 | 0.2638 | 0.2639 | 0 | clinical_base |
| 0.11 | 0.2571 | 0.2556 | 0 | clinical_base |
| 0.12 | 0.2496 | 0.2471 | 0 | clinical_base |
| 0.13 | 0.2415 | 0.2385 | 0 | clinical_base |
| 0.14 | 0.233 | 0.2296 | 0 | clinical_base |
| 0.15 | 0.226 | 0.2206 | 0 | clinical_base |
| 0.16 | 0.2181 | 0.2113 | 0 | clinical_base |
| 0.17 | 0.2118 | 0.2018 | 0 | clinical_base |
| 0.18 | 0.2038 | 0.192 | 0 | clinical_base |
| 0.19 | 0.1971 | 0.1821 | 0 | clinical_base |
| 0.2 | 0.1911 | 0.1718 | 0 | clinical_base |
| 0.21 | 0.1857 | 0.1614 | 0 | clinical_base |
| 0.22 | 0.1782 | 0.1506 | 0 | clinical_base |
| 0.23 | 0.1736 | 0.1396 | 0 | clinical_base |
| 0.24 | 0.1684 | 0.1282 | 0 | clinical_base |
| 0.25 | 0.1636 | 0.1166 | 0 | clinical_base |
| 0.26 | 0.1541 | 0.1047 | 0 | clinical_base |
| 0.27 | 0.1466 | 0.0924 | 0 | clinical_base |
| 0.28 | 0.1391 | 0.0798 | 0 | clinical_base |
| 0.29 | 0.133 | 0.0669 | 0 | clinical_base |
| 0.3 | 0.1246 | 0.0535 | 0 | clinical_base |
| 0.31 | 0.1161 | 0.0398 | 0 | clinical_base |
| 0.32 | 0.1068 | 0.0257 | 0 | clinical_base |
| 0.33 | 0.1029 | 0.0111 | 0 | clinical_base |
| 0.34 | 0.0979 | -0.0038 | 0 | clinical_base |
| 0.35 | 0.0913 | -0.0193 | 0 | clinical_base |
| 0.36 | 0.0846 | -0.0352 | 0 | clinical_base |
| 0.37 | 0.0811 | -0.0516 | 0 | clinical_base |
| 0.38 | 0.0751 | -0.0686 | 0 | clinical_base |
| 0.39 | 0.0664 | -0.0861 | 0 | clinical_base |
| 0.4 | 0.0617 | -0.1042 | 0 | clinical_base |
| 0.41 | 0.0571 | -0.1229 | 0 | clinical_base |
| 0.42 | 0.053 | -0.1423 | 0 | clinical_base |
| 0.43 | 0.0505 | -0.1623 | 0 | clinical_base |
| 0.44 | 0.0463 | -0.1831 | 0 | clinical_base |
| 0.45 | 0.0437 | -0.2046 | 0 | clinical_base |
| 0.46 | 0.0414 | -0.2269 | 0 | clinical_base |
| 0.47 | 0.0318 | -0.2501 | 0 | clinical_base |
| 0.48 | 0.0322 | -0.2741 | 0 | clinical_base |
| 0.49 | 0.032 | -0.2991 | 0 | clinical_base |
| 0.5 | 0.0263 | -0.3251 | 0 | clinical_base |
| 0.51 | 0.0204 | -0.3521 | 0 | clinical_base |
| 0.52 | 0.02 | -0.3803 | 0 | clinical_base |
| 0.53 | 0.0203 | -0.4096 | 0 | clinical_base |
| 0.54 | 0.0198 | -0.4403 | 0 | clinical_base |
| 0.55 | 0.0151 | -0.4723 | 0 | clinical_base |
| 0.56 | 0.0119 | -0.5058 | 0 | clinical_base |
| 0.57 | 0.0162 | -0.5408 | 0 | clinical_base |
| 0.58 | 0.0133 | -0.5775 | 0 | clinical_base |
| 0.59 | 0.005 | -0.6159 | 0 | clinical_base |
| 0.6 | 0.0057 | -0.6563 | 0 | clinical_base |
| 0.61 | 0.003 | -0.6988 | 0 | clinical_base |
| 0.62 | 9.66e-04 | -0.7435 | 0 | clinical_base |
| 0.63 | 0.0025 | -0.7906 | 0 | clinical_base |
| 0.64 | 0.0053 | -0.8404 | 0 | clinical_base |
| 0.65 | 0.0062 | -0.8929 | 0 | clinical_base |
| 0.66 | 0.0027 | -0.9486 | 0 | clinical_base |
| 0.67 | 0.0037 | -1.0077 | 0 | clinical_base |
| 0.68 | 0.006 | -1.0704 | 0 | clinical_base |
| 0.69 | 0.0038 | -1.1372 | 0 | clinical_base |
| 0.7 | 0.0033 | -1.2084 | 0 | clinical_base |
| 0.71 | 0.0042 | -1.2846 | 0 | clinical_base |
| 0.72 | 0.0057 | -1.3662 | 0 | clinical_base |
| 0.73 | 0.0037 | -1.4538 | 0 | clinical_base |
| 0.74 | 8.78e-04 | -1.5482 | 0 | clinical_base |
| 0.75 | 0.002 | -1.6501 | 0 | clinical_base |
| 0.76 | 0.0017 | -1.7605 | 0 | clinical_base |
| 0.77 | 0.0038 | -1.8806 | 0 | clinical_base |
| 0.78 | 0.0017 | -2.0115 | 0 | clinical_base |
| 0.79 | 6.14e-04 | -2.1549 | 0 | clinical_base |
| 0.8 | -4.96e-04 | -2.3127 | 0 | clinical_base |
| 0.05 | 0.3027 | 0.3026 | 0 | base_plus_trajectory |
| 0.06 | 0.2953 | 0.2952 | 0 | base_plus_trajectory |
| 0.07 | 0.2869 | 0.2876 | 0 | base_plus_trajectory |
| 0.08 | 0.2799 | 0.2799 | 0 | base_plus_trajectory |
| 0.09 | 0.2737 | 0.2719 | 0 | base_plus_trajectory |
| 0.1 | 0.2662 | 0.2639 | 0 | base_plus_trajectory |
| 0.11 | 0.2578 | 0.2556 | 0 | base_plus_trajectory |
| 0.12 | 0.2501 | 0.2471 | 0 | base_plus_trajectory |
| 0.13 | 0.2436 | 0.2385 | 0 | base_plus_trajectory |
| 0.14 | 0.2356 | 0.2296 | 0 | base_plus_trajectory |
| 0.15 | 0.2298 | 0.2206 | 0 | base_plus_trajectory |
| 0.16 | 0.2218 | 0.2113 | 0 | base_plus_trajectory |
| 0.17 | 0.2156 | 0.2018 | 0 | base_plus_trajectory |
| 0.18 | 0.2055 | 0.192 | 0 | base_plus_trajectory |
| 0.19 | 0.1995 | 0.1821 | 0 | base_plus_trajectory |
| 0.2 | 0.1945 | 0.1718 | 0 | base_plus_trajectory |
| 0.21 | 0.1872 | 0.1614 | 0 | base_plus_trajectory |
| 0.22 | 0.1799 | 0.1506 | 0 | base_plus_trajectory |
| 0.23 | 0.1734 | 0.1396 | 0 | base_plus_trajectory |
| 0.24 | 0.168 | 0.1282 | 0 | base_plus_trajectory |
| 0.25 | 0.1621 | 0.1166 | 0 | base_plus_trajectory |
| 0.26 | 0.1559 | 0.1047 | 0 | base_plus_trajectory |
| 0.27 | 0.1497 | 0.0924 | 0 | base_plus_trajectory |
| 0.28 | 0.1496 | 0.0798 | 0 | base_plus_trajectory |
| 0.29 | 0.1436 | 0.0669 | 0 | base_plus_trajectory |
| 0.3 | 0.1378 | 0.0535 | 0 | base_plus_trajectory |
| 0.31 | 0.1336 | 0.0398 | 0 | base_plus_trajectory |
| 0.32 | 0.1226 | 0.0257 | 0 | base_plus_trajectory |
| 0.33 | 0.1118 | 0.0111 | 0 | base_plus_trajectory |
| 0.34 | 0.103 | -0.0038 | 0 | base_plus_trajectory |
| 0.35 | 0.0982 | -0.0193 | 0 | base_plus_trajectory |
| 0.36 | 0.0927 | -0.0352 | 0 | base_plus_trajectory |
| 0.37 | 0.0875 | -0.0516 | 0 | base_plus_trajectory |
| 0.38 | 0.0798 | -0.0686 | 0 | base_plus_trajectory |
| 0.39 | 0.0759 | -0.0861 | 0 | base_plus_trajectory |
| 0.4 | 0.0726 | -0.1042 | 0 | base_plus_trajectory |
| 0.41 | 0.0722 | -0.1229 | 0 | base_plus_trajectory |
| 0.42 | 0.069 | -0.1423 | 0 | base_plus_trajectory |
| 0.43 | 0.0651 | -0.1623 | 0 | base_plus_trajectory |
| 0.44 | 0.0626 | -0.1831 | 0 | base_plus_trajectory |
| 0.45 | 0.06 | -0.2046 | 0 | base_plus_trajectory |
| 0.46 | 0.0547 | -0.2269 | 0 | base_plus_trajectory |
| 0.47 | 0.0543 | -0.2501 | 0 | base_plus_trajectory |
| 0.48 | 0.049 | -0.2741 | 0 | base_plus_trajectory |
| 0.49 | 0.0451 | -0.2991 | 0 | base_plus_trajectory |
| 0.5 | 0.0442 | -0.3251 | 0 | base_plus_trajectory |
| 0.51 | 0.0451 | -0.3521 | 0 | base_plus_trajectory |
| 0.52 | 0.0413 | -0.3803 | 0 | base_plus_trajectory |
| 0.53 | 0.0404 | -0.4096 | 0 | base_plus_trajectory |
| 0.54 | 0.0383 | -0.4403 | 0 | base_plus_trajectory |
| 0.55 | 0.0351 | -0.4723 | 0 | base_plus_trajectory |
| 0.56 | 0.03 | -0.5058 | 0 | base_plus_trajectory |
| 0.57 | 0.0307 | -0.5408 | 0 | base_plus_trajectory |
| 0.58 | 0.0269 | -0.5775 | 0 | base_plus_trajectory |
| 0.59 | 0.0277 | -0.6159 | 0 | base_plus_trajectory |
| 0.6 | 0.0226 | -0.6563 | 0 | base_plus_trajectory |
| 0.61 | 0.0194 | -0.6988 | 0 | base_plus_trajectory |
| 0.62 | 0.0198 | -0.7435 | 0 | base_plus_trajectory |
| 0.63 | 0.0179 | -0.7906 | 0 | base_plus_trajectory |
| 0.64 | 0.0158 | -0.8404 | 0 | base_plus_trajectory |
| 0.65 | 0.0169 | -0.8929 | 0 | base_plus_trajectory |
| 0.66 | 0.0125 | -0.9486 | 0 | base_plus_trajectory |
| 0.67 | 0.0119 | -1.0077 | 0 | base_plus_trajectory |
| 0.68 | 0.0084 | -1.0704 | 0 | base_plus_trajectory |
| 0.69 | 0.0039 | -1.1372 | 0 | base_plus_trajectory |
| 0.7 | 0.0071 | -1.2084 | 0 | base_plus_trajectory |
| 0.71 | 0.0056 | -1.2846 | 0 | base_plus_trajectory |
| 0.72 | 0.0069 | -1.3662 | 0 | base_plus_trajectory |
| 0.73 | 0.0071 | -1.4538 | 0 | base_plus_trajectory |
| 0.74 | 0.0071 | -1.5482 | 0 | base_plus_trajectory |
| 0.75 | 0.0055 | -1.6501 | 0 | base_plus_trajectory |
| 0.76 | 0.0055 | -1.7605 | 0 | base_plus_trajectory |
| 0.77 | 0.0042 | -1.8806 | 0 | base_plus_trajectory |
| 0.78 | 0.0013 | -2.0115 | 0 | base_plus_trajectory |
| 0.79 | -4.25e-04 | -2.1549 | 0 | base_plus_trajectory |
| 0.8 | -0.0015 | -2.3127 | 0 | base_plus_trajectory |
| 0.05 | 0.3023 | 0.3026 | 0 | base_plus_full_lactate |
| 0.06 | 0.2949 | 0.2952 | 0 | base_plus_full_lactate |
| 0.07 | 0.2862 | 0.2876 | 0 | base_plus_full_lactate |
| 0.08 | 0.2797 | 0.2799 | 0 | base_plus_full_lactate |
| 0.09 | 0.273 | 0.2719 | 0 | base_plus_full_lactate |
| 0.1 | 0.2667 | 0.2639 | 0 | base_plus_full_lactate |
| 0.11 | 0.2581 | 0.2556 | 0 | base_plus_full_lactate |
| 0.12 | 0.2521 | 0.2471 | 0 | base_plus_full_lactate |
| 0.13 | 0.2457 | 0.2385 | 0 | base_plus_full_lactate |
| 0.14 | 0.239 | 0.2296 | 0 | base_plus_full_lactate |
| 0.15 | 0.2316 | 0.2206 | 0 | base_plus_full_lactate |
| 0.16 | 0.2247 | 0.2113 | 0 | base_plus_full_lactate |
| 0.17 | 0.2174 | 0.2018 | 0 | base_plus_full_lactate |
| 0.18 | 0.2089 | 0.192 | 0 | base_plus_full_lactate |
| 0.19 | 0.2035 | 0.1821 | 0 | base_plus_full_lactate |
| 0.2 | 0.1976 | 0.1718 | 0 | base_plus_full_lactate |
| 0.21 | 0.1934 | 0.1614 | 0 | base_plus_full_lactate |
| 0.22 | 0.1849 | 0.1506 | 0 | base_plus_full_lactate |
| 0.23 | 0.178 | 0.1396 | 0 | base_plus_full_lactate |
| 0.24 | 0.1752 | 0.1282 | 0 | base_plus_full_lactate |
| 0.25 | 0.1681 | 0.1166 | 0 | base_plus_full_lactate |
| 0.26 | 0.1614 | 0.1047 | 0 | base_plus_full_lactate |
| 0.27 | 0.1546 | 0.0924 | 0 | base_plus_full_lactate |
| 0.28 | 0.1471 | 0.0798 | 0 | base_plus_full_lactate |
| 0.29 | 0.144 | 0.0669 | 0 | base_plus_full_lactate |
| 0.3 | 0.1377 | 0.0535 | 0 | base_plus_full_lactate |
| 0.31 | 0.1285 | 0.0398 | 0 | base_plus_full_lactate |
| 0.32 | 0.1183 | 0.0257 | 0 | base_plus_full_lactate |
| 0.33 | 0.113 | 0.0111 | 0 | base_plus_full_lactate |
| 0.34 | 0.1091 | -0.0038 | 0 | base_plus_full_lactate |
| 0.35 | 0.1058 | -0.0193 | 0 | base_plus_full_lactate |
| 0.36 | 0.0992 | -0.0352 | 0 | base_plus_full_lactate |
| 0.37 | 0.0966 | -0.0516 | 0 | base_plus_full_lactate |
| 0.38 | 0.0891 | -0.0686 | 0 | base_plus_full_lactate |
| 0.39 | 0.0836 | -0.0861 | 0 | base_plus_full_lactate |
| 0.4 | 0.0809 | -0.1042 | 0 | base_plus_full_lactate |
| 0.41 | 0.0757 | -0.1229 | 0 | base_plus_full_lactate |
| 0.42 | 0.073 | -0.1423 | 0 | base_plus_full_lactate |
| 0.43 | 0.0692 | -0.1623 | 0 | base_plus_full_lactate |
| 0.44 | 0.0657 | -0.1831 | 0 | base_plus_full_lactate |
| 0.45 | 0.0645 | -0.2046 | 0 | base_plus_full_lactate |
| 0.46 | 0.0624 | -0.2269 | 0 | base_plus_full_lactate |
| 0.47 | 0.0599 | -0.2501 | 0 | base_plus_full_lactate |
| 0.48 | 0.0595 | -0.2741 | 0 | base_plus_full_lactate |
| 0.49 | 0.0534 | -0.2991 | 0 | base_plus_full_lactate |
| 0.5 | 0.0521 | -0.3251 | 0 | base_plus_full_lactate |
| 0.51 | 0.0522 | -0.3521 | 0 | base_plus_full_lactate |
| 0.52 | 0.0526 | -0.3803 | 0 | base_plus_full_lactate |
| 0.53 | 0.0464 | -0.4096 | 0 | base_plus_full_lactate |
| 0.54 | 0.0457 | -0.4403 | 0 | base_plus_full_lactate |
| 0.55 | 0.041 | -0.4723 | 0 | base_plus_full_lactate |
| 0.56 | 0.0398 | -0.5058 | 0 | base_plus_full_lactate |
| 0.57 | 0.037 | -0.5408 | 0 | base_plus_full_lactate |
| 0.58 | 0.0376 | -0.5775 | 0 | base_plus_full_lactate |
| 0.59 | 0.0362 | -0.6159 | 0 | base_plus_full_lactate |
| 0.6 | 0.0315 | -0.6563 | 0 | base_plus_full_lactate |
| 0.61 | 0.0304 | -0.6988 | 0 | base_plus_full_lactate |
| 0.62 | 0.0289 | -0.7435 | 0 | base_plus_full_lactate |
| 0.63 | 0.026 | -0.7906 | 0 | base_plus_full_lactate |
| 0.64 | 0.0228 | -0.8404 | 0 | base_plus_full_lactate |
| 0.65 | 0.0262 | -0.8929 | 0 | base_plus_full_lactate |
| 0.66 | 0.022 | -0.9486 | 0 | base_plus_full_lactate |
| 0.67 | 0.0203 | -1.0077 | 0 | base_plus_full_lactate |
| 0.68 | 0.0157 | -1.0704 | 0 | base_plus_full_lactate |
| 0.69 | 0.0121 | -1.1372 | 0 | base_plus_full_lactate |
| 0.7 | 0.0089 | -1.2084 | 0 | base_plus_full_lactate |
| 0.71 | 0.0065 | -1.2846 | 0 | base_plus_full_lactate |
| 0.72 | 0.0037 | -1.3662 | 0 | base_plus_full_lactate |
| 0.73 | 0.0048 | -1.4538 | 0 | base_plus_full_lactate |
| 0.74 | 0.0024 | -1.5482 | 0 | base_plus_full_lactate |
| 0.75 | 0.002 | -1.6501 | 0 | base_plus_full_lactate |
| 0.76 | -8.27e-04 | -1.7605 | 0 | base_plus_full_lactate |
| 0.77 | 0.0022 | -1.8806 | 0 | base_plus_full_lactate |
| 0.78 | 5.87e-04 | -2.0115 | 0 | base_plus_full_lactate |
| 0.79 | -4.73e-04 | -2.1549 | 0 | base_plus_full_lactate |
| 0.8 | -0.004 | -2.3127 | 0 | base_plus_full_lactate |

Note. The first part provides binned calibration data by model: mean_predicted is the mean predicted probability within each bin, observed is the observed in-hospital mortality proportion, and n is the number of patients. The second part provides decision curve data by threshold probability and model: net_benefit is the model-based net benefit, and treat_all and treat_none are reference strategies. These data correspond to Figure 4A and Figure 4B, respectively. Source files: Supplementary_Table_S8_Calibration_and_decision_curve_source_data.xlsx.

**Supplementary Table S9. Variance inflation factors for the primary adjusted model**

| **variable** | **vif** |
| --- | --- |
| sofa | 1.927 |
| charlson_comorbidity_index | 1.609 |
| age | 1.54 |
| mechvent_24h | 1.374 |
| vasoactive_24h | 1.33 |
| creatinine_max | 1.327 |
| mbp_mean | 1.098 |

Note. Variance inflation factors were calculated for covariates in the primary MIMIC-IV adjusted logistic regression model. Higher VIF values indicate greater multicollinearity. VIF, variance inflation factor. Source files: Supplementary_Table_S9_Primary_model_VIF.csv.

**Supplementary Table S10. Prediction performance comparing foldwise trajectory group with simpler lactate summaries**

| **Model** | **AUROC** | **AUPRC** | **Brier score** | **Delta AUROC vs base** | **Delta AUPRC vs base** | **Net benefit at 0.30** | **Net benefit at 0.40** | **Net benefit at 0.50** |
| --- | --- | --- | --- | --- | --- | --- | --- | --- |
| Clinical base model | 0.703 | 0.532 | 0.199 | 0 | 0 | 0.125 | 0.062 | 0.026 |
| Clinical base + initial lactate | 0.719 | 0.548 | 0.195 | 0.016 | 0.016 | 0.134 | 0.077 | 0.032 |
| Clinical base + last lactate | 0.734 | 0.592 | 0.189 | 0.031 | 0.06 | 0.136 | 0.078 | 0.059 |
| Clinical base + initial and last lactate | 0.736 | 0.594 | 0.188 | 0.033 | 0.062 | 0.139 | 0.082 | 0.058 |
| Clinical base + initial lactate and clearance | 0.727 | 0.571 | 0.192 | 0.024 | 0.039 | 0.133 | 0.083 | 0.048 |
| Clinical base + observed 18-24 h lactate | 0.728 | 0.586 | 0.19 | 0.025 | 0.054 | 0.131 | 0.073 | 0.058 |
| Clinical base + mean observed lactate | 0.727 | 0.573 | 0.191 | 0.024 | 0.041 | 0.137 | 0.077 | 0.056 |
| Clinical base + time-weighted mean lactate | 0.729 | 0.576 | 0.191 | 0.026 | 0.044 | 0.135 | 0.078 | 0.057 |
| Clinical base + peak lactate | 0.721 | 0.566 | 0.193 | 0.018 | 0.034 | 0.137 | 0.077 | 0.043 |
| Clinical base + minimum lactate | 0.734 | 0.579 | 0.19 | 0.031 | 0.047 | 0.142 | 0.074 | 0.053 |
| Clinical base + lactate slope | 0.703 | 0.539 | 0.198 | 0 | 0.007 | 0.124 | 0.063 | 0.03 |
| Clinical base + persistent hyperlactatemia | 0.725 | 0.567 | 0.192 | 0.022 | 0.035 | 0.134 | 0.071 | 0.045 |
| Clinical base + trajectory group | 0.724 | 0.572 | 0.192 | 0.021 | 0.04 | 0.138 | 0.073 | 0.044 |
| Clinical base + full lactate dynamics | 0.735 | 0.588 | 0.189 | 0.032 | 0.055 | 0.136 | 0.08 | 0.051 |

Note. Models were evaluated using 5-fold stratified cross-validation among MIMIC-IV patients alive and still hospitalized at the 24-hour landmark. Delta values are absolute differences compared with the clinical base model. Comparators include initial, last, 18-24-hour, mean, time-weighted mean, peak and minimum lactate, slope, clearance, and persistent hyperlactatemia. The time-weighted mean used trapezoidal integration over 0-24 hours with the nearest observed value extended only to the interval boundaries. Persistent hyperlactatemia was defined as last lactate within 24 hours >=4 mmol/L. AUROC, area under the receiver operating characteristic curve; AUPRC, area under the precision-recall curve; Brier, Brier score. For every model containing trajectory group, lactate-window filling, scaling, K-means fitting, trajectory assignment, and model fitting were repeated within each training fold. Source files: Supplementary_Table_S10_Lactate_marker_model_performance.csv.

**Supplementary Table S11. Bootstrap increments comparing foldwise trajectory group with simpler lactate summaries**

| **Comparison** | **Delta AUROC (95% CI)** | **Delta AUPRC (95% CI)** | **Delta net benefit at 0.30** | **Delta net benefit at 0.40** | **Delta net benefit at 0.50** |
| --- | --- | --- | --- | --- | --- |
| Trajectory group vs initial lactate | 0.0050 (-0.0024 to 0.0130) | 0.0239 (0.0039 to 0.0412) | 0.0038 | -0.0048 | 0.0119 |
| Trajectory group vs last lactate | -0.0092 (-0.0170 to -0.0024) | -0.0200 (-0.0378 to -0.0016) | 0.0021 | -0.0056 | -0.0144 |
| Trajectory group vs initial and last lactate | -0.0120 (-0.0192 to -0.0056) | -0.0222 (-0.0385 to -0.0059) | -0.0013 | -0.0089 | -0.0134 |
| Trajectory group vs initial lactate and clearance | -0.0029 (-0.0103 to 0.0038) | 0.0012 (-0.0158 to 0.0195) | 0.0048 | -0.0108 | -0.004 |
| Trajectory group vs observed 18-24 h lactate | -0.0035 (-0.0113 to 0.0049) | -0.0142 (-0.0307 to 0.0032) | 0.0067 | -7.00e-04 | -0.0139 |
| Trajectory group vs mean observed lactate | -0.0028 (-0.0078 to 0.0019) | -0.0009 (-0.0158 to 0.0122) | 0.0011 | -0.0046 | -0.0119 |
| Trajectory group vs time-weighted mean lactate | -0.0044 (-0.0085 to -0.0003) | -0.0040 (-0.0154 to 0.0076) | 0.0025 | -0.0058 | -0.0124 |
| Trajectory group vs peak lactate | 0.0034 (-0.0022 to 0.0087) | 0.0056 (-0.0098 to 0.0178) | 0.0011 | -0.0045 | 0.0015 |
| Trajectory group vs minimum lactate | -0.0093 (-0.0156 to -0.0033) | -0.0070 (-0.0245 to 0.0087) | -0.0041 | -0.0013 | -0.0089 |
| Trajectory group vs lactate slope | 0.0211 (0.0101 to 0.0309) | 0.0332 (0.0116 to 0.0539) | 0.014 | 0.0093 | 0.0144 |
| Trajectory group vs persistent hyperlactatemia | -0.0007 (-0.0100 to 0.0088) | 0.0046 (-0.0126 to 0.0212) | 0.0033 | 0.0012 | -0.001 |

Note. Bootstrap estimates used 500 patient-level resamples of out-of-fold predictions from the 24-hour landmark risk set. Delta values are trajectory minus comparator. Confidence intervals are empirical 95% intervals conditional on the fitted cross-validation pipeline; the complete pipeline was not refitted within each bootstrap sample. Net-benefit differences are reported at thresholds of 0.30, 0.40, and 0.50. All trajectory-containing predictions were generated by the primary foldwise trajectory pipeline. Source files: Supplementary_Table_S11_Trajectory_increment_vs_simple_markers.csv.

**Supplementary Table S12. AMI-CS and non-AMI-CS subgroup mortality by trajectory group in the MIMIC-IV landmark risk set**

| **subgroup_definition** | **subgroup** | **n** | **deaths** | **mortality_pct** | **persistent_high_lactate_n** |
| --- | --- | --- | --- | --- | --- |
| AMI-CS by acute MI ICD | Yes | 798 | 292 | 36.5915 | 101 |
| AMI-CS by acute MI ICD | Yes: trajectory group 1 | 302 | 75 | 24.8344 | 0 |
| AMI-CS by acute MI ICD | Yes: trajectory group 2 | 307 | 101 | 32.899 | 18 |
| AMI-CS by acute MI ICD | Yes: trajectory group 3 | 146 | 82 | 56.1644 | 43 |
| AMI-CS by acute MI ICD | Yes: trajectory group 4 | 43 | 34 | 79.0698 | 40 |
| AMI-CS by acute MI ICD | No | 1217 | 388 | 31.8817 | 164 |
| AMI-CS by acute MI ICD | No: trajectory group 1 | 421 | 101 | 23.9905 | 2 |
| AMI-CS by acute MI ICD | No: trajectory group 2 | 467 | 128 | 27.409 | 10 |
| AMI-CS by acute MI ICD | No: trajectory group 3 | 254 | 107 | 42.126 | 80 |
| AMI-CS by acute MI ICD | No: trajectory group 4 | 75 | 52 | 69.3333 | 72 |
| MI history/Charlson marker | Yes | 958 | 347 | 36.2213 | 117 |
| MI history/Charlson marker | Yes: trajectory group 1 | 360 | 92 | 25.5556 | 0 |
| MI history/Charlson marker | Yes: trajectory group 2 | 369 | 119 | 32.2493 | 18 |
| MI history/Charlson marker | Yes: trajectory group 3 | 177 | 95 | 53.6723 | 50 |
| MI history/Charlson marker | Yes: trajectory group 4 | 52 | 41 | 78.8462 | 49 |
| MI history/Charlson marker | No | 1057 | 333 | 31.5043 | 148 |
| MI history/Charlson marker | No: trajectory group 1 | 363 | 84 | 23.1405 | 2 |
| MI history/Charlson marker | No: trajectory group 2 | 405 | 110 | 27.1605 | 10 |
| MI history/Charlson marker | No: trajectory group 3 | 223 | 94 | 42.1525 | 73 |
| MI history/Charlson marker | No: trajectory group 4 | 66 | 45 | 68.1818 | 63 |

Note. AMI-CS was defined using acute myocardial infarction diagnosis codes during the same hospital admission. Analyses were restricted to patients alive and still hospitalized at 24 hours. Trajectory groups follow the primary MIMIC-IV ordering from lowest to highest 24-hour lactate burden. Mortality denotes in-hospital mortality. AMI-CS, acute myocardial infarction-related cardiogenic shock. Source files: Supplementary_Table_S12_AMI_subgroup_mortality.csv.

**Supplementary Table S13. AMI-CS and non-AMI-CS subgroup adjusted trajectory ORs in the MIMIC-IV landmark risk set**

| **subgroup_definition** | **subgroup** | **n_complete** | **aic** | **term** | **or** | **ci95_low** | **ci95_high** | **p_value** |
| --- | --- | --- | --- | --- | --- | --- | --- | --- |
| AMI-CS by acute MI ICD | Yes | 797 | 924.826 | traj_2 | 1.2825 | 0.8823 | 1.8641 | 0.1923 |
| AMI-CS by acute MI ICD | Yes | 797 | 924.826 | traj_3 | 3.1019 | 1.9551 | 4.9214 | 1.53e-06 |
| AMI-CS by acute MI ICD | Yes | 797 | 924.826 | traj_4 | 8.1007 | 3.5185 | 18.6504 | 8.80e-07 |
| AMI-CS by acute MI ICD | No | 1216 | 1358.3268 | traj_2 | 1.041 | 0.7548 | 1.4359 | 0.8064 |
| AMI-CS by acute MI ICD | No | 1216 | 1358.3268 | traj_3 | 1.6115 | 1.1092 | 2.3413 | 0.0123 |
| AMI-CS by acute MI ICD | No | 1216 | 1358.3268 | traj_4 | 4.6595 | 2.5361 | 8.5608 | 7.10e-07 |
| MI history/Charlson marker | Yes | 957 | 1103.8923 | traj_2 | 1.196 | 0.8494 | 1.684 | 0.3054 |
| MI history/Charlson marker | Yes | 957 | 1103.8923 | traj_3 | 2.639 | 1.7316 | 4.022 | 6.37e-06 |
| MI history/Charlson marker | Yes | 957 | 1103.8923 | traj_4 | 7.2193 | 3.3654 | 15.4867 | 3.85e-07 |
| MI history/Charlson marker | No | 1056 | 1178.4095 | traj_2 | 1.0852 | 0.7661 | 1.5373 | 0.6452 |
| MI history/Charlson marker | No | 1056 | 1178.4095 | traj_3 | 1.6745 | 1.1222 | 2.4987 | 0.0116 |
| MI history/Charlson marker | No | 1056 | 1178.4095 | traj_4 | 4.6865 | 2.4723 | 8.8838 | 2.20e-06 |

Note. Adjusted logistic regression was performed separately within AMI-CS and non-AMI-CS subgroups. Group 1 was the reference trajectory group. Models adjusted for the same MIMIC-IV covariates used in the primary adjusted analysis where estimable. P values are two-sided. OR, odds ratio; CI, confidence interval; AIC, Akaike information criterion; AMI-CS, acute myocardial infarction-related cardiogenic shock. Source files: Supplementary_Table_S13_AMI_subgroup_adjusted_OR.csv.

**Supplementary Table S14. Persistent hyperlactatemia adjusted association in MIMIC-IV**

| **comparison** | **n_complete** | **or** | **ci95** | **p_value** |
| --- | --- | --- | --- | --- |
| Persistent hyperlactatemia vs no persistent hyperlactatemia | 2013 | 3.1893 | 2.36-4.31 | 3.64e-14 |

Note. Persistent hyperlactatemia was defined as last lactate within 24 hours >=4 mmol/L. The comparison group was patients without persistent hyperlactatemia. The model adjusted for age, SOFA score, Charlson comorbidity index, mean arterial pressure, creatinine, mechanical ventilation, and vasoactive agent use. P values are two-sided. OR, odds ratio; CI, confidence interval; SOFA, Sequential Organ Failure Assessment. Source files: Supplementary_Table_S14_Persistent_hyperlactatemia_adjusted_OR.csv.

**Supplementary Table S15. Early lactate measurement availability, 24-hour deaths, lactate testing frequency, and landmark risk-set construction**

| **analysis** | **group** | **n** | **hospital_deaths** | **hospital_mortality_pct** | **death_within_24h** | **death_within_24h_pct** | **sofa_median** | **sapsii_median** | **oasis_median** | **adjusted_or** | **ci95_low** | **ci95_high** | **p_value** |
| --- | --- | --- | --- | --- | --- | --- | --- | --- | --- | --- | --- | --- | --- |
| lactate_measurement_availability | 0 lactates | 331 | 125 | 37.7644 | 27 | 8.1571 |  |  |  |  |  |  |  |
| lactate_measurement_availability | 1 lactate | 410 | 145 | 35.3659 | 46 | 11.2195 |  |  |  |  |  |  |  |
| lactate_measurement_availability | >=2 lactates | 2181 | 844 | 38.6978 | 157 | 7.1985 | 8 | 47 | 37 |  |  |  |  |
| landmark_risk_set_accounting | not_hospitalized_at_24h | 166 | 164 | 98.7952 | 157 | 94.5783 |  |  |  |  |  |  |  |
| landmark_risk_set_accounting | alive_and_hospitalized_at_24h | 2015 | 680 | 33.7469 | 0 | 0 |  |  |  |  |  |  |  |
| 24h_landmark_alive_and_hospitalized | trajectory_group_1 | 723 | 176 | 24.343 | 0 | 0 |  |  |  |  |  |  |  |
| 24h_landmark_alive_and_hospitalized | trajectory_group_2 | 774 | 229 | 29.5866 | 0 | 0 |  |  |  |  |  |  |  |
| 24h_landmark_alive_and_hospitalized | trajectory_group_3 | 400 | 189 | 47.25 | 0 | 0 |  |  |  |  |  |  |  |
| 24h_landmark_alive_and_hospitalized | trajectory_group_4 | 118 | 86 | 72.8814 | 0 | 0 |  |  |  |  |  |  |  |
| 24h_landmark_adjusted_or | traj_2 | 2013 |  |  |  |  |  |  |  | 1.1314 | 0.8873 | 1.4426 | 0.3193 |
| 24h_landmark_adjusted_or | traj_3 | 2013 |  |  |  |  |  |  |  | 2.0789 | 1.5584 | 2.7732 | 6.44e-07 |
| 24h_landmark_adjusted_or | traj_4 | 2013 |  |  |  |  |  |  |  | 5.8137 | 3.5724 | 9.4609 | 1.39e-12 |
| eligible_lactate_frequency_and_severity | 2 lactates | 367 | 134 | 36.5123 |  |  | 7 | 43 | 34 |  |  |  |  |
| eligible_lactate_frequency_and_severity | 3-4 lactates | 719 | 256 | 35.605 |  |  | 7 | 44 | 35 |  |  |  |  |
| eligible_lactate_frequency_and_severity | >=5 lactates | 1095 | 454 | 41.4612 |  |  | 9 | 50 | 39 |  |  |  |  |

Note. This table reports early lactate measurement availability among all MIMIC-IV cardiogenic shock patients, deaths within 24 hours, illness severity by lactate testing frequency among trajectory-eligible patients, and a 24-hour landmark analysis restricted to patients alive and still hospitalized at 24 hours after ICU admission. Continued hospitalization was defined from hospital discharge time; recorded death time was summarized separately. Deaths within 24 hours required a recorded death time more than 0 and no more than 24 hours after ICU admission; two records with death times before ICU admission were excluded from that timing summary. Adjusted ORs use Group 1 as the reference group and the primary MIMIC-IV covariates where available. P values are two-sided. OR, odds ratio; CI, confidence interval; SOFA, Sequential Organ Failure Assessment; SAPS II, Simplified Acute Physiology Score II; OASIS, Oxford Acute Severity of Illness Score. Source files: Supplementary_Table_S15_24h_landmark_early_death.csv.

**Supplementary Table S16. Lactate-window observation and imputation audit**

| **cohort_or_group** | **time_window** | **fill_status** | **n_cells** | **pct_of_patients** | **pct_of_cells** | **metric** | **value** |
| --- | --- | --- | --- | --- | --- | --- | --- |
| MIMIC-IV overall | lact_0_6h | observed | 1995 | 91.4718 |  |  |  |
| MIMIC-IV overall | lact_0_6h | within_patient_edge_extrapolation | 186 | 8.5282 |  |  |  |
| MIMIC-IV overall | lact_12_18h | observed | 1500 | 68.7758 |  |  |  |
| MIMIC-IV overall | lact_12_18h | within_patient_edge_extrapolation | 401 | 18.3861 |  |  |  |
| MIMIC-IV overall | lact_12_18h | within_patient_linear_interpolation | 280 | 12.8381 |  |  |  |
| MIMIC-IV overall | lact_18_24h | observed | 1346 | 61.7148 |  |  |  |
| MIMIC-IV overall | lact_18_24h | within_patient_edge_extrapolation | 835 | 38.2852 |  |  |  |
| MIMIC-IV overall | lact_6_12h | observed | 1697 | 77.8083 |  |  |  |
| MIMIC-IV overall | lact_6_12h | within_patient_edge_extrapolation | 174 | 7.978 |  |  |  |
| MIMIC-IV overall | lact_6_12h | within_patient_linear_interpolation | 310 | 14.2137 |  |  |  |
| trajectory_group_1 | all_windows | observed | 1993 |  | 68.1601 |  |  |
| trajectory_group_1 | all_windows | within_patient_edge_extrapolation | 619 |  | 21.1696 |  |  |
| trajectory_group_1 | all_windows | within_patient_linear_interpolation | 312 |  | 10.6703 |  |  |
| trajectory_group_2 | all_windows | observed | 2391 |  | 76.0496 |  |  |
| trajectory_group_2 | all_windows | within_patient_edge_extrapolation | 533 |  | 16.9529 |  |  |
| trajectory_group_2 | all_windows | within_patient_linear_interpolation | 220 |  | 6.9975 |  |  |
| trajectory_group_3 | all_windows | observed | 1488 |  | 85.3211 |  |  |
| trajectory_group_3 | all_windows | within_patient_edge_extrapolation | 204 |  | 11.6972 |  |  |
| trajectory_group_3 | all_windows | within_patient_linear_interpolation | 52 |  | 2.9817 |  |  |
| trajectory_group_4 | all_windows | observed | 666 |  | 73.0263 |  |  |
| trajectory_group_4 | all_windows | within_patient_edge_extrapolation | 240 |  | 26.3158 |  |  |
| trajectory_group_4 | all_windows | within_patient_linear_interpolation | 6 |  | 0.6579 |  |  |
| trajectory_group_1 | observed_window_count | 1 observed windows | 43 |  | 5.8824 |  |  |
| trajectory_group_1 | observed_window_count | 2 observed windows | 279 |  | 38.1669 |  |  |
| trajectory_group_1 | observed_window_count | 3 observed windows | 244 |  | 33.3789 |  |  |
| trajectory_group_1 | observed_window_count | 4 observed windows | 165 |  | 22.5718 |  |  |
| trajectory_group_2 | observed_window_count | 1 observed windows | 38 |  | 4.8346 |  |  |
| trajectory_group_2 | observed_window_count | 2 observed windows | 190 |  | 24.173 |  |  |
| trajectory_group_2 | observed_window_count | 3 observed windows | 259 |  | 32.9517 |  |  |
| trajectory_group_2 | observed_window_count | 4 observed windows | 299 |  | 38.0407 |  |  |
| trajectory_group_3 | observed_window_count | 1 observed windows | 17 |  | 3.8991 |  |  |
| trajectory_group_3 | observed_window_count | 2 observed windows | 49 |  | 11.2385 |  |  |
| trajectory_group_3 | observed_window_count | 3 observed windows | 107 |  | 24.5413 |  |  |
| trajectory_group_3 | observed_window_count | 4 observed windows | 263 |  | 60.3211 |  |  |
| trajectory_group_4 | observed_window_count | 1 observed windows | 45 |  | 19.7368 |  |  |
| trajectory_group_4 | observed_window_count | 2 observed windows | 36 |  | 15.7895 |  |  |
| trajectory_group_4 | observed_window_count | 3 observed windows | 39 |  | 17.1053 |  |  |
| trajectory_group_4 | observed_window_count | 4 observed windows | 108 |  | 47.3684 |  |  |
| MIMIC-IV overall | last_measurement_to_24h_landmark | timing_audit | 2181 |  |  | last_measurement_gap_median_hours | 4.2833 |
| MIMIC-IV overall | last_measurement_to_24h_landmark | timing_audit | 2181 |  |  | last_measurement_gap_q25_hours | 1.8269 |
| MIMIC-IV overall | last_measurement_to_24h_landmark | timing_audit | 2181 |  |  | last_measurement_gap_q75_hours | 9.3275 |
| MIMIC-IV overall | last_measurement_to_24h_landmark | timing_audit | 2181 |  |  | last_measurement_within_3h_pct | 39.0188 |
| MIMIC-IV overall | last_measurement_to_24h_landmark | timing_audit | 2181 |  |  | last_measurement_within_6h_pct | 61.7148 |
| MIMIC-IV overall | last_measurement_to_24h_landmark | timing_audit | 2181 |  |  | last_measurement_more_than_12h_before_landmark_pct | 17.8817 |
| trajectory_group_1 | last_measurement_to_24h_landmark | timing_audit | 731 |  |  | last_measurement_gap_median_hours | 5.3308 |
| trajectory_group_1 | last_measurement_to_24h_landmark | timing_audit | 731 |  |  | last_measurement_gap_q25_hours | 2.1128 |
| trajectory_group_1 | last_measurement_to_24h_landmark | timing_audit | 731 |  |  | last_measurement_gap_q75_hours | 11.0538 |
| trajectory_group_1 | last_measurement_to_24h_landmark | timing_audit | 731 |  |  | last_measurement_within_3h_pct | 32.2845 |
| trajectory_group_1 | last_measurement_to_24h_landmark | timing_audit | 731 |  |  | last_measurement_within_6h_pct | 54.9932 |
| trajectory_group_1 | last_measurement_to_24h_landmark | timing_audit | 731 |  |  | last_measurement_more_than_12h_before_landmark_pct | 21.6142 |
| trajectory_group_2 | last_measurement_to_24h_landmark | timing_audit | 786 |  |  | last_measurement_gap_median_hours | 4.3788 |
| trajectory_group_2 | last_measurement_to_24h_landmark | timing_audit | 786 |  |  | last_measurement_gap_q25_hours | 2.0833 |
| trajectory_group_2 | last_measurement_to_24h_landmark | timing_audit | 786 |  |  | last_measurement_gap_q75_hours | 8.5958 |
| trajectory_group_2 | last_measurement_to_24h_landmark | timing_audit | 786 |  |  | last_measurement_within_3h_pct | 37.2774 |
| trajectory_group_2 | last_measurement_to_24h_landmark | timing_audit | 786 |  |  | last_measurement_within_6h_pct | 61.9593 |
| trajectory_group_2 | last_measurement_to_24h_landmark | timing_audit | 786 |  |  | last_measurement_more_than_12h_before_landmark_pct | 15.2672 |
| trajectory_group_3 | last_measurement_to_24h_landmark | timing_audit | 436 |  |  | last_measurement_gap_median_hours | 2.5496 |
| trajectory_group_3 | last_measurement_to_24h_landmark | timing_audit | 436 |  |  | last_measurement_gap_q25_hours | 1.1897 |
| trajectory_group_3 | last_measurement_to_24h_landmark | timing_audit | 436 |  |  | last_measurement_gap_q75_hours | 5.5542 |
| trajectory_group_3 | last_measurement_to_24h_landmark | timing_audit | 436 |  |  | last_measurement_within_3h_pct | 55.9633 |
| trajectory_group_3 | last_measurement_to_24h_landmark | timing_audit | 436 |  |  | last_measurement_within_6h_pct | 77.2936 |
| trajectory_group_3 | last_measurement_to_24h_landmark | timing_audit | 436 |  |  | last_measurement_more_than_12h_before_landmark_pct | 8.945 |
| trajectory_group_4 | last_measurement_to_24h_landmark | timing_audit | 228 |  |  | last_measurement_gap_median_hours | 5.7132 |
| trajectory_group_4 | last_measurement_to_24h_landmark | timing_audit | 228 |  |  | last_measurement_gap_q25_hours | 2.1992 |
| trajectory_group_4 | last_measurement_to_24h_landmark | timing_audit | 228 |  |  | last_measurement_gap_q75_hours | 13.7222 |
| trajectory_group_4 | last_measurement_to_24h_landmark | timing_audit | 228 |  |  | last_measurement_within_3h_pct | 34.2105 |
| trajectory_group_4 | last_measurement_to_24h_landmark | timing_audit | 228 |  |  | last_measurement_within_6h_pct | 52.6316 |
| trajectory_group_4 | last_measurement_to_24h_landmark | timing_audit | 228 |  |  | last_measurement_more_than_12h_before_landmark_pct | 32.0175 |
| MIMIC-IV overall | last_measurement_gap_stratum | 0-3 h | 851 | 39.0188 |  | in_hospital_mortality_pct | 39.2479 |
| MIMIC-IV overall | last_measurement_gap_stratum | >3-6 h | 495 | 22.696 |  | in_hospital_mortality_pct | 34.9495 |
| MIMIC-IV overall | last_measurement_gap_stratum | >6-12 h | 445 | 20.4035 |  | in_hospital_mortality_pct | 35.7303 |
| MIMIC-IV overall | last_measurement_gap_stratum | >12 h | 390 | 17.8817 |  | in_hospital_mortality_pct | 45.641 |
| MIMIC-IV overall | duplicate_timestamp_audit | same_timestamp_records | 10 |  |  | duplicate_timestamps | 5 |
| MIMIC-IV overall | duplicate_timestamp_audit | same_timestamp_discordant_values | 10 |  |  | discordant_duplicate_timestamps | 4 |

Note. Observed windows contain at least one measured lactate value in the corresponding 6-hour interval. Internal missing windows were filled by within-patient linear interpolation; edge windows were filled using the nearest observed within-patient value. Cohort median filling was reserved for any remaining missing windows after within-patient filling. Percentages are reported by patients for time-window rows and by all cells for trajectory-group rows. The timing audit reports the interval between the last measured lactate and the 24-hour landmark. Measurements sharing an identical timestamp were collapsed to their median; the duplicate-timestamp audit reports their frequency. Source files: Supplementary_Table_S16_Lactate_imputation_audit.csv.

**Supplementary Table S17. Cluster stability, observed-window, and landmark-only refitting sensitivity analyses**

| **analysis** | **metric** | **value** | **n** | **ci95_low** | **ci95_high** | **p_value** |
| --- | --- | --- | --- | --- | --- | --- |
| primary_k4 | silhouette | 0.3444 | 2181 |  |  |  |
| candidate_k | k_2_silhouette | 0.5479 | 2181 |  |  |  |
| candidate_k | k_3_silhouette | 0.4331 | 2181 |  |  |  |
| candidate_k | k_4_silhouette | 0.3444 | 2181 |  |  |  |
| candidate_k | k_5_silhouette | 0.3019 | 2181 |  |  |  |
| kmeans_seed_stability | adjusted_rand_index_vs_primary | 1 | 2181 |  |  |  |
| kmeans_seed_stability | adjusted_rand_index_vs_primary | 0.9969 | 2181 |  |  |  |
| kmeans_seed_stability | adjusted_rand_index_vs_primary | 0.9874 | 2181 |  |  |  |
| kmeans_seed_stability | adjusted_rand_index_vs_primary | 0.9676 | 2181 |  |  |  |
| kmeans_seed_stability | adjusted_rand_index_vs_primary | 0.9985 | 2181 |  |  |  |
| kmeans_seed_stability | adjusted_rand_index_vs_primary | 1 | 2181 |  |  |  |
| kmeans_seed_stability | adjusted_rand_index_vs_primary | 0.9761 | 2181 |  |  |  |
| kmeans_seed_stability | adjusted_rand_index_vs_primary | 0.9985 | 2181 |  |  |  |
| kmeans_seed_stability | adjusted_rand_index_vs_primary | 0.9761 | 2181 |  |  |  |
| kmeans_seed_stability | adjusted_rand_index_vs_primary | 0.9893 | 2181 |  |  |  |
| kmeans_seed_stability | adjusted_rand_index_vs_primary | 0.9706 | 2181 |  |  |  |
| kmeans_seed_stability | adjusted_rand_index_vs_primary | 0.9676 | 2181 |  |  |  |
| kmeans_seed_stability | adjusted_rand_index_vs_primary | 0.9969 | 2181 |  |  |  |
| kmeans_seed_stability | adjusted_rand_index_vs_primary | 0.9985 | 2181 |  |  |  |
| kmeans_seed_stability | adjusted_rand_index_vs_primary | 0.9893 | 2181 |  |  |  |
| kmeans_seed_stability | adjusted_rand_index_vs_primary | 0.9733 | 2181 |  |  |  |
| kmeans_seed_stability | adjusted_rand_index_vs_primary | 0.9706 | 2181 |  |  |  |
| kmeans_seed_stability | adjusted_rand_index_vs_primary | 0.9761 | 2181 |  |  |  |
| kmeans_seed_stability | adjusted_rand_index_vs_primary | 1 | 2181 |  |  |  |
| kmeans_seed_stability | adjusted_rand_index_vs_primary | 0.9893 | 2181 |  |  |  |
| kmeans_seed_stability | adjusted_rand_index_vs_primary | 0.9706 | 2181 |  |  |  |
| kmeans_seed_stability | adjusted_rand_index_vs_primary | 1 | 2181 |  |  |  |
| kmeans_seed_stability | adjusted_rand_index_vs_primary | 1 | 2181 |  |  |  |
| kmeans_seed_stability | adjusted_rand_index_vs_primary | 0.9978 | 2181 |  |  |  |
| kmeans_seed_stability | adjusted_rand_index_vs_primary | 1 | 2181 |  |  |  |
| kmeans_seed_stability | adjusted_rand_index_vs_primary | 0.9988 | 2181 |  |  |  |
| kmeans_seed_stability | adjusted_rand_index_vs_primary | 0.9761 | 2181 |  |  |  |
| kmeans_seed_stability | adjusted_rand_index_vs_primary | 0.9733 | 2181 |  |  |  |
| kmeans_seed_stability | adjusted_rand_index_vs_primary | 0.9904 | 2181 |  |  |  |
| kmeans_seed_stability | adjusted_rand_index_vs_primary | 0.9718 | 2181 |  |  |  |
| kmeans_seed_stability | adjusted_rand_index_vs_primary | 0.9733 | 2181 |  |  |  |
| kmeans_seed_stability | adjusted_rand_index_vs_primary | 0.9761 | 2181 |  |  |  |
| kmeans_seed_stability | adjusted_rand_index_vs_primary | 1 | 2181 |  |  |  |
| kmeans_seed_stability | adjusted_rand_index_vs_primary | 1 | 2181 |  |  |  |
| kmeans_seed_stability | adjusted_rand_index_vs_primary | 1 | 2181 |  |  |  |
| kmeans_seed_stability | adjusted_rand_index_vs_primary | 0.9978 | 2181 |  |  |  |
| kmeans_seed_stability | adjusted_rand_index_vs_primary | 0.9791 | 2181 |  |  |  |
| kmeans_seed_stability | adjusted_rand_index_vs_primary | 0.9706 | 2181 |  |  |  |
| kmeans_seed_stability | adjusted_rand_index_vs_primary | 0.9969 | 2181 |  |  |  |
| kmeans_seed_stability | adjusted_rand_index_vs_primary | 1 | 2181 |  |  |  |
| kmeans_seed_stability | adjusted_rand_index_vs_primary | 0.9706 | 2181 |  |  |  |
| kmeans_seed_stability | adjusted_rand_index_vs_primary | 0.9733 | 2181 |  |  |  |
| kmeans_seed_stability | adjusted_rand_index_vs_primary | 1 | 2181 |  |  |  |
| kmeans_seed_stability | adjusted_rand_index_vs_primary | 1 | 2181 |  |  |  |
| kmeans_seed_stability | adjusted_rand_index_vs_primary | 0.9837 | 2181 |  |  |  |
| kmeans_seed_stability | adjusted_rand_index_vs_primary | 0.988 | 2181 |  |  |  |
| kmeans_seed_stability | adjusted_rand_index_vs_primary | 0.9676 | 2181 |  |  |  |
| kmeans_seed_stability | adjusted_rand_index_vs_primary | 0.9837 | 2181 |  |  |  |
| kmeans_seed_stability | adjusted_rand_index_vs_primary | 0.9985 | 2181 |  |  |  |
| kmeans_seed_stability | adjusted_rand_index_vs_primary | 1 | 2181 |  |  |  |
| kmeans_seed_stability | adjusted_rand_index_vs_primary | 0.9761 | 2181 |  |  |  |
| kmeans_seed_stability | adjusted_rand_index_vs_primary | 0.9733 | 2181 |  |  |  |
| kmeans_seed_stability | adjusted_rand_index_vs_primary | 1 | 2181 |  |  |  |
| kmeans_seed_stability | adjusted_rand_index_vs_primary | 0.9969 | 2181 |  |  |  |
| kmeans_seed_stability | adjusted_rand_index_vs_primary | 1 | 2181 |  |  |  |
| kmeans_seed_stability | adjusted_rand_index_vs_primary | 0.9838 | 2181 |  |  |  |
| kmeans_seed_stability | adjusted_rand_index_vs_primary | 0.9965 | 2181 |  |  |  |
| kmeans_seed_stability | adjusted_rand_index_vs_primary | 0.9985 | 2181 |  |  |  |
| kmeans_seed_stability | adjusted_rand_index_vs_primary | 1 | 2181 |  |  |  |
| kmeans_seed_stability | adjusted_rand_index_vs_primary | 1 | 2181 |  |  |  |
| kmeans_seed_stability | adjusted_rand_index_vs_primary | 1 | 2181 |  |  |  |
| kmeans_seed_stability | adjusted_rand_index_vs_primary | 0.9837 | 2181 |  |  |  |
| kmeans_seed_stability | adjusted_rand_index_vs_primary | 1 | 2181 |  |  |  |
| kmeans_seed_stability | adjusted_rand_index_vs_primary | 1 | 2181 |  |  |  |
| kmeans_seed_stability | adjusted_rand_index_vs_primary | 0.9893 | 2181 |  |  |  |
| kmeans_seed_stability | adjusted_rand_index_vs_primary | 1 | 2181 |  |  |  |
| kmeans_seed_stability | adjusted_rand_index_vs_primary | 0.9969 | 2181 |  |  |  |
| kmeans_seed_stability | adjusted_rand_index_vs_primary | 1 | 2181 |  |  |  |
| kmeans_seed_stability | adjusted_rand_index_vs_primary | 0.9985 | 2181 |  |  |  |
| kmeans_seed_stability | adjusted_rand_index_vs_primary | 0.9985 | 2181 |  |  |  |
| kmeans_seed_stability | adjusted_rand_index_vs_primary | 1 | 2181 |  |  |  |
| kmeans_seed_stability | adjusted_rand_index_vs_primary | 0.9985 | 2181 |  |  |  |
| kmeans_seed_stability | adjusted_rand_index_vs_primary | 0.9676 | 2181 |  |  |  |
| kmeans_seed_stability | adjusted_rand_index_vs_primary | 0.9733 | 2181 |  |  |  |
| kmeans_seed_stability | adjusted_rand_index_vs_primary | 0.9985 | 2181 |  |  |  |
| kmeans_seed_stability | adjusted_rand_index_vs_primary | 1 | 2181 |  |  |  |
| kmeans_seed_stability | adjusted_rand_index_vs_primary | 1 | 2181 |  |  |  |
| kmeans_seed_stability | adjusted_rand_index_vs_primary | 0.9791 | 2181 |  |  |  |
| kmeans_seed_stability | adjusted_rand_index_vs_primary | 1 | 2181 |  |  |  |
| kmeans_seed_stability | adjusted_rand_index_vs_primary | 0.9985 | 2181 |  |  |  |
| kmeans_seed_stability | adjusted_rand_index_vs_primary | 0.9733 | 2181 |  |  |  |
| kmeans_seed_stability | adjusted_rand_index_vs_primary | 0.9761 | 2181 |  |  |  |
| kmeans_seed_stability | adjusted_rand_index_vs_primary | 1 | 2181 |  |  |  |
| kmeans_seed_stability | adjusted_rand_index_vs_primary | 0.9985 | 2181 |  |  |  |
| kmeans_seed_stability | adjusted_rand_index_vs_primary | 1 | 2181 |  |  |  |
| kmeans_seed_stability | adjusted_rand_index_vs_primary | 0.9706 | 2181 |  |  |  |
| kmeans_seed_stability | adjusted_rand_index_vs_primary | 1 | 2181 |  |  |  |
| kmeans_seed_stability | adjusted_rand_index_vs_primary | 0.9822 | 2181 |  |  |  |
| kmeans_seed_stability | adjusted_rand_index_vs_primary | 0.9985 | 2181 |  |  |  |
| kmeans_seed_stability | adjusted_rand_index_vs_primary | 1 | 2181 |  |  |  |
| kmeans_seed_stability | adjusted_rand_index_vs_primary | 0.9761 | 2181 |  |  |  |
| kmeans_seed_stability | adjusted_rand_index_vs_primary | 1 | 2181 |  |  |  |
| kmeans_seed_stability | adjusted_rand_index_vs_primary | 0.9706 | 2181 |  |  |  |
| kmeans_seed_stability | adjusted_rand_index_vs_primary | 0.9733 | 2181 |  |  |  |
| kmeans_seed_stability | adjusted_rand_index_vs_primary | 1 | 2181 |  |  |  |
| kmeans_seed_stability | adjusted_rand_index_vs_primary | 0.9893 | 2181 |  |  |  |
| kmeans_seed_stability | adjusted_rand_index_vs_primary | 0.9822 | 2181 |  |  |  |
| kmeans_seed_stability | adjusted_rand_index_vs_primary | 0.9985 | 2181 |  |  |  |
| kmeans_seed_stability | adjusted_rand_index_vs_primary | 0.9733 | 2181 |  |  |  |
| kmeans_seed_stability | adjusted_rand_index_vs_primary | 0.988 | 2181 |  |  |  |
| patient_bootstrap_stability | adjusted_rand_index_vs_primary | 0.9657 | 2181 |  |  |  |
| patient_bootstrap_stability | adjusted_rand_index_vs_primary | 0.9165 | 2181 |  |  |  |
| patient_bootstrap_stability | adjusted_rand_index_vs_primary | 0.9645 | 2181 |  |  |  |
| patient_bootstrap_stability | adjusted_rand_index_vs_primary | 0.8997 | 2181 |  |  |  |
| patient_bootstrap_stability | adjusted_rand_index_vs_primary | 0.9741 | 2181 |  |  |  |
| patient_bootstrap_stability | adjusted_rand_index_vs_primary | 0.8197 | 2181 |  |  |  |
| patient_bootstrap_stability | adjusted_rand_index_vs_primary | 0.9337 | 2181 |  |  |  |
| patient_bootstrap_stability | adjusted_rand_index_vs_primary | 0.9646 | 2181 |  |  |  |
| patient_bootstrap_stability | adjusted_rand_index_vs_primary | 0.9635 | 2181 |  |  |  |
| patient_bootstrap_stability | adjusted_rand_index_vs_primary | 0.7921 | 2181 |  |  |  |
| patient_bootstrap_stability | adjusted_rand_index_vs_primary | 0.9091 | 2181 |  |  |  |
| patient_bootstrap_stability | adjusted_rand_index_vs_primary | 0.8018 | 2181 |  |  |  |
| patient_bootstrap_stability | adjusted_rand_index_vs_primary | 0.907 | 2181 |  |  |  |
| patient_bootstrap_stability | adjusted_rand_index_vs_primary | 0.9359 | 2181 |  |  |  |
| patient_bootstrap_stability | adjusted_rand_index_vs_primary | 0.8924 | 2181 |  |  |  |
| patient_bootstrap_stability | adjusted_rand_index_vs_primary | 0.9258 | 2181 |  |  |  |
| patient_bootstrap_stability | adjusted_rand_index_vs_primary | 0.8699 | 2181 |  |  |  |
| patient_bootstrap_stability | adjusted_rand_index_vs_primary | 0.9057 | 2181 |  |  |  |
| patient_bootstrap_stability | adjusted_rand_index_vs_primary | 0.9842 | 2181 |  |  |  |
| patient_bootstrap_stability | adjusted_rand_index_vs_primary | 0.9307 | 2181 |  |  |  |
| patient_bootstrap_stability | adjusted_rand_index_vs_primary | 0.9843 | 2181 |  |  |  |
| patient_bootstrap_stability | adjusted_rand_index_vs_primary | 0.9677 | 2181 |  |  |  |
| patient_bootstrap_stability | adjusted_rand_index_vs_primary | 0.9001 | 2181 |  |  |  |
| patient_bootstrap_stability | adjusted_rand_index_vs_primary | 0.9483 | 2181 |  |  |  |
| patient_bootstrap_stability | adjusted_rand_index_vs_primary | 0.9187 | 2181 |  |  |  |
| patient_bootstrap_stability | adjusted_rand_index_vs_primary | 0.9225 | 2181 |  |  |  |
| patient_bootstrap_stability | adjusted_rand_index_vs_primary | 0.911 | 2181 |  |  |  |
| patient_bootstrap_stability | adjusted_rand_index_vs_primary | 0.8384 | 2181 |  |  |  |
| patient_bootstrap_stability | adjusted_rand_index_vs_primary | 0.9719 | 2181 |  |  |  |
| patient_bootstrap_stability | adjusted_rand_index_vs_primary | 0.8317 | 2181 |  |  |  |
| patient_bootstrap_stability | adjusted_rand_index_vs_primary | 0.9454 | 2181 |  |  |  |
| patient_bootstrap_stability | adjusted_rand_index_vs_primary | 0.9787 | 2181 |  |  |  |
| patient_bootstrap_stability | adjusted_rand_index_vs_primary | 0.8845 | 2181 |  |  |  |
| patient_bootstrap_stability | adjusted_rand_index_vs_primary | 0.9202 | 2181 |  |  |  |
| patient_bootstrap_stability | adjusted_rand_index_vs_primary | 0.9171 | 2181 |  |  |  |
| patient_bootstrap_stability | adjusted_rand_index_vs_primary | 0.9417 | 2181 |  |  |  |
| patient_bootstrap_stability | adjusted_rand_index_vs_primary | 0.9605 | 2181 |  |  |  |
| patient_bootstrap_stability | adjusted_rand_index_vs_primary | 0.9089 | 2181 |  |  |  |
| patient_bootstrap_stability | adjusted_rand_index_vs_primary | 0.9454 | 2181 |  |  |  |
| patient_bootstrap_stability | adjusted_rand_index_vs_primary | 0.8245 | 2181 |  |  |  |
| patient_bootstrap_stability | adjusted_rand_index_vs_primary | 0.9412 | 2181 |  |  |  |
| patient_bootstrap_stability | adjusted_rand_index_vs_primary | 0.9289 | 2181 |  |  |  |
| patient_bootstrap_stability | adjusted_rand_index_vs_primary | 0.9137 | 2181 |  |  |  |
| patient_bootstrap_stability | adjusted_rand_index_vs_primary | 0.8819 | 2181 |  |  |  |
| patient_bootstrap_stability | adjusted_rand_index_vs_primary | 0.9296 | 2181 |  |  |  |
| patient_bootstrap_stability | adjusted_rand_index_vs_primary | 0.9691 | 2181 |  |  |  |
| patient_bootstrap_stability | adjusted_rand_index_vs_primary | 0.9683 | 2181 |  |  |  |
| patient_bootstrap_stability | adjusted_rand_index_vs_primary | 0.918 | 2181 |  |  |  |
| patient_bootstrap_stability | adjusted_rand_index_vs_primary | 0.9772 | 2181 |  |  |  |
| patient_bootstrap_stability | adjusted_rand_index_vs_primary | 0.9566 | 2181 |  |  |  |
| patient_bootstrap_stability | adjusted_rand_index_vs_primary | 0.9717 | 2181 |  |  |  |
| patient_bootstrap_stability | adjusted_rand_index_vs_primary | 0.934 | 2181 |  |  |  |
| patient_bootstrap_stability | adjusted_rand_index_vs_primary | 0.9401 | 2181 |  |  |  |
| patient_bootstrap_stability | adjusted_rand_index_vs_primary | 0.9743 | 2181 |  |  |  |
| patient_bootstrap_stability | adjusted_rand_index_vs_primary | 0.9296 | 2181 |  |  |  |
| patient_bootstrap_stability | adjusted_rand_index_vs_primary | 0.9737 | 2181 |  |  |  |
| patient_bootstrap_stability | adjusted_rand_index_vs_primary | 0.9471 | 2181 |  |  |  |
| patient_bootstrap_stability | adjusted_rand_index_vs_primary | 0.983 | 2181 |  |  |  |
| patient_bootstrap_stability | adjusted_rand_index_vs_primary | 0.9512 | 2181 |  |  |  |
| patient_bootstrap_stability | adjusted_rand_index_vs_primary | 0.9095 | 2181 |  |  |  |
| patient_bootstrap_stability | adjusted_rand_index_vs_primary | 0.9553 | 2181 |  |  |  |
| patient_bootstrap_stability | adjusted_rand_index_vs_primary | 0.9699 | 2181 |  |  |  |
| patient_bootstrap_stability | adjusted_rand_index_vs_primary | 0.8898 | 2181 |  |  |  |
| patient_bootstrap_stability | adjusted_rand_index_vs_primary | 0.9318 | 2181 |  |  |  |
| patient_bootstrap_stability | adjusted_rand_index_vs_primary | 0.9381 | 2181 |  |  |  |
| patient_bootstrap_stability | adjusted_rand_index_vs_primary | 0.9044 | 2181 |  |  |  |
| patient_bootstrap_stability | adjusted_rand_index_vs_primary | 0.9162 | 2181 |  |  |  |
| patient_bootstrap_stability | adjusted_rand_index_vs_primary | 0.9686 | 2181 |  |  |  |
| patient_bootstrap_stability | adjusted_rand_index_vs_primary | 0.9746 | 2181 |  |  |  |
| patient_bootstrap_stability | adjusted_rand_index_vs_primary | 0.9111 | 2181 |  |  |  |
| patient_bootstrap_stability | adjusted_rand_index_vs_primary | 0.9455 | 2181 |  |  |  |
| patient_bootstrap_stability | adjusted_rand_index_vs_primary | 0.9731 | 2181 |  |  |  |
| patient_bootstrap_stability | adjusted_rand_index_vs_primary | 0.729 | 2181 |  |  |  |
| patient_bootstrap_stability | adjusted_rand_index_vs_primary | 0.9616 | 2181 |  |  |  |
| patient_bootstrap_stability | adjusted_rand_index_vs_primary | 0.9375 | 2181 |  |  |  |
| patient_bootstrap_stability | adjusted_rand_index_vs_primary | 0.9178 | 2181 |  |  |  |
| patient_bootstrap_stability | adjusted_rand_index_vs_primary | 0.7871 | 2181 |  |  |  |
| patient_bootstrap_stability | adjusted_rand_index_vs_primary | 0.9435 | 2181 |  |  |  |
| patient_bootstrap_stability | adjusted_rand_index_vs_primary | 0.9506 | 2181 |  |  |  |
| patient_bootstrap_stability | adjusted_rand_index_vs_primary | 0.9582 | 2181 |  |  |  |
| patient_bootstrap_stability | adjusted_rand_index_vs_primary | 0.9638 | 2181 |  |  |  |
| patient_bootstrap_stability | adjusted_rand_index_vs_primary | 0.982 | 2181 |  |  |  |
| patient_bootstrap_stability | adjusted_rand_index_vs_primary | 0.9425 | 2181 |  |  |  |
| patient_bootstrap_stability | adjusted_rand_index_vs_primary | 0.9489 | 2181 |  |  |  |
| patient_bootstrap_stability | adjusted_rand_index_vs_primary | 0.988 | 2181 |  |  |  |
| patient_bootstrap_stability | adjusted_rand_index_vs_primary | 0.9393 | 2181 |  |  |  |
| patient_bootstrap_stability | adjusted_rand_index_vs_primary | 0.9338 | 2181 |  |  |  |
| patient_bootstrap_stability | adjusted_rand_index_vs_primary | 0.9428 | 2181 |  |  |  |
| patient_bootstrap_stability | adjusted_rand_index_vs_primary | 0.9843 | 2181 |  |  |  |
| patient_bootstrap_stability | adjusted_rand_index_vs_primary | 0.9238 | 2181 |  |  |  |
| patient_bootstrap_stability | adjusted_rand_index_vs_primary | 0.9579 | 2181 |  |  |  |
| patient_bootstrap_stability | adjusted_rand_index_vs_primary | 0.9642 | 2181 |  |  |  |
| patient_bootstrap_stability | adjusted_rand_index_vs_primary | 0.9764 | 2181 |  |  |  |
| patient_bootstrap_stability | adjusted_rand_index_vs_primary | 0.9844 | 2181 |  |  |  |
| patient_bootstrap_stability | adjusted_rand_index_vs_primary | 0.8725 | 2181 |  |  |  |
| patient_bootstrap_stability | adjusted_rand_index_vs_primary | 0.9356 | 2181 |  |  |  |
| patient_bootstrap_stability | adjusted_rand_index_vs_primary | 0.9378 | 2181 |  |  |  |
| patient_bootstrap_stability | adjusted_rand_index_vs_primary | 0.9736 | 2181 |  |  |  |
| patient_bootstrap_stability | adjusted_rand_index_vs_primary | 0.9755 | 2181 |  |  |  |
| patient_bootstrap_stability | adjusted_rand_index_vs_primary | 0.9219 | 2181 |  |  |  |
| at_least_3_observed_windows | group_1_mortality_pct | 23.8612 | 461 |  |  |  |
| at_least_3_observed_windows | group_2_mortality_pct | 31.4176 | 522 |  |  |  |
| at_least_3_observed_windows | group_3_mortality_pct | 49.711 | 346 |  |  |  |
| at_least_3_observed_windows | group_4_mortality_pct | 79.3548 | 155 |  |  |  |
| at_least_3_observed_windows_adjusted_or | traj_2 | 1.2203 | 1482 | 0.9044 | 1.6467 | 0.1928 |
| at_least_3_observed_windows_adjusted_or | traj_3 | 2.3863 | 1482 | 1.7126 | 3.3251 | 2.77e-07 |
| at_least_3_observed_windows_adjusted_or | traj_4 | 8.7198 | 1482 | 5.3335 | 14.2561 | 5.91e-18 |
| at_least_3_observed_windows | silhouette | 0.3106 | 1484 |  |  |  |
| landmark_only_k4_refit | adjusted_rand_index_vs_full_cohort_labels | 0.8733 | 2015 |  |  |  |
| landmark_only_k4_refit | silhouette | 0.3316 | 2015 |  |  |  |
| landmark_only_k4_refit | group_1_mortality_pct | 24.3867 | 693 |  |  |  |
| landmark_only_k4_refit | group_2_mortality_pct | 29.8177 | 768 |  |  |  |
| landmark_only_k4_refit | group_3_mortality_pct | 43.8903 | 401 |  |  |  |
| landmark_only_k4_refit | group_4_mortality_pct | 69.281 | 153 |  |  |  |
| landmark_only_k4_refit_adjusted_or | traj_2 | 1.1244 | 2013 | 0.8788 | 1.4385 | 0.3511 |
| landmark_only_k4_refit_adjusted_or | traj_3 | 1.7726 | 2013 | 1.3268 | 2.3682 | 1.07e-04 |
| landmark_only_k4_refit_adjusted_or | traj_4 | 5.0166 | 2013 | 3.2558 | 7.7297 | 2.64e-13 |
| candidate_k_extended_metrics | k_2_silhouette | 0.5479 | 2181 |  |  |  |
| candidate_k_extended_metrics | k_2_calinski_harabasz | 3174.8522 | 2181 |  |  |  |
| candidate_k_extended_metrics | k_2_davies_bouldin | 0.7199 | 2181 |  |  |  |
| candidate_k_extended_metrics | k_3_silhouette | 0.4331 | 2181 |  |  |  |
| candidate_k_extended_metrics | k_3_calinski_harabasz | 2969.3046 | 2181 |  |  |  |
| candidate_k_extended_metrics | k_3_davies_bouldin | 0.8364 | 2181 |  |  |  |
| candidate_k_extended_metrics | k_4_silhouette | 0.3444 | 2181 |  |  |  |
| candidate_k_extended_metrics | k_4_calinski_harabasz | 2687.9127 | 2181 |  |  |  |
| candidate_k_extended_metrics | k_4_davies_bouldin | 0.9552 | 2181 |  |  |  |
| candidate_k_extended_metrics | k_5_silhouette | 0.3018 | 2181 |  |  |  |
| candidate_k_extended_metrics | k_5_calinski_harabasz | 2366.1616 | 2181 |  |  |  |
| candidate_k_extended_metrics | k_5_davies_bouldin | 1.0718 | 2181 |  |  |  |

Note. Candidate K=2 to K=5 solutions were assessed using silhouette, Calinski-Harabasz, and Davies-Bouldin indices. Random-seed and patient-bootstrap stability were quantified using the adjusted Rand index versus the primary K=4 solution across 100 repetitions. The observed-window sensitivity analysis restricted the cohort to patients with at least three actually observed lactate windows. K=4 was also refitted after restricting the data to the 24-hour landmark risk set. Group 1 is the reference group for adjusted ORs. OR, odds ratio; CI, confidence interval. Source files: Supplementary_Table_S17_Cluster_stability.csv.

**Supplementary Table S18. Prediction sensitivity analysis using globally assigned trajectory labels**

| **model** | **n** | **events** | **auroc** | **auprc** | **brier** | **delta_auroc_vs_clinical_base** | **delta_auprc_vs_clinical_base** |
| --- | --- | --- | --- | --- | --- | --- | --- |
| clinical_base | 2015 | 680 | 0.703 | 0.5323 | 0.1987 | 0 | 0 |
| base_plus_global_trajectory | 2015 | 680 | 0.7274 | 0.573 | 0.1916 | 0.0244 | 0.0406 |
| base_plus_full_lactate_global_trajectory | 2015 | 680 | 0.7367 | 0.5885 | 0.1889 | 0.0337 | 0.0562 |

Note. This sensitivity analysis assigned trajectory labels once using the full trajectory-analysis cohort before 5-fold prediction modeling. The primary prediction analysis instead repeated lactate-window filling, scaling, K-means fitting, trajectory assignment, and model fitting within each training fold. Delta values are absolute differences from the clinical base model. AUROC, area under the receiver operating characteristic curve; AUPRC, area under the precision-recall curve. Source files: Supplementary_Table_S18_Global_trajectory_label_sensitivity.csv.

**Supplementary Table S19. Adjustment-model and eICU-CRD missing-value sensitivity analyses**

| **cohort** | **model** | **covariates** | **term** | **n_complete** | **or** | **ci95_low** | **ci95_high** | **p_value** |
| --- | --- | --- | --- | --- | --- | --- | --- | --- |
| MIMIC-IV | primary | age; sofa; charlson_comorbidity_index; mbp_mean; creatinine_max; mechvent_24h; vasoactive_24h | traj_2 | 2013 | 1.1314 | 0.8873 | 1.4426 | 0.3193 |
| MIMIC-IV | primary | age; sofa; charlson_comorbidity_index; mbp_mean; creatinine_max; mechvent_24h; vasoactive_24h | traj_3 | 2013 | 2.0789 | 1.5584 | 2.7732 | 6.44e-07 |
| MIMIC-IV | primary | age; sofa; charlson_comorbidity_index; mbp_mean; creatinine_max; mechvent_24h; vasoactive_24h | traj_4 | 2013 | 5.8137 | 3.5724 | 9.4609 | 1.39e-12 |
| MIMIC-IV | without_treatment_variables | age; sofa; charlson_comorbidity_index; mbp_mean; creatinine_max | traj_2 | 2013 | 1.1477 | 0.9009 | 1.4622 | 0.2648 |
| MIMIC-IV | without_treatment_variables | age; sofa; charlson_comorbidity_index; mbp_mean; creatinine_max | traj_3 | 2013 | 2.1244 | 1.5947 | 2.8299 | 2.61e-07 |
| MIMIC-IV | without_treatment_variables | age; sofa; charlson_comorbidity_index; mbp_mean; creatinine_max | traj_4 | 2013 | 6.0551 | 3.7256 | 9.8411 | 3.66e-13 |
| MIMIC-IV | severity_only | age; sofa; charlson_comorbidity_index | traj_2 | 2015 | 1.1384 | 0.8941 | 1.4494 | 0.293 |
| MIMIC-IV | severity_only | age; sofa; charlson_comorbidity_index | traj_3 | 2015 | 2.0932 | 1.5742 | 2.7833 | 3.75e-07 |
| MIMIC-IV | severity_only | age; sofa; charlson_comorbidity_index | traj_4 | 2015 | 5.9862 | 3.6856 | 9.7229 | 4.79e-13 |
| eICU-CRD | primary_apache_without_acute_physiology | age; apachescore; meanbp; creatinine; vent; vasoactive_24h | traj_2 | 366 | 1.329 | 0.8318 | 2.1233 | 0.2342 |
| eICU-CRD | primary_apache_without_acute_physiology | age; apachescore; meanbp; creatinine; vent; vasoactive_24h | traj_3 | 366 | 1.9602 | 0.9681 | 3.969 | 0.0615 |
| eICU-CRD | primary_apache_without_acute_physiology | age; apachescore; meanbp; creatinine; vent; vasoactive_24h | traj_4 | 366 | 13.4139 | 3.3383 | 53.8996 | 2.53e-04 |
| eICU-CRD | acute_physiology_without_apache | age; acutephysiologyscore; meanbp; creatinine; vent; vasoactive_24h | traj_2 | 366 | 1.3422 | 0.8394 | 2.1462 | 0.2191 |
| eICU-CRD | acute_physiology_without_apache | age; acutephysiologyscore; meanbp; creatinine; vent; vasoactive_24h | traj_3 | 366 | 1.9886 | 0.9908 | 3.9914 | 0.0531 |
| eICU-CRD | acute_physiology_without_apache | age; acutephysiologyscore; meanbp; creatinine; vent; vasoactive_24h | traj_4 | 366 | 13.6974 | 3.4021 | 55.1475 | 2.31e-04 |
| eICU-CRD | apache_plus_acute_physiology_sensitivity | age; apachescore; acutephysiologyscore; meanbp; creatinine; vent; vasoactive_24h | traj_2 | 366 | 1.3243 | 0.83 | 2.1128 | 0.2386 |
| eICU-CRD | apache_plus_acute_physiology_sensitivity | age; apachescore; acutephysiologyscore; meanbp; creatinine; vent; vasoactive_24h | traj_3 | 366 | 1.9514 | 0.9546 | 3.9893 | 0.0669 |
| eICU-CRD | apache_plus_acute_physiology_sensitivity | age; apachescore; acutephysiologyscore; meanbp; creatinine; vent; vasoactive_24h | traj_4 | 366 | 13.3252 | 3.328 | 53.3534 | 2.54e-04 |
| eICU-CRD | without_treatment_variables | age; apachescore; meanbp; creatinine | traj_2 | 366 | 1.3288 | 0.8327 | 2.1204 | 0.2332 |
| eICU-CRD | without_treatment_variables | age; apachescore; meanbp; creatinine | traj_3 | 366 | 1.9476 | 0.9786 | 3.876 | 0.0577 |
| eICU-CRD | without_treatment_variables | age; apachescore; meanbp; creatinine | traj_4 | 366 | 13.3215 | 3.322 | 53.4198 | 2.58e-04 |
| eICU-CRD | median_imputation_with_missing_indicators | age; apachescore; meanbp; creatinine; vent; vasoactive_24h; apachescore_missing; meanbp_missing | traj_2 | 429 | 1.4195 | 0.8998 | 2.2394 | 0.1321 |
| eICU-CRD | median_imputation_with_missing_indicators | age; apachescore; meanbp; creatinine; vent; vasoactive_24h; apachescore_missing; meanbp_missing | traj_3 | 429 | 2.6363 | 1.382 | 5.029 | 0.0033 |
| eICU-CRD | median_imputation_with_missing_indicators | age; apachescore; meanbp; creatinine; vent; vasoactive_24h; apachescore_missing; meanbp_missing | traj_4 | 429 | 10.579 | 3.6952 | 30.2866 | 1.11e-05 |

Note. The primary eICU-CRD model used APACHE IVa score without acute physiology score and hospital-clustered standard errors. Sensitivity models examined treatment-variable adjustment and alternative severity-score specifications, including acute physiology score alone and both scores together. A median-imputation model with missingness indicators retained all landmark-eligible eICU-CRD patients. Group 1 is the reference trajectory group. P values are two-sided. OR, odds ratio; CI, confidence interval; APACHE, Acute Physiology and Chronic Health Evaluation IVa. Source files: Supplementary_Table_S19_Adjustment_model_sensitivity.csv.

**Supplementary Table S20. Model-specific missingness and complete-case sample sizes**

| **model** | **variable** | **n_total** | **n_missing** | **n_complete** | **missing_pct** |
| --- | --- | --- | --- | --- | --- |
| MIMIC-IV primary adjusted model | complete_case_model_n | 2015 | 2 | 2013 | 0.0993 |
| MIMIC-IV primary adjusted model | hospital_expire_flag | 2015 | 0 | 2015 | 0 |
| MIMIC-IV primary adjusted model | trajectory_group | 2015 | 0 | 2015 | 0 |
| MIMIC-IV primary adjusted model | age | 2015 | 0 | 2015 | 0 |
| MIMIC-IV primary adjusted model | sofa | 2015 | 0 | 2015 | 0 |
| MIMIC-IV primary adjusted model | charlson_comorbidity_index | 2015 | 0 | 2015 | 0 |
| MIMIC-IV primary adjusted model | mbp_mean | 2015 | 2 | 2013 | 0.0993 |
| MIMIC-IV primary adjusted model | creatinine_max | 2015 | 0 | 2015 | 0 |
| MIMIC-IV primary adjusted model | mechvent_24h | 2015 | 0 | 2015 | 0 |
| MIMIC-IV primary adjusted model | vasoactive_24h | 2015 | 0 | 2015 | 0 |
| MIMIC-IV no-treatment model | complete_case_model_n | 2015 | 2 | 2013 | 0.0993 |
| MIMIC-IV no-treatment model | hospital_expire_flag | 2015 | 0 | 2015 | 0 |
| MIMIC-IV no-treatment model | trajectory_group | 2015 | 0 | 2015 | 0 |
| MIMIC-IV no-treatment model | age | 2015 | 0 | 2015 | 0 |
| MIMIC-IV no-treatment model | sofa | 2015 | 0 | 2015 | 0 |
| MIMIC-IV no-treatment model | charlson_comorbidity_index | 2015 | 0 | 2015 | 0 |
| MIMIC-IV no-treatment model | mbp_mean | 2015 | 2 | 2013 | 0.0993 |
| MIMIC-IV no-treatment model | creatinine_max | 2015 | 0 | 2015 | 0 |
| eICU-CRD primary adjusted model | complete_case_model_n | 429 | 63 | 366 | 14.6853 |
| eICU-CRD primary adjusted model | hospital_expire_flag | 429 | 0 | 429 | 0 |
| eICU-CRD primary adjusted model | trajectory_group | 429 | 0 | 429 | 0 |
| eICU-CRD primary adjusted model | age | 429 | 0 | 429 | 0 |
| eICU-CRD primary adjusted model | apachescore | 429 | 63 | 366 | 14.6853 |
| eICU-CRD primary adjusted model | meanbp | 429 | 23 | 406 | 5.3613 |
| eICU-CRD primary adjusted model | creatinine | 429 | 23 | 406 | 5.3613 |
| eICU-CRD primary adjusted model | vent | 429 | 23 | 406 | 5.3613 |
| eICU-CRD primary adjusted model | vasoactive_24h | 429 | 0 | 429 | 0 |

Note. Complete-case sample sizes and variable-level missingness are reported for the primary and sensitivity adjusted models. n_complete denotes the number of patients with non-missing values for each variable; complete_case_model_n reports the final model sample size. Source files: Supplementary_Table_S20_Model_missingness_complete_cases.csv.

**Supplementary Table S21. Transportability of the persistent-hyperlactatemia association in eICU-CRD at the 24-hour landmark.**

| **analysis** | **group_or_comparison** | **n** | **deaths** | **mortality_pct** | **initial_lactate_median** | **last_lactate_median** | **adjusted_or** | **ci95_low** | **ci95_high** | **p_value** |
| --- | --- | --- | --- | --- | --- | --- | --- | --- | --- | --- |
| eICU 24-hour landmark persistent hyperlactatemia mortality | no persistent hyperlactatemia | 302 | 105 | 34.77 | 2.9 | 1.8 |  |  |  |  |
| eICU 24-hour landmark persistent hyperlactatemia mortality | persistent hyperlactatemia | 127 | 81 | 63.78 | 6.9 | 6.5 |  |  |  |  |
| eICU 24-hour landmark APACHE IVa-adjusted association | persistent hyperlactatemia vs no persistent hyperlactatemia | 366 |  |  |  |  | 2.36 | 1.3 | 4.3 | 0.0049 |

Note. Persistent hyperlactatemia was defined as last lactate within 24 hours >=4 mmol/L. The adjusted association used patients without persistent hyperlactatemia as the reference group, adjusted for APACHE IVa score and clinical covariates, and used hospital-clustered standard errors. P values are two-sided. OR, odds ratio; CI, confidence interval; APACHE IVa, Acute Physiology and Chronic Health Evaluation IVa. Source files: Supplementary_Table_S21_eICU_persistent_hyperlactatemia.csv.

**Supplementary Table S22. Full-cohort trajectory associations without landmark restriction**

| **cohort** | **trajectory_group** | **n** | **deaths** | **mortality_pct** | **adjusted_or** | **ci95_low** | **ci95_high** | **p_value** | **model_n** |
| --- | --- | --- | --- | --- | --- | --- | --- | --- | --- |
| MIMIC-IV | 1 | 731 | 184 | 25.171 | 1 |  |  |  | 2177 |
| MIMIC-IV | 2 | 786 | 240 | 30.5344 | 1.1396 | 0.8967 | 1.4483 | 0.2854 | 2177 |
| MIMIC-IV | 3 | 436 | 225 | 51.6055 | 2.365 | 1.7866 | 3.1306 | 1.79e-09 | 2177 |
| MIMIC-IV | 4 | 228 | 195 | 85.5263 | 11.611 | 7.4621 | 18.0669 | 1.61e-27 | 2177 |
| eICU-CRD | 1 | 156 | 44 | 28.2051 | 1 |  |  |  | 408 |
| eICU-CRD | 2 | 147 | 58 | 39.4558 | 1.4125 | 0.8973 | 2.2234 | 0.1357 | 408 |
| eICU-CRD | 3 | 107 | 65 | 60.7477 | 2.3939 | 1.2488 | 4.5892 | 0.0086 | 408 |
| eICU-CRD | 4 | 66 | 59 | 89.3939 | 20.9918 | 5.3981 | 81.6314 | 1.12e-05 | 408 |

Note. These sensitivity analyses include all trajectory-eligible patients without requiring survival and continued hospitalization to 24 hours. MIMIC-IV models used conventional standard errors; eICU-CRD models used hospital-clustered standard errors. Group 1 is the reference group. Source files: Supplementary_Table_S22_Full_cohort_associations.csv.

**Supplementary Table S23. Selection comparison by repeated lactate testing eligibility**

| **cohort** | **testing_group** | **metric** | **n_nonmissing** | **value** | **standardized_mean_difference_repeated_vs_not** | **median** | **q25** | **q75** | **events** |
| --- | --- | --- | --- | --- | --- | --- | --- | --- | --- |
| MIMIC-IV | at least two lactate measurements | population_n | 2181 | 2181 |  |  |  |  |  |
| MIMIC-IV | fewer than two lactate measurements | population_n | 741 | 741 |  |  |  |  |  |
| MIMIC-IV | at least two lactate measurements | age_years | 2181 | 67.0995 | -0.1831 | 69 | 59 | 77 |  |
| MIMIC-IV | fewer than two lactate measurements | age_years | 741 | 69.7139 | -0.1831 | 71 | 62 | 81 |  |
| MIMIC-IV | at least two lactate measurements | female | 2181 | 38.4227 | -0.1406 |  |  |  | 838 |
| MIMIC-IV | fewer than two lactate measurements | female | 741 | 45.3441 | -0.1406 |  |  |  | 336 |
| MIMIC-IV | at least two lactate measurements | in_hospital_mortality | 2181 | 38.6978 | 0.0467 |  |  |  | 844 |
| MIMIC-IV | fewer than two lactate measurements | in_hospital_mortality | 741 | 36.4372 | 0.0467 |  |  |  | 270 |
| MIMIC-IV | at least two lactate measurements | death_or_discharge_within_24h | 2181 | 7.6112 | -0.0973 |  |  |  | 166 |
| MIMIC-IV | fewer than two lactate measurements | death_or_discharge_within_24h | 741 | 10.3914 | -0.0973 |  |  |  | 77 |
| MIMIC-IV | at least two lactate measurements | death_within_24h | 2181 | 7.1985 | -0.0951 |  |  |  | 157 |
| MIMIC-IV | fewer than two lactate measurements | death_within_24h | 741 | 9.8516 | -0.0951 |  |  |  | 73 |
| eICU-CRD | at least two lactate measurements | population_n | 476 | 476 |  |  |  |  |  |
| eICU-CRD | fewer than two lactate measurements | population_n | 1174 | 1174 |  |  |  |  |  |
| eICU-CRD | at least two lactate measurements | age_years | 476 | 65.7038 | -0.0527 | 68 | 57 | 76 |  |
| eICU-CRD | fewer than two lactate measurements | age_years | 1174 | 66.4744 | -0.0527 | 68 | 57 | 77 |  |
| eICU-CRD | at least two lactate measurements | female | 476 | 42.0168 | 0.1194 |  |  |  | 200 |
| eICU-CRD | fewer than two lactate measurements | female | 1174 | 36.201 | 0.1194 |  |  |  | 425 |
| eICU-CRD | at least two lactate measurements | in_hospital_mortality | 476 | 47.479 | 0.331 |  |  |  | 226 |
| eICU-CRD | fewer than two lactate measurements | in_hospital_mortality | 1174 | 31.5162 | 0.331 |  |  |  | 370 |
| eICU-CRD | at least two lactate measurements | death_or_discharge_within_24h | 476 | 9.8739 | -0.084 |  |  |  | 47 |
| eICU-CRD | fewer than two lactate measurements | death_or_discharge_within_24h | 1174 | 12.5213 | -0.084 |  |  |  | 147 |
| eICU-CRD | at least two lactate measurements | death_within_24h | 476 | 8.4034 | -0.0281 |  |  |  | 40 |
| eICU-CRD | fewer than two lactate measurements | death_within_24h | 1174 | 9.1993 | -0.0281 |  |  |  | 108 |

Note. Patients with at least two lactate measurements within 24 hours were compared with patients having fewer than two measurements in each source cohort. Continuous age is reported using the mean, median, and interquartile range; binary variables are reported as events and percentages. Standardized mean differences compare the repeated-testing group with the group having fewer measurements. For MIMIC-IV, deaths within 24 hours required a recorded death time more than 0 and no more than 24 hours after ICU admission; two records with death times before ICU admission were excluded from that timing summary. Severity scores were not available from the current extraction for patients outside the analytic trajectory cohorts. Source files: Supplementary_Table_S23_Selection_comparison.csv.

**Supplementary Table S24. Lactate-window observation and filling status by trajectory group**

| **cohort_or_group** | **trajectory_group** | **time_window** | **fill_status** | **n_cells** | **denominator_cells** | **pct_of_group_window_cells** |
| --- | --- | --- | --- | --- | --- | --- |
| MIMIC-IV overall | all | lact_0_6h | observed | 1995 | 2181 | 91.4718 |
| MIMIC-IV overall | all | lact_0_6h | within_patient_edge_extrapolation | 186 | 2181 | 8.5282 |
| MIMIC-IV overall | all | lact_6_12h | observed | 1697 | 2181 | 77.8083 |
| MIMIC-IV overall | all | lact_6_12h | within_patient_edge_extrapolation | 174 | 2181 | 7.978 |
| MIMIC-IV overall | all | lact_6_12h | within_patient_linear_interpolation | 310 | 2181 | 14.2137 |
| MIMIC-IV overall | all | lact_12_18h | observed | 1500 | 2181 | 68.7758 |
| MIMIC-IV overall | all | lact_12_18h | within_patient_edge_extrapolation | 401 | 2181 | 18.3861 |
| MIMIC-IV overall | all | lact_12_18h | within_patient_linear_interpolation | 280 | 2181 | 12.8381 |
| MIMIC-IV overall | all | lact_18_24h | observed | 1346 | 2181 | 61.7148 |
| MIMIC-IV overall | all | lact_18_24h | within_patient_edge_extrapolation | 835 | 2181 | 38.2852 |
| MIMIC-IV group 3 | 3 | lact_0_6h | observed | 396 | 436 | 90.8257 |
| MIMIC-IV group 3 | 3 | lact_0_6h | within_patient_edge_extrapolation | 40 | 436 | 9.1743 |
| MIMIC-IV group 3 | 3 | lact_6_12h | observed | 385 | 436 | 88.3028 |
| MIMIC-IV group 3 | 3 | lact_6_12h | within_patient_edge_extrapolation | 24 | 436 | 5.5046 |
| MIMIC-IV group 3 | 3 | lact_6_12h | within_patient_linear_interpolation | 27 | 436 | 6.1927 |
| MIMIC-IV group 3 | 3 | lact_12_18h | observed | 370 | 436 | 84.8624 |
| MIMIC-IV group 3 | 3 | lact_12_18h | within_patient_edge_extrapolation | 41 | 436 | 9.4037 |
| MIMIC-IV group 3 | 3 | lact_12_18h | within_patient_linear_interpolation | 25 | 436 | 5.7339 |
| MIMIC-IV group 3 | 3 | lact_18_24h | observed | 337 | 436 | 77.2936 |
| MIMIC-IV group 3 | 3 | lact_18_24h | within_patient_edge_extrapolation | 99 | 436 | 22.7064 |
| MIMIC-IV group 2 | 2 | lact_0_6h | observed | 725 | 786 | 92.2392 |
| MIMIC-IV group 2 | 2 | lact_0_6h | within_patient_edge_extrapolation | 61 | 786 | 7.7608 |
| MIMIC-IV group 2 | 2 | lact_6_12h | observed | 631 | 786 | 80.2799 |
| MIMIC-IV group 2 | 2 | lact_6_12h | within_patient_edge_extrapolation | 49 | 786 | 6.2341 |
| MIMIC-IV group 2 | 2 | lact_6_12h | within_patient_linear_interpolation | 106 | 786 | 13.486 |
| MIMIC-IV group 2 | 2 | lact_12_18h | observed | 548 | 786 | 69.7201 |
| MIMIC-IV group 2 | 2 | lact_12_18h | within_patient_edge_extrapolation | 124 | 786 | 15.7761 |
| MIMIC-IV group 2 | 2 | lact_12_18h | within_patient_linear_interpolation | 114 | 786 | 14.5038 |
| MIMIC-IV group 2 | 2 | lact_18_24h | observed | 487 | 786 | 61.9593 |
| MIMIC-IV group 2 | 2 | lact_18_24h | within_patient_edge_extrapolation | 299 | 786 | 38.0407 |
| MIMIC-IV group 1 | 1 | lact_0_6h | observed | 658 | 731 | 90.0137 |
| MIMIC-IV group 1 | 1 | lact_0_6h | within_patient_edge_extrapolation | 73 | 731 | 9.9863 |
| MIMIC-IV group 1 | 1 | lact_6_12h | observed | 502 | 731 | 68.6731 |
| MIMIC-IV group 1 | 1 | lact_6_12h | within_patient_edge_extrapolation | 56 | 731 | 7.6607 |
| MIMIC-IV group 1 | 1 | lact_6_12h | within_patient_linear_interpolation | 173 | 731 | 23.6662 |
| MIMIC-IV group 1 | 1 | lact_12_18h | observed | 431 | 731 | 58.9603 |
| MIMIC-IV group 1 | 1 | lact_12_18h | within_patient_edge_extrapolation | 161 | 731 | 22.0246 |
| MIMIC-IV group 1 | 1 | lact_12_18h | within_patient_linear_interpolation | 139 | 731 | 19.015 |
| MIMIC-IV group 1 | 1 | lact_18_24h | observed | 402 | 731 | 54.9932 |
| MIMIC-IV group 1 | 1 | lact_18_24h | within_patient_edge_extrapolation | 329 | 731 | 45.0068 |
| MIMIC-IV group 4 | 4 | lact_0_6h | observed | 216 | 228 | 94.7368 |
| MIMIC-IV group 4 | 4 | lact_0_6h | within_patient_edge_extrapolation | 12 | 228 | 5.2632 |
| MIMIC-IV group 4 | 4 | lact_6_12h | observed | 179 | 228 | 78.5088 |
| MIMIC-IV group 4 | 4 | lact_6_12h | within_patient_edge_extrapolation | 45 | 228 | 19.7368 |
| MIMIC-IV group 4 | 4 | lact_6_12h | within_patient_linear_interpolation | 4 | 228 | 1.7544 |
| MIMIC-IV group 4 | 4 | lact_12_18h | observed | 151 | 228 | 66.2281 |
| MIMIC-IV group 4 | 4 | lact_12_18h | within_patient_edge_extrapolation | 75 | 228 | 32.8947 |
| MIMIC-IV group 4 | 4 | lact_12_18h | within_patient_linear_interpolation | 2 | 228 | 0.8772 |
| MIMIC-IV group 4 | 4 | lact_18_24h | observed | 120 | 228 | 52.6316 |
| MIMIC-IV group 4 | 4 | lact_18_24h | within_patient_edge_extrapolation | 108 | 228 | 47.3684 |

Note. Each patient contributes one cell per 6-hour window. Observed denotes a measured lactate in that window; linear interpolation denotes an internal missing window bounded by observed patient values; edge extrapolation carries the nearest within-patient value into an unobserved edge window. Percentages use the number of patients in the corresponding cohort or trajectory group as the denominator for each time window. Source files: Supplementary_Table_S24_Fill_status_by_group_and_window.csv.

**Supplementary Table S25. Centroid separation and trajectory-assignment clarity**

| **section** | **cohort** | **trajectory_group** | **metric** | **value** | **n** |
| --- | --- | --- | --- | --- | --- |
| centroid_pairwise_distance | MIMIC-IV derivation | 1 vs 2 | euclidean_distance_in_standardized_log_lactate_space | 1.3592 | 2181 |
| centroid_pairwise_distance | MIMIC-IV derivation | 1 vs 3 | euclidean_distance_in_standardized_log_lactate_space | 3.1559 | 2181 |
| centroid_pairwise_distance | MIMIC-IV derivation | 1 vs 4 | euclidean_distance_in_standardized_log_lactate_space | 5.7288 | 2181 |
| centroid_pairwise_distance | MIMIC-IV derivation | 2 vs 3 | euclidean_distance_in_standardized_log_lactate_space | 1.8083 | 2181 |
| centroid_pairwise_distance | MIMIC-IV derivation | 2 vs 4 | euclidean_distance_in_standardized_log_lactate_space | 4.4017 | 2181 |
| centroid_pairwise_distance | MIMIC-IV derivation | 3 vs 4 | euclidean_distance_in_standardized_log_lactate_space | 2.6227 | 2181 |
| model_summary | MIMIC-IV derivation | all | silhouette_score | 0.3444 | 2181 |
| model_summary | MIMIC-IV derivation | all | adjusted_rand_index_vs_saved_primary_labels | 1 | 2181 |
| model_summary | MIMIC-IV derivation | all | derivation_95th_percentile_nearest_distance_threshold | 1.6849 | 2181 |
| assignment_distance_summary | MIMIC-IV derivation | all | nearest_distance_median | 0.7048 | 2181 |
| assignment_distance_summary | MIMIC-IV derivation | all | nearest_distance_q25 | 0.4807 | 2181 |
| assignment_distance_summary | MIMIC-IV derivation | all | nearest_distance_q75 | 1.0217 | 2181 |
| assignment_distance_summary | MIMIC-IV derivation | all | nearest_distance_p95 | 1.6849 | 2181 |
| assignment_distance_summary | MIMIC-IV derivation | all | distance_margin_median | 0.7701 | 2181 |
| assignment_distance_summary | MIMIC-IV derivation | all | relative_margin_median | 0.518 | 2181 |
| assignment_distance_summary | MIMIC-IV derivation | all | nearest_to_second_ratio_median | 0.482 | 2181 |
| assignment_distance_summary | MIMIC-IV derivation | all | outside_derivation_95pct_distance_pct | 4.9977 | 2181 |
| assignment_distance_summary | MIMIC-IV derivation | 1 | nearest_distance_median | 0.5462 | 731 |
| assignment_distance_summary | MIMIC-IV derivation | 1 | nearest_distance_q25 | 0.3563 | 731 |
| assignment_distance_summary | MIMIC-IV derivation | 1 | nearest_distance_q75 | 0.7278 | 731 |
| assignment_distance_summary | MIMIC-IV derivation | 1 | nearest_distance_p95 | 1.1206 | 731 |
| assignment_distance_summary | MIMIC-IV derivation | 1 | distance_margin_median | 0.9696 | 731 |
| assignment_distance_summary | MIMIC-IV derivation | 1 | relative_margin_median | 0.6146 | 731 |
| assignment_distance_summary | MIMIC-IV derivation | 1 | nearest_to_second_ratio_median | 0.3854 | 731 |
| assignment_distance_summary | MIMIC-IV derivation | 1 | outside_derivation_95pct_distance_pct | 0 | 731 |
| assignment_distance_summary | MIMIC-IV derivation | 2 | nearest_distance_median | 0.6879 | 786 |
| assignment_distance_summary | MIMIC-IV derivation | 2 | nearest_distance_q25 | 0.4752 | 786 |
| assignment_distance_summary | MIMIC-IV derivation | 2 | nearest_distance_q75 | 0.9682 | 786 |
| assignment_distance_summary | MIMIC-IV derivation | 2 | nearest_distance_p95 | 1.515 | 786 |
| assignment_distance_summary | MIMIC-IV derivation | 2 | distance_margin_median | 0.578 | 786 |
| assignment_distance_summary | MIMIC-IV derivation | 2 | relative_margin_median | 0.4272 | 786 |
| assignment_distance_summary | MIMIC-IV derivation | 2 | nearest_to_second_ratio_median | 0.5728 | 786 |
| assignment_distance_summary | MIMIC-IV derivation | 2 | outside_derivation_95pct_distance_pct | 2.2901 | 786 |
| assignment_distance_summary | MIMIC-IV derivation | 3 | nearest_distance_median | 0.9486 | 436 |
| assignment_distance_summary | MIMIC-IV derivation | 3 | nearest_distance_q25 | 0.7331 | 436 |
| assignment_distance_summary | MIMIC-IV derivation | 3 | nearest_distance_q75 | 1.2558 | 436 |
| assignment_distance_summary | MIMIC-IV derivation | 3 | nearest_distance_p95 | 1.9615 | 436 |
| assignment_distance_summary | MIMIC-IV derivation | 3 | distance_margin_median | 0.8337 | 436 |
| assignment_distance_summary | MIMIC-IV derivation | 3 | relative_margin_median | 0.4492 | 436 |
| assignment_distance_summary | MIMIC-IV derivation | 3 | nearest_to_second_ratio_median | 0.5508 | 436 |
| assignment_distance_summary | MIMIC-IV derivation | 3 | outside_derivation_95pct_distance_pct | 9.1743 | 436 |
| assignment_distance_summary | MIMIC-IV derivation | 4 | nearest_distance_median | 1.1737 | 228 |
| assignment_distance_summary | MIMIC-IV derivation | 4 | nearest_distance_q25 | 0.7655 | 228 |
| assignment_distance_summary | MIMIC-IV derivation | 4 | nearest_distance_q75 | 1.6377 | 228 |
| assignment_distance_summary | MIMIC-IV derivation | 4 | nearest_distance_p95 | 2.4967 | 228 |
| assignment_distance_summary | MIMIC-IV derivation | 4 | distance_margin_median | 1.7025 | 228 |
| assignment_distance_summary | MIMIC-IV derivation | 4 | relative_margin_median | 0.5841 | 228 |
| assignment_distance_summary | MIMIC-IV derivation | 4 | nearest_to_second_ratio_median | 0.4159 | 228 |
| assignment_distance_summary | MIMIC-IV derivation | 4 | outside_derivation_95pct_distance_pct | 22.3684 | 228 |
| assignment_distance_summary | eICU-CRD fixed-centroid validation | all | nearest_distance_median | 0.7894 | 476 |
| assignment_distance_summary | eICU-CRD fixed-centroid validation | all | nearest_distance_q25 | 0.5342 | 476 |
| assignment_distance_summary | eICU-CRD fixed-centroid validation | all | nearest_distance_q75 | 1.2135 | 476 |
| assignment_distance_summary | eICU-CRD fixed-centroid validation | all | nearest_distance_p95 | 1.8699 | 476 |
| assignment_distance_summary | eICU-CRD fixed-centroid validation | all | distance_margin_median | 0.7807 | 476 |
| assignment_distance_summary | eICU-CRD fixed-centroid validation | all | relative_margin_median | 0.4939 | 476 |
| assignment_distance_summary | eICU-CRD fixed-centroid validation | all | nearest_to_second_ratio_median | 0.5061 | 476 |
| assignment_distance_summary | eICU-CRD fixed-centroid validation | all | outside_derivation_95pct_distance_pct | 7.7731 | 476 |
| assignment_distance_summary | eICU-CRD fixed-centroid validation | 1 | nearest_distance_median | 0.5746 | 103 |
| assignment_distance_summary | eICU-CRD fixed-centroid validation | 1 | nearest_distance_q25 | 0.3441 | 103 |
| assignment_distance_summary | eICU-CRD fixed-centroid validation | 1 | nearest_distance_q75 | 0.7436 | 103 |
| assignment_distance_summary | eICU-CRD fixed-centroid validation | 1 | nearest_distance_p95 | 1.3217 | 103 |
| assignment_distance_summary | eICU-CRD fixed-centroid validation | 1 | distance_margin_median | 0.9567 | 103 |
| assignment_distance_summary | eICU-CRD fixed-centroid validation | 1 | relative_margin_median | 0.6438 | 103 |
| assignment_distance_summary | eICU-CRD fixed-centroid validation | 1 | nearest_to_second_ratio_median | 0.3562 | 103 |
| assignment_distance_summary | eICU-CRD fixed-centroid validation | 1 | outside_derivation_95pct_distance_pct | 0 | 103 |
| assignment_distance_summary | eICU-CRD fixed-centroid validation | 2 | nearest_distance_median | 0.7055 | 136 |
| assignment_distance_summary | eICU-CRD fixed-centroid validation | 2 | nearest_distance_q25 | 0.5098 | 136 |
| assignment_distance_summary | eICU-CRD fixed-centroid validation | 2 | nearest_distance_q75 | 0.8916 | 136 |
| assignment_distance_summary | eICU-CRD fixed-centroid validation | 2 | nearest_distance_p95 | 1.5609 | 136 |
| assignment_distance_summary | eICU-CRD fixed-centroid validation | 2 | distance_margin_median | 0.576 | 136 |
| assignment_distance_summary | eICU-CRD fixed-centroid validation | 2 | relative_margin_median | 0.4252 | 136 |
| assignment_distance_summary | eICU-CRD fixed-centroid validation | 2 | nearest_to_second_ratio_median | 0.5748 | 136 |
| assignment_distance_summary | eICU-CRD fixed-centroid validation | 2 | outside_derivation_95pct_distance_pct | 3.6765 | 136 |
| assignment_distance_summary | eICU-CRD fixed-centroid validation | 3 | nearest_distance_median | 1.0907 | 143 |
| assignment_distance_summary | eICU-CRD fixed-centroid validation | 3 | nearest_distance_q25 | 0.7562 | 143 |
| assignment_distance_summary | eICU-CRD fixed-centroid validation | 3 | nearest_distance_q75 | 1.4733 | 143 |
| assignment_distance_summary | eICU-CRD fixed-centroid validation | 3 | nearest_distance_p95 | 2.0342 | 143 |
| assignment_distance_summary | eICU-CRD fixed-centroid validation | 3 | distance_margin_median | 0.6536 | 143 |
| assignment_distance_summary | eICU-CRD fixed-centroid validation | 3 | relative_margin_median | 0.3476 | 143 |
| assignment_distance_summary | eICU-CRD fixed-centroid validation | 3 | nearest_to_second_ratio_median | 0.6524 | 143 |
| assignment_distance_summary | eICU-CRD fixed-centroid validation | 3 | outside_derivation_95pct_distance_pct | 12.5874 | 143 |
| assignment_distance_summary | eICU-CRD fixed-centroid validation | 4 | nearest_distance_median | 1.0837 | 94 |
| assignment_distance_summary | eICU-CRD fixed-centroid validation | 4 | nearest_distance_q25 | 0.6948 | 94 |
| assignment_distance_summary | eICU-CRD fixed-centroid validation | 4 | nearest_distance_q75 | 1.4321 | 94 |
| assignment_distance_summary | eICU-CRD fixed-centroid validation | 4 | nearest_distance_p95 | 2.6765 | 94 |
| assignment_distance_summary | eICU-CRD fixed-centroid validation | 4 | distance_margin_median | 1.7795 | 94 |
| assignment_distance_summary | eICU-CRD fixed-centroid validation | 4 | relative_margin_median | 0.6062 | 94 |
| assignment_distance_summary | eICU-CRD fixed-centroid validation | 4 | nearest_to_second_ratio_median | 0.3938 | 94 |
| assignment_distance_summary | eICU-CRD fixed-centroid validation | 4 | outside_derivation_95pct_distance_pct | 14.8936 | 94 |
| external_assignment_concordance | eICU-CRD | all | adjusted_rand_index_reclustered_vs_fixed | 0.4468 | 476 |
| external_assignment_concordance | eICU-CRD | all | exact_ordered_group_agreement_pct | 69.5378 | 476 |
| external_assignment_concordance | eICU-CRD | reclustered_1_fixed_1 | patient_count | 103 | 476 |
| external_assignment_concordance | eICU-CRD | reclustered_1_fixed_2 | patient_count | 53 | 476 |
| external_assignment_concordance | eICU-CRD | reclustered_1_fixed_3 | patient_count | 0 | 476 |
| external_assignment_concordance | eICU-CRD | reclustered_1_fixed_4 | patient_count | 0 | 476 |
| external_assignment_concordance | eICU-CRD | reclustered_2_fixed_1 | patient_count | 0 | 476 |
| external_assignment_concordance | eICU-CRD | reclustered_2_fixed_2 | patient_count | 83 | 476 |
| external_assignment_concordance | eICU-CRD | reclustered_2_fixed_3 | patient_count | 64 | 476 |
| external_assignment_concordance | eICU-CRD | reclustered_2_fixed_4 | patient_count | 0 | 476 |
| external_assignment_concordance | eICU-CRD | reclustered_3_fixed_1 | patient_count | 0 | 476 |
| external_assignment_concordance | eICU-CRD | reclustered_3_fixed_2 | patient_count | 0 | 476 |
| external_assignment_concordance | eICU-CRD | reclustered_3_fixed_3 | patient_count | 79 | 476 |
| external_assignment_concordance | eICU-CRD | reclustered_3_fixed_4 | patient_count | 28 | 476 |
| external_assignment_concordance | eICU-CRD | reclustered_4_fixed_1 | patient_count | 0 | 476 |
| external_assignment_concordance | eICU-CRD | reclustered_4_fixed_2 | patient_count | 0 | 476 |
| external_assignment_concordance | eICU-CRD | reclustered_4_fixed_3 | patient_count | 0 | 476 |
| external_assignment_concordance | eICU-CRD | reclustered_4_fixed_4 | patient_count | 66 | 476 |

Note. Centroid and patient distances are Euclidean distances in standardized log-transformed lactate-window space. The distance margin is the second-nearest minus nearest centroid distance; the relative margin divides this difference by the second-nearest distance. For fixed-centroid eICU-CRD transportability, outside derivation distance support denotes a nearest-centroid distance above the MIMIC-IV 95th percentile. This is a distributional diagnostic and not an unclassifiable-patient threshold. The external concordance section reports the cross-tabulation, exact ordered-group agreement, and adjusted Rand index between eICU re-clustering and fixed MIMIC-IV-centroid assignment. Source files: Supplementary_Table_S25_Cluster_assignment_clarity.csv.

**Supplementary Table S26. Sensitivity analysis restricted to four directly observed lactate windows**

| **analysis_population** | **metric** | **trajectory_group** | **n** | **value** | **deaths** | **mortality_pct** | **adjusted_or** | **ci95_low** | **ci95_high** | **p_value** | **model_n** |
| --- | --- | --- | --- | --- | --- | --- | --- | --- | --- | --- | --- |
| all four windows directly observed | silhouette_score | all | 835 | 0.2899 |  |  |  |  |  |  |  |
| all four windows directly observed | adjusted_rand_index_vs_primary_group | all | 835 | 0.7578 |  |  |  |  |  |  |  |
| full trajectory cohort | group_result | 1 | 209 |  | 53 | 25.3589 | 1 |  |  |  | 835 |
| full trajectory cohort | group_result | 2 | 285 |  | 89 | 31.2281 | 1.1305 | 0.7397 | 1.7277 | 0.5708 | 835 |
| full trajectory cohort | group_result | 3 | 231 |  | 119 | 51.5152 | 2.6285 | 1.6766 | 4.1208 | 2.52e-05 | 835 |
| full trajectory cohort | group_result | 4 | 110 |  | 89 | 80.9091 | 10.5573 | 5.5892 | 19.9412 | 3.78e-13 | 835 |
| 24-hour landmark cohort | group_result | 1 | 208 |  | 52 | 25 | 1 |  |  |  | 818 |
| 24-hour landmark cohort | group_result | 2 | 281 |  | 85 | 30.2491 | 1.1133 | 0.7255 | 1.7084 | 0.6233 | 818 |
| 24-hour landmark cohort | group_result | 3 | 230 |  | 118 | 51.3043 | 2.6657 | 1.696 | 4.1898 | 2.14e-05 | 818 |
| 24-hour landmark cohort | group_result | 4 | 99 |  | 78 | 78.7879 | 9.4554 | 4.9715 | 17.9834 | 7.42e-12 | 818 |

Note. This analysis included only patients with a measured lactate value in each of the four 6-hour windows and therefore required no interpolation or edge extrapolation. K-means was refitted in this restricted sample. Adjusted logistic models used the primary MIMIC-IV covariates. Group 1 is the reference group. OR, odds ratio; CI, confidence interval. Source files: Supplementary_Table_S26_Complete_observed_windows.csv.

**Supplementary Table S27. Adjusted absolute mortality, risk differences, risk ratios, and odds ratios**

| **Cohort** | **Group** | **N at landmark** | **Deaths** | **Model included, n** | **Excluded for missing covariates, n** | **Adjusted mortality, % (95% CI)** | **Risk difference per 100 (95% CI)** | **Adjusted RR (95% CI)** | **Adjusted OR (95% CI)** |
| --- | --- | --- | --- | --- | --- | --- | --- | --- | --- |
| MIMIC-IV | Group 1 | 723 | 176 | 721 | 2 | 27.6 (24.4-31.1) | Reference | Reference | Reference |
| MIMIC-IV | Group 2 | 774 | 229 | 774 | 0 | 29.9 (26.9-33.1) | 2.3 (-2.2-6.8) | 1.10 (0.94-1.30) | 1.13 (0.89-1.44) |
| MIMIC-IV | Group 3 | 400 | 189 | 400 | 0 | 42.4 (37.8-47.2) | 14.8 (8.7-20.6) | 1.56 (1.31-1.85) | 2.08 (1.56-2.77) |
| MIMIC-IV | Group 4 | 118 | 86 | 118 | 0 | 64.8 (55.3-73.3) | 37.3 (26.7-46.4) | 2.15 (1.77-2.61) | 5.81 (3.57-9.46) |
| eICU-CRD fixed MIMIC-IV centroids | Group 1 | 102 | 26 | 79 | 23 | 32.1 (22.2-43.9) | Reference | Reference | Reference |
| eICU-CRD fixed MIMIC-IV centroids | Group 2 | 134 | 51 | 115 | 19 | 42.8 (34.0-51.6) | 10.7 (-3.3-23.2) | 1.40 (0.93-2.13) | 1.65 (0.87-3.12) |
| eICU-CRD fixed MIMIC-IV centroids | Group 3 | 129 | 59 | 113 | 16 | 41.7 (32.8-51.7) | 9.5 (-4.8-23.5) | 1.41 (0.91-2.18) | 1.57 (0.78-3.17) |
| eICU-CRD fixed MIMIC-IV centroids | Group 4 | 64 | 50 | 59 | 5 | 75.5 (58.0-87.6) | 43.4 (22.1-59.1) | 2.30 (1.47-3.62) | 7.71 (2.76-21.57) |

Note. Adjusted mortality is the marginal probability standardized to each cohort's complete-case covariate distribution. Confidence intervals for adjusted probabilities and risk differences were obtained from 5,000 coefficient draws using the fitted logistic-model covariance matrix. Risk ratios were estimated with modified Poisson regression using robust standard errors in MIMIC-IV and hospital-clustered standard errors in eICU-CRD. Group 1 is the reference group. RR, risk ratio; OR, odds ratio; CI, confidence interval. Source files: Supplementary_Table_S27_Adjusted_absolute_effects.csv.

**Supplementary Table S28. Repeated 5-fold cross-validation with complete foldwise trajectory fitting**

**Across-split summary**

| **model** | **metric** | **n_repeats** | **mean** | **sd** | **ci95_low_across_splits** | **ci95_high_across_splits** | **minimum** | **maximum** |
| --- | --- | --- | --- | --- | --- | --- | --- | --- |
| clinical_base | auroc | 20 | 0.7051 | 0.0015 | 0.7026 | 0.7076 | 0.7025 | 0.7078 |
| clinical_base | auprc | 20 | 0.5335 | 0.003 | 0.528 | 0.5373 | 0.526 | 0.5377 |
| clinical_base | brier | 20 | 0.1983 | 3.93e-04 | 0.1976 | 0.1989 | 0.1975 | 0.199 |
| clinical_base | calibration_intercept | 20 | -0.023 | 0.0062 | -0.031 | -0.0128 | -0.031 | -0.0123 |
| clinical_base | calibration_slope | 20 | 0.9614 | 0.0103 | 0.9452 | 0.978 | 0.9438 | 0.9786 |
| base_plus_initial_and_clearance | auroc | 20 | 0.7294 | 0.0014 | 0.7265 | 0.7311 | 0.7263 | 0.7313 |
| base_plus_initial_and_clearance | auprc | 20 | 0.5706 | 0.0027 | 0.5651 | 0.5738 | 0.5649 | 0.574 |
| base_plus_initial_and_clearance | brier | 20 | 0.1913 | 4.08e-04 | 0.1907 | 0.192 | 0.1907 | 0.1921 |
| base_plus_initial_and_clearance | calibration_intercept | 20 | -0.0265 | 0.0048 | -0.0337 | -0.018 | -0.0337 | -0.0178 |
| base_plus_initial_and_clearance | calibration_slope | 20 | 0.9562 | 0.0094 | 0.94 | 0.9701 | 0.937 | 0.9725 |
| base_plus_trajectory | auroc | 20 | 0.7252 | 0.0022 | 0.7198 | 0.7276 | 0.7181 | 0.7278 |
| base_plus_trajectory | auprc | 20 | 0.571 | 0.0031 | 0.5654 | 0.576 | 0.5625 | 0.5763 |
| base_plus_trajectory | brier | 20 | 0.1922 | 6.16e-04 | 0.1913 | 0.1935 | 0.1912 | 0.1942 |
| base_plus_trajectory | calibration_intercept | 20 | -0.0254 | 0.0086 | -0.048 | -0.0183 | -0.0533 | -0.0183 |
| base_plus_trajectory | calibration_slope | 20 | 0.9582 | 0.0132 | 0.9247 | 0.973 | 0.9144 | 0.9765 |
| base_plus_full_lactate_dynamics | auroc | 20 | 0.7363 | 0.0021 | 0.7313 | 0.7384 | 0.7297 | 0.7384 |
| base_plus_full_lactate_dynamics | auprc | 20 | 0.5865 | 0.0031 | 0.5803 | 0.5907 | 0.5775 | 0.5915 |
| base_plus_full_lactate_dynamics | brier | 20 | 0.1892 | 6.18e-04 | 0.1883 | 0.1904 | 0.1881 | 0.1911 |
| base_plus_full_lactate_dynamics | calibration_intercept | 20 | -0.0478 | 0.0095 | -0.0685 | -0.0359 | -0.074 | -0.0338 |
| base_plus_full_lactate_dynamics | calibration_slope | 20 | 0.9242 | 0.0145 | 0.8932 | 0.9438 | 0.8825 | 0.9475 |
| trajectory_minus_initial_and_clearance | auroc | 20 | -0.0042 | 0.0018 | -0.0084 | -0.0029 | -0.0107 | -0.0028 |
| trajectory_minus_initial_and_clearance | auprc | 20 | 4.32e-04 | 0.0033 | -0.0048 | 0.0058 | -0.0052 | 0.0063 |
| trajectory_minus_initial_and_clearance | brier | 20 | 9.30e-04 | 5.34e-04 | 2.93e-04 | 0.0021 | 2.81e-04 | 0.0024 |
| trajectory_minus_initial_and_clearance | calibration_intercept | 20 | 0.0011 | 0.0081 | -0.0167 | 0.0096 | -0.0217 | 0.0096 |
| trajectory_minus_initial_and_clearance | calibration_slope | 20 | 0.002 | 0.0117 | -0.021 | 0.0151 | -0.0319 | 0.0151 |

**Per-repeat results**

| **repeat** | **split_seed** | **model** | **n** | **events** | **auroc** | **auprc** | **brier** | **calibration_intercept** | **calibration_slope** |
| --- | --- | --- | --- | --- | --- | --- | --- | --- | --- |
| 1 | 2026 | clinical_base | 2015 | 680 | 0.703 | 0.5323 | 0.1987 | -0.0307 | 0.9504 |
| 1 | 2026 | base_plus_initial_and_clearance | 2015 | 680 | 0.7273 | 0.5709 | 0.1915 | -0.0317 | 0.9481 |
| 1 | 2026 | base_plus_trajectory | 2015 | 680 | 0.7241 | 0.5719 | 0.1922 | -0.0221 | 0.9604 |
| 1 | 2026 | base_plus_full_lactate_dynamics | 2015 | 680 | 0.7353 | 0.5878 | 0.1891 | -0.0506 | 0.9225 |
| 1 | 2026 | trajectory_minus_initial_and_clearance | 2015 | 680 | -0.0031 | 0.001 | 6.85e-04 | 0.0096 | 0.0123 |
| 2 | 2027 | clinical_base | 2015 | 680 | 0.704 | 0.5326 | 0.1985 | -0.0235 | 0.959 |
| 2 | 2027 | base_plus_initial_and_clearance | 2015 | 680 | 0.7288 | 0.5713 | 0.1913 | -0.0261 | 0.9554 |
| 2 | 2027 | base_plus_trajectory | 2015 | 680 | 0.7255 | 0.5718 | 0.1919 | -0.0236 | 0.9604 |
| 2 | 2027 | base_plus_full_lactate_dynamics | 2015 | 680 | 0.7362 | 0.5852 | 0.1891 | -0.0474 | 0.9214 |
| 2 | 2027 | trajectory_minus_initial_and_clearance | 2015 | 680 | -0.0033 | 4.86e-04 | 5.89e-04 | 0.0026 | 0.005 |
| 3 | 2028 | clinical_base | 2015 | 680 | 0.7049 | 0.5308 | 0.1984 | -0.0246 | 0.9585 |
| 3 | 2028 | base_plus_initial_and_clearance | 2015 | 680 | 0.7291 | 0.5698 | 0.1912 | -0.0284 | 0.9561 |
| 3 | 2028 | base_plus_trajectory | 2015 | 680 | 0.725 | 0.5705 | 0.1924 | -0.0251 | 0.9567 |
| 3 | 2028 | base_plus_full_lactate_dynamics | 2015 | 680 | 0.7355 | 0.5838 | 0.1894 | -0.0625 | 0.9051 |
| 3 | 2028 | trajectory_minus_initial_and_clearance | 2015 | 680 | -0.004 | 6.25e-04 | 0.0011 | 0.0032 | 5.69e-04 |
| 4 | 2029 | clinical_base | 2015 | 680 | 0.7046 | 0.5335 | 0.1982 | -0.022 | 0.9627 |
| 4 | 2029 | base_plus_initial_and_clearance | 2015 | 680 | 0.7299 | 0.574 | 0.1909 | -0.0238 | 0.9628 |
| 4 | 2029 | base_plus_trajectory | 2015 | 680 | 0.7252 | 0.5705 | 0.1924 | -0.0286 | 0.9547 |
| 4 | 2029 | base_plus_full_lactate_dynamics | 2015 | 680 | 0.7381 | 0.5892 | 0.1885 | -0.0404 | 0.9397 |
| 4 | 2029 | trajectory_minus_initial_and_clearance | 2015 | 680 | -0.0047 | -0.0034 | 0.0015 | -0.0048 | -0.0081 |
| 5 | 2030 | clinical_base | 2015 | 680 | 0.7046 | 0.5346 | 0.1983 | -0.025 | 0.9608 |
| 5 | 2030 | base_plus_initial_and_clearance | 2015 | 680 | 0.7291 | 0.5694 | 0.1914 | -0.0294 | 0.9551 |
| 5 | 2030 | base_plus_trajectory | 2015 | 680 | 0.726 | 0.5719 | 0.1919 | -0.0233 | 0.9643 |
| 5 | 2030 | base_plus_full_lactate_dynamics | 2015 | 680 | 0.7377 | 0.5897 | 0.1886 | -0.0382 | 0.9377 |
| 5 | 2030 | trajectory_minus_initial_and_clearance | 2015 | 680 | -0.0031 | 0.0025 | 5.17e-04 | 0.0061 | 0.0091 |
| 6 | 2031 | clinical_base | 2015 | 680 | 0.7057 | 0.5337 | 0.1982 | -0.0262 | 0.9626 |
| 6 | 2031 | base_plus_initial_and_clearance | 2015 | 680 | 0.7306 | 0.5734 | 0.1909 | -0.0231 | 0.9674 |
| 6 | 2031 | base_plus_trajectory | 2015 | 680 | 0.7246 | 0.5691 | 0.1925 | -0.0277 | 0.9584 |
| 6 | 2031 | base_plus_full_lactate_dynamics | 2015 | 680 | 0.7371 | 0.5892 | 0.1889 | -0.0435 | 0.9343 |
| 6 | 2031 | trajectory_minus_initial_and_clearance | 2015 | 680 | -0.006 | -0.0044 | 0.0016 | -0.0046 | -0.009 |
| 7 | 2032 | clinical_base | 2015 | 680 | 0.705 | 0.5306 | 0.1985 | -0.0307 | 0.9526 |
| 7 | 2032 | base_plus_initial_and_clearance | 2015 | 680 | 0.7296 | 0.5649 | 0.1917 | -0.0337 | 0.9469 |
| 7 | 2032 | base_plus_trajectory | 2015 | 680 | 0.7269 | 0.5703 | 0.192 | -0.0251 | 0.9606 |
| 7 | 2032 | base_plus_full_lactate_dynamics | 2015 | 680 | 0.737 | 0.5833 | 0.1895 | -0.0573 | 0.9158 |
| 7 | 2032 | trajectory_minus_initial_and_clearance | 2015 | 680 | -0.0028 | 0.0053 | 3.05e-04 | 0.0086 | 0.0137 |
| 8 | 2033 | clinical_base | 2015 | 680 | 0.7027 | 0.535 | 0.1987 | -0.031 | 0.9467 |
| 8 | 2033 | base_plus_initial_and_clearance | 2015 | 680 | 0.7263 | 0.5725 | 0.1917 | -0.0309 | 0.9433 |
| 8 | 2033 | base_plus_trajectory | 2015 | 680 | 0.7217 | 0.5693 | 0.1929 | -0.0421 | 0.9361 |
| 8 | 2033 | base_plus_full_lactate_dynamics | 2015 | 680 | 0.733 | 0.5887 | 0.1895 | -0.0562 | 0.9121 |
| 8 | 2033 | trajectory_minus_initial_and_clearance | 2015 | 680 | -0.0046 | -0.0033 | 0.0011 | -0.0111 | -0.0072 |
| 9 | 2034 | clinical_base | 2015 | 680 | 0.7025 | 0.5305 | 0.1989 | -0.0307 | 0.9438 |
| 9 | 2034 | base_plus_initial_and_clearance | 2015 | 680 | 0.7267 | 0.5664 | 0.1921 | -0.0337 | 0.937 |
| 9 | 2034 | base_plus_trajectory | 2015 | 680 | 0.7237 | 0.5685 | 0.1928 | -0.0242 | 0.9496 |
| 9 | 2034 | base_plus_full_lactate_dynamics | 2015 | 680 | 0.7342 | 0.5855 | 0.1897 | -0.0467 | 0.9155 |
| 9 | 2034 | trajectory_minus_initial_and_clearance | 2015 | 680 | -0.0031 | 0.0021 | 6.08e-04 | 0.0095 | 0.0126 |
| 10 | 2035 | clinical_base | 2015 | 680 | 0.7037 | 0.526 | 0.199 | -0.031 | 0.9468 |
| 10 | 2035 | base_plus_initial_and_clearance | 2015 | 680 | 0.7287 | 0.5677 | 0.1917 | -0.0316 | 0.9463 |
| 10 | 2035 | base_plus_trajectory | 2015 | 680 | 0.7181 | 0.5625 | 0.1942 | -0.0533 | 0.9144 |
| 10 | 2035 | base_plus_full_lactate_dynamics | 2015 | 680 | 0.7297 | 0.5775 | 0.1911 | -0.074 | 0.8825 |
| 10 | 2035 | trajectory_minus_initial_and_clearance | 2015 | 680 | -0.0107 | -0.0052 | 0.0024 | -0.0217 | -0.0319 |
| 11 | 2036 | clinical_base | 2015 | 680 | 0.7074 | 0.5368 | 0.1977 | -0.0123 | 0.9763 |
| 11 | 2036 | base_plus_initial_and_clearance | 2015 | 680 | 0.7302 | 0.5719 | 0.1911 | -0.0233 | 0.9592 |
| 11 | 2036 | base_plus_trajectory | 2015 | 680 | 0.7271 | 0.5718 | 0.1918 | -0.0184 | 0.9648 |
| 11 | 2036 | base_plus_full_lactate_dynamics | 2015 | 680 | 0.7381 | 0.588 | 0.1888 | -0.0395 | 0.9336 |
| 11 | 2036 | trajectory_minus_initial_and_clearance | 2015 | 680 | -0.0031 | -1.75e-04 | 7.65e-04 | 0.0049 | 0.0056 |
| 12 | 2037 | clinical_base | 2015 | 680 | 0.7078 | 0.5366 | 0.1975 | -0.0133 | 0.9786 |
| 12 | 2037 | base_plus_initial_and_clearance | 2015 | 680 | 0.7313 | 0.5726 | 0.1907 | -0.0223 | 0.9645 |
| 12 | 2037 | base_plus_trajectory | 2015 | 680 | 0.7278 | 0.5754 | 0.1914 | -0.0217 | 0.9691 |
| 12 | 2037 | base_plus_full_lactate_dynamics | 2015 | 680 | 0.7366 | 0.585 | 0.1894 | -0.0527 | 0.9182 |
| 12 | 2037 | trajectory_minus_initial_and_clearance | 2015 | 680 | -0.0035 | 0.0028 | 6.68e-04 | 5.75e-04 | 0.0046 |
| 13 | 2038 | clinical_base | 2015 | 680 | 0.706 | 0.5352 | 0.198 | -0.0183 | 0.9727 |
| 13 | 2038 | base_plus_initial_and_clearance | 2015 | 680 | 0.7309 | 0.5728 | 0.1908 | -0.0178 | 0.9725 |
| 13 | 2038 | base_plus_trajectory | 2015 | 680 | 0.7258 | 0.5694 | 0.1922 | -0.0198 | 0.9659 |
| 13 | 2038 | base_plus_full_lactate_dynamics | 2015 | 680 | 0.7359 | 0.5836 | 0.1895 | -0.0512 | 0.9214 |
| 13 | 2038 | trajectory_minus_initial_and_clearance | 2015 | 680 | -0.005 | -0.0034 | 0.0014 | -0.002 | -0.0066 |
| 14 | 2039 | clinical_base | 2015 | 680 | 0.7068 | 0.5377 | 0.1976 | -0.015 | 0.9773 |
| 14 | 2039 | base_plus_initial_and_clearance | 2015 | 680 | 0.7305 | 0.5719 | 0.1908 | -0.0219 | 0.9646 |
| 14 | 2039 | base_plus_trajectory | 2015 | 680 | 0.7274 | 0.5763 | 0.1912 | -0.019 | 0.9765 |
| 14 | 2039 | base_plus_full_lactate_dynamics | 2015 | 680 | 0.7383 | 0.5915 | 0.1881 | -0.0338 | 0.9475 |
| 14 | 2039 | trajectory_minus_initial_and_clearance | 2015 | 680 | -0.0031 | 0.0044 | 4.17e-04 | 0.0029 | 0.012 |
| 15 | 2040 | clinical_base | 2015 | 680 | 0.7059 | 0.5365 | 0.198 | -0.0222 | 0.9683 |
| 15 | 2040 | base_plus_initial_and_clearance | 2015 | 680 | 0.7302 | 0.5716 | 0.1909 | -0.0254 | 0.9642 |
| 15 | 2040 | base_plus_trajectory | 2015 | 680 | 0.7267 | 0.5713 | 0.1919 | -0.0321 | 0.9577 |
| 15 | 2040 | base_plus_full_lactate_dynamics | 2015 | 680 | 0.7376 | 0.5879 | 0.1887 | -0.0466 | 0.9312 |
| 15 | 2040 | trajectory_minus_initial_and_clearance | 2015 | 680 | -0.0035 | -2.47e-04 | 9.58e-04 | -0.0068 | -0.0065 |
| 16 | 2041 | clinical_base | 2015 | 680 | 0.7043 | 0.5302 | 0.1986 | -0.0223 | 0.9554 |
| 16 | 2041 | base_plus_initial_and_clearance | 2015 | 680 | 0.7292 | 0.5653 | 0.1918 | -0.0305 | 0.9442 |
| 16 | 2041 | base_plus_trajectory | 2015 | 680 | 0.7258 | 0.5692 | 0.1922 | -0.0229 | 0.9593 |
| 16 | 2041 | base_plus_full_lactate_dynamics | 2015 | 680 | 0.7384 | 0.5872 | 0.1889 | -0.0395 | 0.934 |
| 16 | 2041 | trajectory_minus_initial_and_clearance | 2015 | 680 | -0.0034 | 0.0038 | 3.99e-04 | 0.0076 | 0.015 |
| 17 | 2042 | clinical_base | 2015 | 680 | 0.7052 | 0.5355 | 0.1982 | -0.014 | 0.9675 |
| 17 | 2042 | base_plus_initial_and_clearance | 2015 | 680 | 0.7293 | 0.5705 | 0.1912 | -0.0181 | 0.96 |
| 17 | 2042 | base_plus_trajectory | 2015 | 680 | 0.7248 | 0.5712 | 0.1922 | -0.0215 | 0.956 |
| 17 | 2042 | base_plus_full_lactate_dynamics | 2015 | 680 | 0.7363 | 0.5872 | 0.1891 | -0.041 | 0.9302 |
| 17 | 2042 | trajectory_minus_initial_and_clearance | 2015 | 680 | -0.0044 | 6.29e-04 | 9.67e-04 | -0.0034 | -0.004 |
| 18 | 2043 | clinical_base | 2015 | 680 | 0.7051 | 0.5349 | 0.1981 | -0.0195 | 0.9682 |
| 18 | 2043 | base_plus_initial_and_clearance | 2015 | 680 | 0.7291 | 0.5694 | 0.1914 | -0.0295 | 0.9535 |
| 18 | 2043 | base_plus_trajectory | 2015 | 680 | 0.7261 | 0.5757 | 0.1917 | -0.0201 | 0.9686 |
| 18 | 2043 | base_plus_full_lactate_dynamics | 2015 | 680 | 0.7349 | 0.5836 | 0.1895 | -0.0499 | 0.9175 |
| 18 | 2043 | trajectory_minus_initial_and_clearance | 2015 | 680 | -0.003 | 0.0063 | 2.81e-04 | 0.0094 | 0.0151 |
| 19 | 2044 | clinical_base | 2015 | 680 | 0.7059 | 0.5302 | 0.1983 | -0.0262 | 0.9582 |
| 19 | 2044 | base_plus_initial_and_clearance | 2015 | 680 | 0.731 | 0.5711 | 0.191 | -0.0226 | 0.9639 |
| 19 | 2044 | base_plus_trajectory | 2015 | 680 | 0.7255 | 0.5692 | 0.1922 | -0.0199 | 0.962 |
| 19 | 2044 | base_plus_full_lactate_dynamics | 2015 | 680 | 0.7377 | 0.5875 | 0.189 | -0.0444 | 0.9277 |
| 19 | 2044 | trajectory_minus_initial_and_clearance | 2015 | 680 | -0.0055 | -0.0019 | 0.0013 | 0.0027 | -0.0019 |
| 20 | 2045 | clinical_base | 2015 | 680 | 0.7064 | 0.536 | 0.198 | -0.0218 | 0.9626 |
| 20 | 2045 | base_plus_initial_and_clearance | 2015 | 680 | 0.7303 | 0.5736 | 0.1908 | -0.027 | 0.9585 |
| 20 | 2045 | base_plus_trajectory | 2015 | 680 | 0.7264 | 0.5742 | 0.1918 | -0.0183 | 0.9684 |
| 20 | 2045 | base_plus_full_lactate_dynamics | 2015 | 680 | 0.7374 | 0.5881 | 0.1887 | -0.0401 | 0.9359 |
| 20 | 2045 | trajectory_minus_initial_and_clearance | 2015 | 680 | -0.0039 | 5.77e-04 | 9.46e-04 | 0.0086 | 0.0099 |

Note. Twenty 5-fold stratified cross-validation runs used different split seeds. Within every training fold, covariate imputation and scaling, lactate-window preprocessing, K-means fitting, trajectory assignment, and prediction-model fitting were repeated before application to the validation fold. Intervals describe variation across random splits and are not patient-bootstrap confidence intervals. AUROC, area under the receiver operating characteristic curve; AUPRC, area under the precision-recall curve. Source files: Supplementary_Table_S28_Repeated_CV_summary.csv, Supplementary_Table_S28_Repeated_CV_per_repeat.csv.

**Supplementary Table S29. Decision-curve net-benefit variation across repeated cross-validation splits**

| **model** | **threshold** | **mean** | **sd** | **ci95_low_across_splits** | **ci95_high_across_splits** | **minimum** | **maximum** |
| --- | --- | --- | --- | --- | --- | --- | --- |
| base_plus_full_lactate_dynamics | 0.1 | 0.2654 | 7.63e-04 | 0.2641 | 0.2666 | 0.2637 | 0.2667 |
| base_plus_full_lactate_dynamics | 0.15 | 0.2313 | 9.88e-04 | 0.2299 | 0.2332 | 0.2297 | 0.234 |
| base_plus_full_lactate_dynamics | 0.2 | 0.1981 | 0.0014 | 0.1955 | 0.2001 | 0.195 | 0.2001 |
| base_plus_full_lactate_dynamics | 0.25 | 0.17 | 0.0021 | 0.1658 | 0.1739 | 0.1654 | 0.174 |
| base_plus_full_lactate_dynamics | 0.3 | 0.1352 | 0.0026 | 0.1302 | 0.1386 | 0.1293 | 0.1387 |
| base_plus_full_lactate_dynamics | 0.35 | 0.1066 | 0.0031 | 0.0993 | 0.1113 | 0.0992 | 0.1116 |
| base_plus_full_lactate_dynamics | 0.4 | 0.0799 | 0.0025 | 0.0759 | 0.0847 | 0.0759 | 0.0854 |
| base_plus_full_lactate_dynamics | 0.45 | 0.0624 | 0.0017 | 0.0596 | 0.0653 | 0.0595 | 0.0655 |
| base_plus_full_lactate_dynamics | 0.5 | 0.0512 | 0.0023 | 0.0471 | 0.0551 | 0.0471 | 0.0551 |
| base_plus_full_lactate_dynamics | 0.55 | 0.0386 | 0.0041 | 0.0317 | 0.0447 | 0.0315 | 0.0451 |
| base_plus_full_lactate_dynamics | 0.6 | 0.0291 | 0.0033 | 0.0234 | 0.0334 | 0.0233 | 0.0337 |
| base_plus_full_lactate_dynamics | 0.65 | 0.0198 | 0.0035 | 0.0122 | 0.0237 | 0.0112 | 0.0238 |
| base_plus_full_lactate_dynamics | 0.7 | 0.0107 | 0.0019 | 0.0078 | 0.0139 | 0.0073 | 0.0141 |
| base_plus_initial_and_clearance | 0.1 | 0.2644 | 6.79e-04 | 0.2634 | 0.2657 | 0.2634 | 0.2661 |
| base_plus_initial_and_clearance | 0.15 | 0.2303 | 0.001 | 0.2282 | 0.2317 | 0.2282 | 0.2319 |
| base_plus_initial_and_clearance | 0.2 | 0.1964 | 0.0013 | 0.1938 | 0.198 | 0.1937 | 0.1983 |
| base_plus_initial_and_clearance | 0.25 | 0.1681 | 0.0022 | 0.1643 | 0.1711 | 0.1639 | 0.1714 |
| base_plus_initial_and_clearance | 0.3 | 0.1308 | 0.0019 | 0.1272 | 0.1337 | 0.1268 | 0.1343 |
| base_plus_initial_and_clearance | 0.35 | 0.1072 | 0.0026 | 0.1039 | 0.1122 | 0.1038 | 0.1133 |
| base_plus_initial_and_clearance | 0.4 | 0.081 | 0.0021 | 0.0763 | 0.0833 | 0.0751 | 0.0834 |
| base_plus_initial_and_clearance | 0.45 | 0.0533 | 0.002 | 0.0494 | 0.0562 | 0.0492 | 0.0563 |
| base_plus_initial_and_clearance | 0.5 | 0.0463 | 0.0032 | 0.0402 | 0.0499 | 0.0402 | 0.0506 |
| base_plus_initial_and_clearance | 0.55 | 0.037 | 0.0026 | 0.0317 | 0.0414 | 0.0302 | 0.0421 |
| base_plus_initial_and_clearance | 0.6 | 0.0301 | 0.0014 | 0.0277 | 0.032 | 0.0275 | 0.0323 |
| base_plus_initial_and_clearance | 0.65 | 0.0164 | 0.0023 | 0.0137 | 0.0213 | 0.0133 | 0.0225 |
| base_plus_initial_and_clearance | 0.7 | 0.0053 | 0.0023 | 0.0021 | 0.0093 | 0.002 | 0.0094 |
| base_plus_trajectory | 0.1 | 0.2652 | 8.20e-04 | 0.264 | 0.2669 | 0.2639 | 0.2669 |
| base_plus_trajectory | 0.15 | 0.2294 | 7.21e-04 | 0.228 | 0.2306 | 0.2277 | 0.2307 |
| base_plus_trajectory | 0.2 | 0.1939 | 0.0012 | 0.1916 | 0.1952 | 0.1913 | 0.1952 |
| base_plus_trajectory | 0.25 | 0.1645 | 0.0017 | 0.1615 | 0.1673 | 0.1613 | 0.1674 |
| base_plus_trajectory | 0.3 | 0.1352 | 0.0017 | 0.1317 | 0.1373 | 0.1308 | 0.1373 |
| base_plus_trajectory | 0.35 | 0.0993 | 0.0024 | 0.0941 | 0.1029 | 0.0937 | 0.1032 |
| base_plus_trajectory | 0.4 | 0.0748 | 0.0024 | 0.0696 | 0.0784 | 0.0677 | 0.0794 |
| base_plus_trajectory | 0.45 | 0.0591 | 0.0021 | 0.0543 | 0.0618 | 0.0539 | 0.0618 |
| base_plus_trajectory | 0.5 | 0.0435 | 0.0016 | 0.0414 | 0.0464 | 0.0412 | 0.0467 |
| base_plus_trajectory | 0.55 | 0.0336 | 0.0027 | 0.0287 | 0.0381 | 0.0286 | 0.0395 |
| base_plus_trajectory | 0.6 | 0.0235 | 0.0028 | 0.0181 | 0.0279 | 0.0176 | 0.028 |
| base_plus_trajectory | 0.65 | 0.0152 | 0.0019 | 0.0117 | 0.0187 | 0.011 | 0.0196 |
| base_plus_trajectory | 0.7 | 0.0055 | 0.003 | 5.67e-04 | 0.0098 | 3.31e-04 | 0.0099 |
| clinical_base | 0.1 | 0.2635 | 4.95e-04 | 0.2628 | 0.2641 | 0.2628 | 0.2641 |
| clinical_base | 0.15 | 0.2267 | 9.84e-04 | 0.225 | 0.228 | 0.2249 | 0.2282 |
| clinical_base | 0.2 | 0.193 | 0.0014 | 0.1907 | 0.1951 | 0.1903 | 0.1954 |
| clinical_base | 0.25 | 0.1618 | 0.0017 | 0.1584 | 0.164 | 0.1578 | 0.1641 |
| clinical_base | 0.3 | 0.1271 | 0.0018 | 0.1238 | 0.1301 | 0.1234 | 0.1309 |
| clinical_base | 0.35 | 0.0921 | 0.0018 | 0.0892 | 0.0945 | 0.0888 | 0.0947 |
| clinical_base | 0.4 | 0.0643 | 0.0023 | 0.0612 | 0.0687 | 0.0607 | 0.0693 |
| clinical_base | 0.45 | 0.0399 | 0.002 | 0.0368 | 0.0433 | 0.0362 | 0.0437 |
| clinical_base | 0.5 | 0.0265 | 0.0024 | 0.0221 | 0.0313 | 0.0218 | 0.0328 |
| clinical_base | 0.55 | 0.0155 | 0.0025 | 0.0115 | 0.0192 | 0.0114 | 0.0193 |
| clinical_base | 0.6 | 0.008 | 0.0022 | 0.0047 | 0.0117 | 0.0037 | 0.0122 |
| clinical_base | 0.65 | 0.0047 | 0.0025 | 6.38e-04 | 0.0086 | 6.38e-04 | 0.0086 |
| clinical_base | 0.7 | 0.0029 | 0.0024 | -0.0014 | 0.0068 | -0.0022 | 0.0079 |

Note. Net benefit was calculated from out-of-fold predictions in each of twenty repeated 5-fold cross-validation runs. The reported mean, standard deviation, interval, minimum, and maximum summarize split-to-split variation at each threshold probability. Source files: Supplementary_Table_S29_Repeated_CV_decision_curve.csv.
